# Joint effect of modifiable risk factors on Parkinson’s disease: a large-scale longitudinal study

**DOI:** 10.3389/fnhum.2025.1525248

**Published:** 2025-01-27

**Authors:** Panlong Li, Xirui Zhu, Min Liu, Yanfeng Wang, Chun Huang, Junwei Sun, Shan Tian, Yuna Li, Yuan Qiao, Junting Yang, Shanshan Cao, Chaohua Cong, Lei Zhao, Jingjing Su, Dandan Tian

**Affiliations:** ^1^School of Electrical and Information Engineering, Zhengzhou University of Light Industry, Zhengzhou, China; ^2^Department of Hypertension, Henan Provincial People’s Hospital, Zhengzhou University People’s Hospital, Zhengzhou, China; ^3^Department of Neurology, Shanghai Ninth People’s Hospital, Shanghai Jiao Tong University School of Medicine, Shanghai, China

**Keywords:** Parkinson’s disease, modifiable risk factors, population attributable fraction, physical measurement, lifestyle, medical history

## Abstract

**Introduction:**

Previous researches have often underestimated the diversity and combined effects of risk factors for Parkinson’s disease (PD). This study aimed to identify how multiple modifiable risk factors collectively impact PD.

**Methods:**

The study included 452,492 participants from the UK Biobank, utilizing genetic data and 255 phenotypic variables. A broad exposure association study was conducted across seven domains: socioeconomic status, medical history, psychosocial factors, physical measures, early life, local environment, and lifestyle. Risk scores of each domain for each participant were generated. The joint effects of modifiable and genetic risks assessed using Cox proportional hazards model. Population attributable fraction (PAF) was estimated to quantify contribution ratio of risk factors in different domains to the occurrence of PD.

**Results:**

Multiple risk factors significantly (*p* < 1.96 × 10^−4^) associated with PD was observed. The top 5 factors were hand grip strength (hazard ratio (HR) = 0.98, *p* = 1.59 × 10^−24^), long-standing illness (HR = 1.38, *p* = 3.63 × 10^−20^), self-reported nervousness (HR = 1.56, *p* = 5.9 × 10^−20^), ever suffered from mental health concerns (HR = 1.42, *p* = 5.48 × 10^−18^) and chest pain (HR = 1.42, *p* = 1.43 × 10^−18^). Individuals with unfavorable medical history, psychosocial factors, physical measures, and lifestyle had an increased risk of PD by 33 to 51% compared to those with favorable factors (*p* < 0.001).

**Discussion:**

Results indicated that addressing modifiable risk factors, especially in physical measures and psychological factors, could potentially prevent up to 33.87% of PD cases. In formulating prevention strategies, it is recommended to prioritize domains such as physical measures, psychosocial factors, lifestyle, and medical history.

## 1 Introduction

Parkinson’s disease (PD) is a neurodegenerative disorder that primarily impacts motor functions, including tremor, muscle stiffness, slowness of movement, and postural balance disorders (Isik et al., 2023). About 10 million people worldwide suffer from PD, and this number is projected to ascend as the population ages (Mahmood et al., 2023). Currently, the precise etiology of PD remains incompletely understood (Ben-Shlomo et al., 2024). Moreover, there is no cure for PD, and the treatment primarily revolves around alleviating symptoms and enhancing the patient’s quality of life (Ma et al., 2023). Therefore, effective prevention strategies before the onset of the disease have become an important aspect of reducing the burden of PD.

Existing research has made some progress in exploring the modifiable risk factors of PD, but there are also limitations. Studies typically employ hypothesis-driven methods, focusing on one or a few risk factors, without fully considering the potential regulatory effects of other risk factors (Costa et al., 2020; Lee et al., 2021; Tchekalarova and Tzoneva, 2023). This limitation may result in an overestimation of the effect size, thereby affecting the accurate assessment of disease risk (Lin et al., 2022). Furthermore, single-factor analyses have failed to elucidate the contribution of each factor to the total burden of PD (Takeuchi and Kawashima, 2022; Larsson and Burgess, 2022). This limitation means that we may not fully understand the role of each individual risk factor in the development of the disease, and how they interact with other factors to jointly affect the progression and severity of the disease (Ben-Shlomo et al., 2024). Consequently, previous research has often overlooked the synergistic effects among various factors, failing to fully capture their combined impact on disease risk (Hahad et al., 2020; Brett et al., 2022). Therefore, it remained unclear what would happen to PD if we simultaneously reversed these risk factors. In summary, these limitations hinder our ability to develop effective targeted prevention strategies. To overcome these limitations, more comprehensive and systematic research methods are needed.

The exposure-wide association study (EWAS) does not rely on specific hypotheses, but adopts an open approach to explore multiple possible risk factors. This method has been successfully applied to the study of depression (Choi et al., 2020) and dementia (Zhang et al., 2023). It reduces the bias and preconceived notions that can lead to selective analysis (Manrai et al., 2017), enabling the identification of previously unconsidered or under-researched risk factors (Zhuang et al., 2018), offering synthesis information for disease’s prevention and treatment. Based on identified risk factors in EWAS, Population Attributable Fraction (PAF) estimation helps to quantify overall contribution of these factors to Parkinson’s prevalence, which provides scientific support for the formulation of public health strategies and interventions (Mukadam et al., 2019).

In this study, we leveraged data from over 400,000 participants in the UK Biobank, employing extensive exposure-association analyses. We identified risk factors associated with PD and integrated these factors into composite risk scores across various domains, aiming to assess their joint effect on the disease. Furthermore, we calculated the PAF to quantify the preventive efficacy that could be achieved by reversing these risk factors.

## 2 Methods

### 2.1 Study populations and ascertainment of Parkinson’s disease

**Figure 1:**
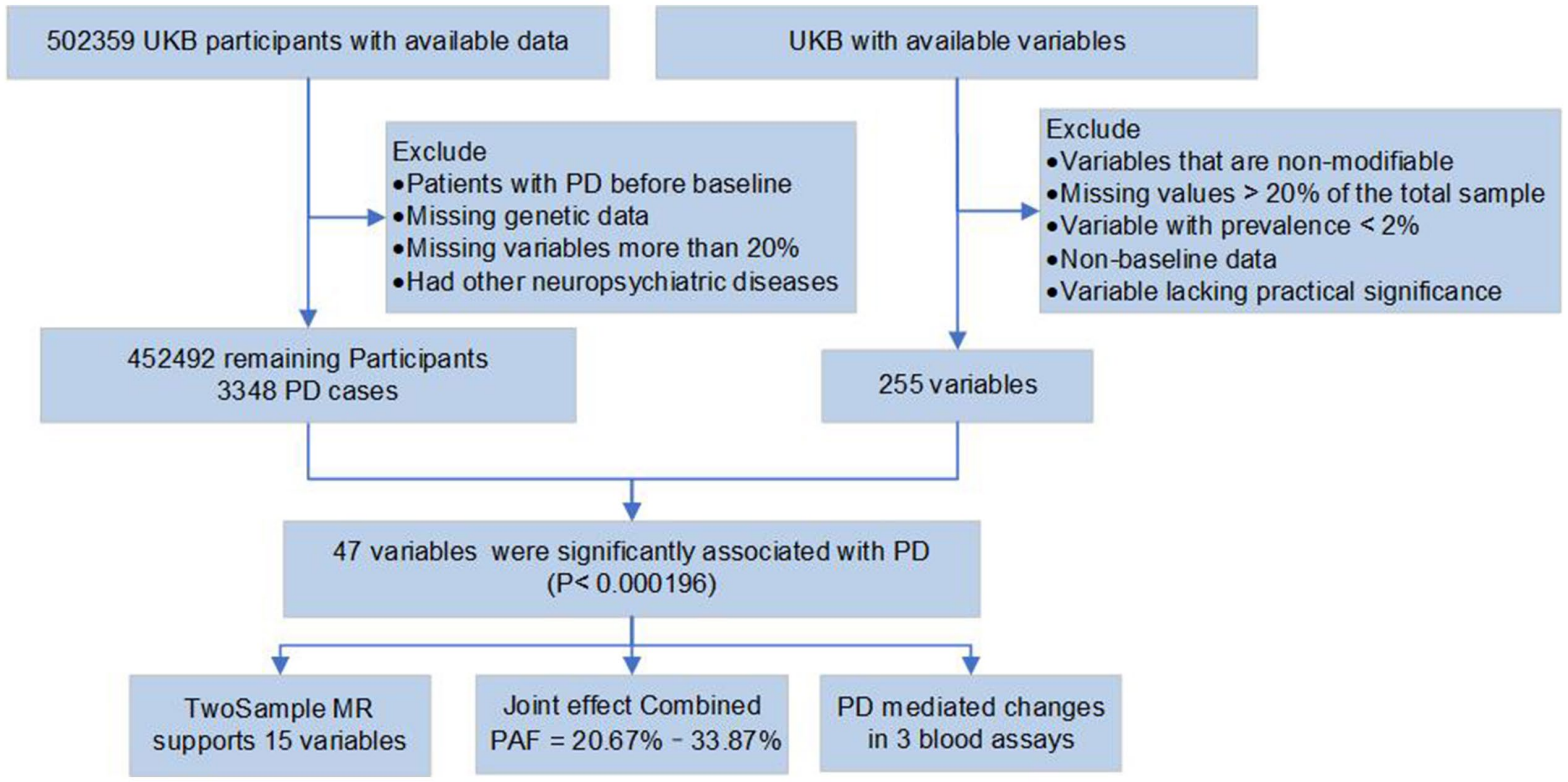
Work flow for the participants selection and study design. PD, Parkinson’s disease; TwoSample MR, 2-sample Mendelian randomization; PAF, population attributable fraction.

The dataset utilized in our research originates from the UK Biobank, which conducted detailed baseline assessments on over half a million British, aged 40 to 69, across 22 assessment centers (Bycroft et al., 2018). It records participants’ health data (including information from hospital health systems and self-reported data by participants), biological samples, imaging and genetic information, as well as multi-dimensional information such as demographic data, socioeconomic status, and lifestyle. We excluded participants diagnosed with PD prior to the baseline assessment, those missing genetic data, and those with more than 20% missing variable, and additionally, those with other neuropsychiatric diseases such as dementia, stroke, and depression. The final analysis included 452,492 participants. The diagnoses of PD were derived from hospitalization records, death registration, healthcare records, and self-reports. PD status was confirmed using the ICD-10 code G20 from the hospital registry data (Supplementary Table 1). STROBE Collaborative Reporting guidelines were used in this study (von Elm et al., 2014). Figure 1 gave out the study design of this study. The approval number of the UK Biobank is 94,885.

### 2.2 Modifiable variables

From the exposure variables available in UK Biobank, we excluded those that were unmodifiable, those with more than 20% missing values, and those with no real meaning. During the variable processing process, we recoded meaningful negative values (e.g., −10 indicating less than 1, we recoded it to 0.5) and non-meaningful negative values (e.g., −1 indicating “Do not know,” −3 indicating “Prefer not to answer,” we recoded them to null values). For missing values, we used the mode to fill in missing values in categorical variables and the median to fill in missing values in continuous variables. For categorical variables, we recoded them in a logical order and used dummy coding to convert multiple choice categorical variables into binary variables. Additionally, for variables measured at multiple time points, we calculated their average values. A detailed description of variable processing was provided in the supplementary Note. We collected data on 255 modifiable variables measured at baseline. These factors were categorized into seven domains: socioeconomic status, medical history, psychosocial factors, physical measures, early life, local environment, and lifestyle. The names of these variables and their field IDs in the UK Biobank provided in Supplementary Table 2.

### 2.3 Calculation of the polygenic risk scores

The calculation of PRS for PD was based on a clustering subset of SNPs that exceeded a particular threshold for the *p*-value in GWAS (Choi et al., 2020). To estimate the genetic risk of each participant, we calculated the PRS using the following formula: $PRS_{j}=\left[\overset{n}{\underset{i=1}{\sum }}\left(\beta_{i}\times G_{ij}\right)-\mathrm{Mean}\left(PRS\right)\right]/SD\left(PRS\right)$ , i corresponds to the ith SNP, j corresponds to the jth individual, *β* denotes the statistical coefficient for that SNP, and G stands for the number of observed effective alleles. The calculation of PRS was detailed in Supplementary Note.

### 2.4 Blood assays source

The blood assays we used in this study were sourced from the UK Biobank. At the time of initial recruitment, blood assays were collected from participants and analyzed at the UK Biobank central laboratory. The assays included various blood cell counts such as monocytes, neutrophils, hematocrit, hemoglobin, red blood cells, lymphocytes, white blood cells, platelets, and mean corpuscular hemoglobin volume. A series of key blood biochemical markers were also collected: C-reactive protein, cholesterol, rheumatoid factor, total bilirubin, etc.

### 2.5 Statistical analyses

We utilized Cox proportional hazards models to comprehensively examine the associations between individual exposure variables and PD events, adjusting for age, gender and assessment center. A conservative Bonferroni correction method was applied to determine the significance threshold (1.96 × 10^−4^). This was calculated by dividing the conventional significance level of 0.05 by the number of modifiable variables collected at baseline (255). We tested the hazard proportional assumption by analyzing the relationship between follow-up time and standardized Schoenfeld residuals. In cases where the hazard proportional assumption was violated, an interaction term for follow-up time was introduced into the model (Ganna and Ingelsson, 2015). We further conducted a multicollinearity analysis among the significant variables, to exclude those with a high correlation (*r*^2^ > 0.9). In sensitivity analyses, we replicated the above analysis stratified by age (≥65/<65), gender (male/female), and PRS (high/medium/low) to examine potential differences among different subgroups.

Two-sample Mendelian Randomization (MR) analyses were conducted to further explore the causal associations between risk factors and PD. We obtained the GWAS statistics of significant variables from IEU Open GWAS Project (https://gwas.mrcieu.ac.uk/). The selection of traits for MR analysis was provided in Supplementary Note. We selected the GWAS dataset of ieu-b-7, which boasts the largest sample size, comprising a total of 482,730 Europeans (including 33,674 cases and 449,056 controls). We identified significant single nucleotide polymorphisms (SNPs) as instrumental variables, with *p*-value < 5 × 10^−8^ and a linkage disequilibrium r^2^ < 0.001. In cases where traits had an insufficient number of SNPs after removing outliers, we adjusted the p-value threshold to 5 × 10^−6^ to increase the SNP’s count (Choi et al., 2020). MR analysis primarily utilized the inverse variance weighted (IVW) method, supplemented by weighted median method and MR-Egger regression analysis (Bowden et al., 2016; Bowden et al., 2015) as additional approaches. We performed Cochran’s Q test, MR-Egger intercept and the MR-PRESSO global test, to examine potential heterogeneity and horizontal pleiotropy. These MR analyses were executed utilizing the “TwoSampleMR” package in the R software, with the version used being 4.3.0. The study adheres to the STROBE-MR guidelines.

We categorized variables which significantly associated with PD into five domains: medical history, lifestyle, psychosocial factors, socioeconomic status, and physical measures and calculated the risk score for each domain. In the analysis, factors with protective effects (hazard ratio: HR < 1) were recoded as risk factors, and assigned a score of 1. Participants scored 1 point for each risk factor they possess and were summed to arrive at unweighted risk scores for each domain. To calculate the weighted risk score for each domain, we employed the Cox regression models, incorporating only the risk factors within the same domain in each analysis. The multi-variables Cox regression model adjusted for baseline age, gender, and assessment center was applied to estimate the combined, yielding *β*-coefficients for each variable. Each of initial binary variables was multiplied by its respective *β*-coefficient, the products were added together, and then divided by the aggregate of β-coefficients to generate normalized weighted scores for each domain. The specific formula for calculating the weighted risk score is as follows: $S=\overset{N}{\underset{k=1}{\sum }}\left(X_{K}\cdot \beta_{K}\right)/\overset{N}{\underset{K=1}{\sum }}\beta_{K}$ , where X_k_ is the K_th_ variable, β_k_ is the β-coefficient of the K_th_ risk factor, and N is the total number of risk factors in the domain. Higher scores indicated a greater level of risk exposure for individuals. We further categorized the scores into three levels: favorable, moderate, and unfavorable to elucidate the risk profile.

To estimate the joint effect of multiple risk factors from different domains, multiple Cox regression models were applied. First, we examined the association between a single modifiable risk score and PD adjusted for age, gender, and assessment center. Then, we further combined five modifiable risk scores in one Cox regression model to estimate their combined effect. Additionally, we conducted the same Cox regression analyses as before using unweighted scores, to further verify the robustness of the results.

To test whether genetic predisposition influences the relationship between risk scores and PD, we stratified participants into nine groups based on their PRS (low, medium, high) and the tertiles of the risk score in each domain. We then assessed the HR for PD within each group, while adding genotype measurement batches to the original Cox model as covariates in the analysis.

To explore the effect of these exposures on PD and blood assays, mediation analyses were further conducted. Two sets of mediation models were applied: one set aimed to determine whether modifiable risk scores mediated changes in blood assays via PD as the intermediary variable; the other set to examine the potential mediating effects of blood assays between modifiable risk scores and the PD development. For variables that demonstrated significant mediation effects, we further explored potential causal effects using MR. The method used for MR is consistent with the approach described earlier for exploring the causal relationship between risk factors and PD.

The PAF quantifies the proportion of expected reduction in disease if a specific risk factor was eliminated or reduced. Two different PAF calculation methods were applied to provide different perspectives on the potential for disease reduction by eliminating or reducing risk factors. In the first method, we constructed a conservative model that eliminated only unfavorable factors within each domain. In the second method, we developed a more optimistic model that excluded both unfavorable and moderate factors in each domain. Using the Cox regression model adjusted for age, gender, and assessment center, we calculated the HR for each domain. Then, we generated PAFs for each domain using the formula: $PAF=\left(P_{\mathrm{pop}}\times \left(HR-1\right)\right)/\left[P_{\mathrm{POP}}\times \left(HR-1\right)+1\right]$ , where Ppop represents the exposure rates in the total population. Principal component analysis was applied to estimate the weights for each PAF, which represented the commonality among the various domains. Both weighted PAFs for each domain and an overall weighted PAF were calculated.

## 3 Results

**Figure 2:**
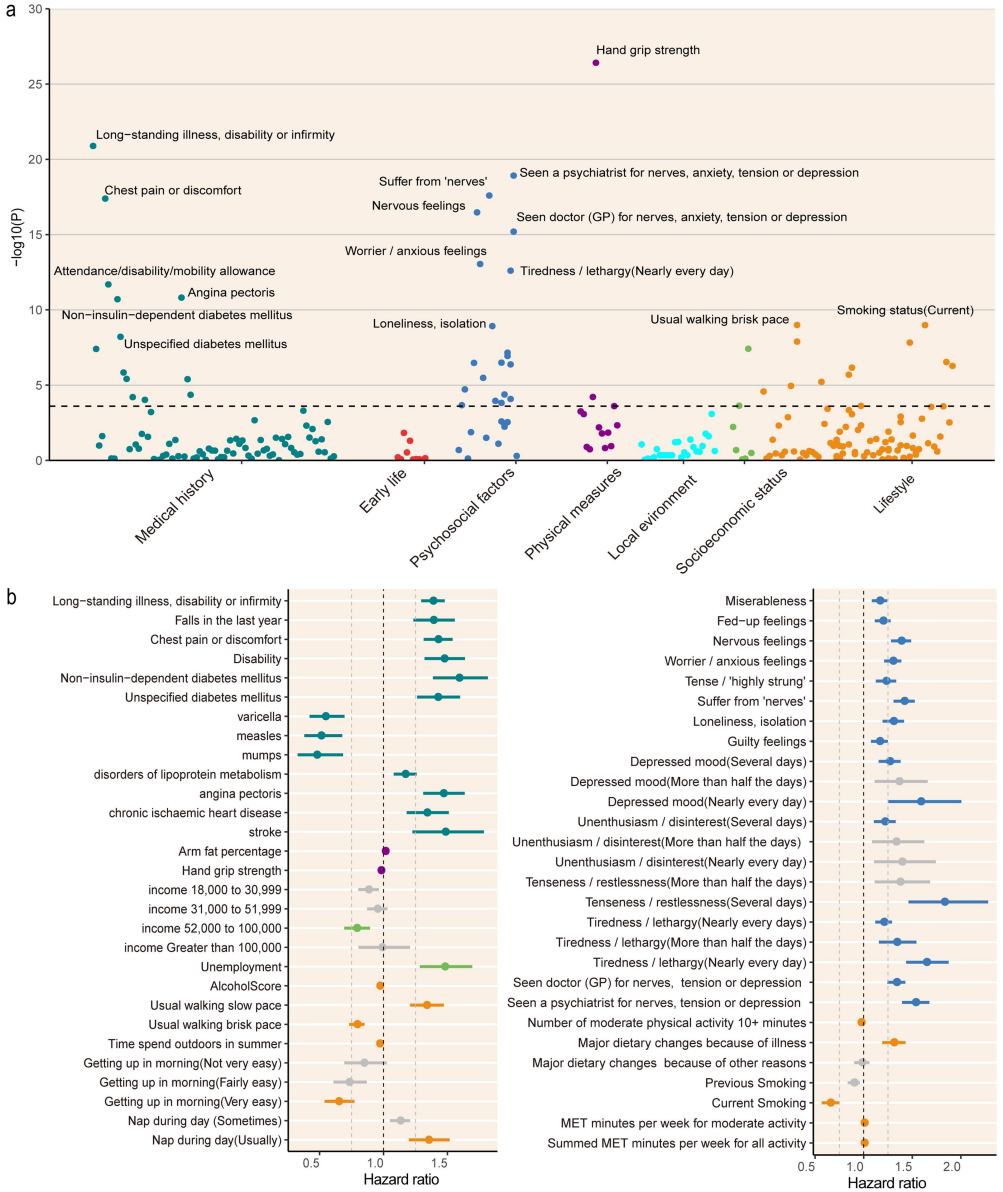
Associations between modifiable risk factors and Parkinson’s disease. **(A)** The x-axis represents categorical domains, while the y-axis represents statistical significance in the form of the negative logarithm (−log10) of the *p*-value. The dashed line denotes the adjusted threshold post-Bonferroni correction (1.96 × 10^−4^). **(B)** Each point represents a hazard ratio, accompanied by a horizontal line indicating the corresponding 95% confidence interval. Hazard ratios were calculated through a Cox proportional hazards regression analysis, with adjustments made for baseline age, gender, and assessment center. Grey means not significant after Bonferroni−corrected.

The study cohort included 52.9% females, with a mean age of 56.53 years and a standard deviation (SD) of 8.12. Among the 452,492 participants, 3,348 were diagnosed with PD at an average age of 62.85 years (SD = 5.34) (baseline characteristics were provided in the Supplementary Table 3 and Supplementary note). We identified 47 out of 255 modifiable factors significantly associated with PD (see Figure 2; Supplementary Table 4). Among these, 36 factors exhibited potential adverse impact, while the remaining 11 displayed protective effects. The three primary factors contributing to an elevated PD risk were long-standing illness (HR = 1.38, 95%CI = 1.29–1.48, *p* = 3.63 × 10^−20^), self-reported nervousness (HR = 1.56, 95%CI = 1.42–1.71, *p* = 5.9 × 10^−20^), and ever suffered from mental health concerns (HR = 1.42, 95%CI = 1.31–1.54, *p* = 5.48 × 10^−18^). Conversely, the three main factors that lowered PD risk were a strong hand grip (HR = 0.98, 95%CI = 0.97–0.98, *p* = 1.59 × 10^−24^), current smoking (HR = 0.66, 95%CI = 0.58–0.76, *p* = 9.8 × 10^−9^), and a brisk usual walking pace (HR = 0.80, 95%CI = 0.74–0.86, *p* = 1.53 × 10^−8^).

**Figure 3:**
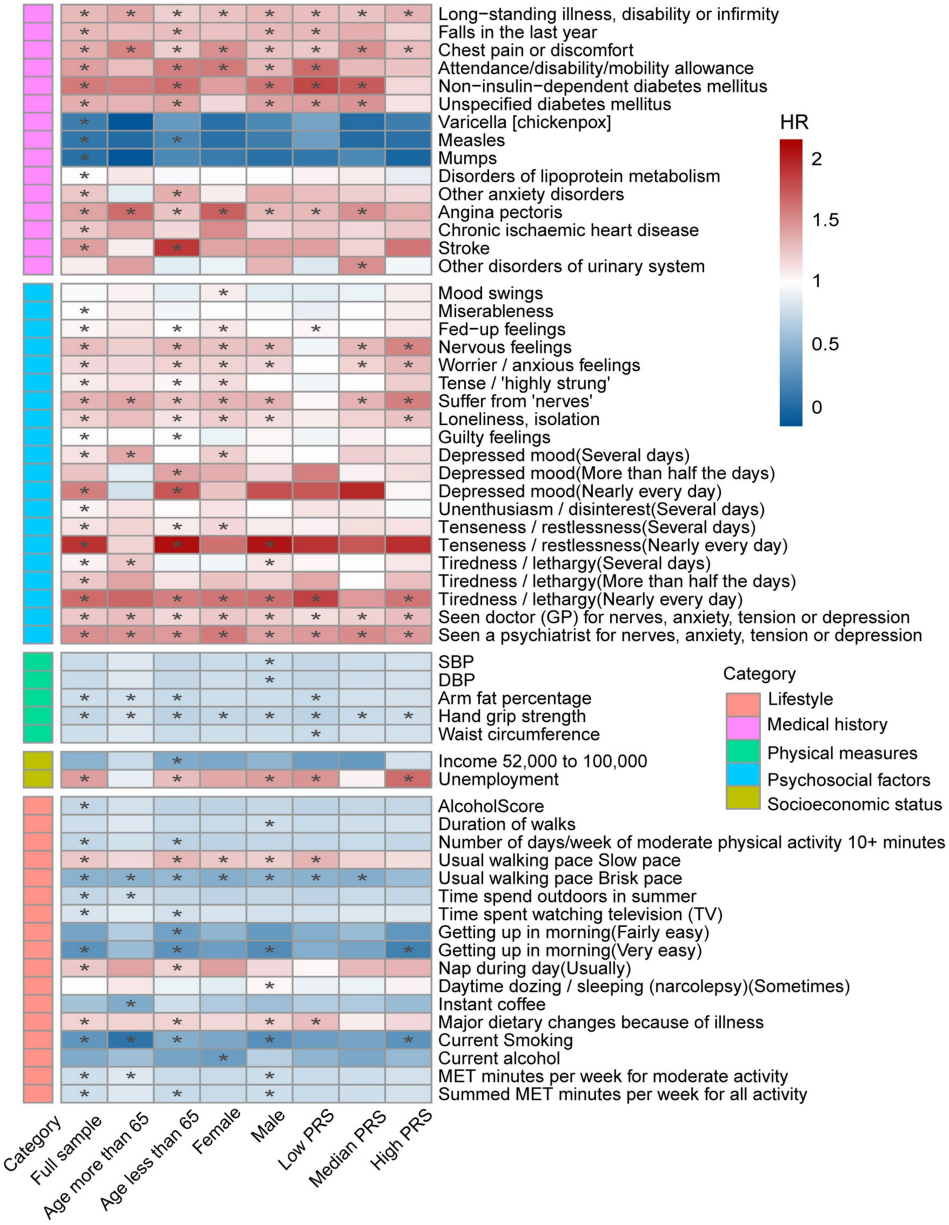
Heatmap for significant factors associated with Parkinson’s disease (Full sample and by age, gender and PRS). The color within each box represents hazard ratio, with adjustments for baseline age, gender, and assessment center in the model. Asterisks denote statistical significance after applying the Bonferroni correction (1.96 × 10^−4^).

After applying Bonferroni correction, early life and local environment factors were not found to be statistically significant. The results of individual exposure across subgroups categorized by age, gender, and PRS revealed similar patterns of association (Figure 3; Supplementary Table 4). In the collinearity analysis, no factors with high correlation were identified that required exclusion. The correlations among the significant variables were presented in Supplementary Figure 1.

Mendelian randomization uncovered 15 potential factors that have a causal relationship with PD (Supplementary Tables 5, 6). These factors specifically included: arm fat percentage for both right and left sides [respective odds ratio (OR) = 0.75, 0.76, *p* = 0.0015, 0.0022], diabetes (OR = 1.06, *p* = 0.0047), long-standing illness (OR = 5.35, *p* = 0.0148), experiencing falls in the last year (OR = 3.24, *p* = 0.0235), four lifestyle factors (current smoking: OR = 0.14, *p* = 0.0009; walking pace: OR = 2.8, *p* = 0.0031; moderate activity: OR = 0.65, *p* = 0.022; and getting up in the morning: OR = 0.6, *p* = 0.0379), and six psychosocial factors, such as anxious, worrier and guilty feelings (ORs ranging from 2.69 to 8.13, *p*-values between 0.0059 and 0.0471). Supplementary Table 6 listed the results obtained from the IVW and additional methods. The MR Egger failed to reveal any heterogeneity or horizontal pleiotropy.

**Figure 4:**
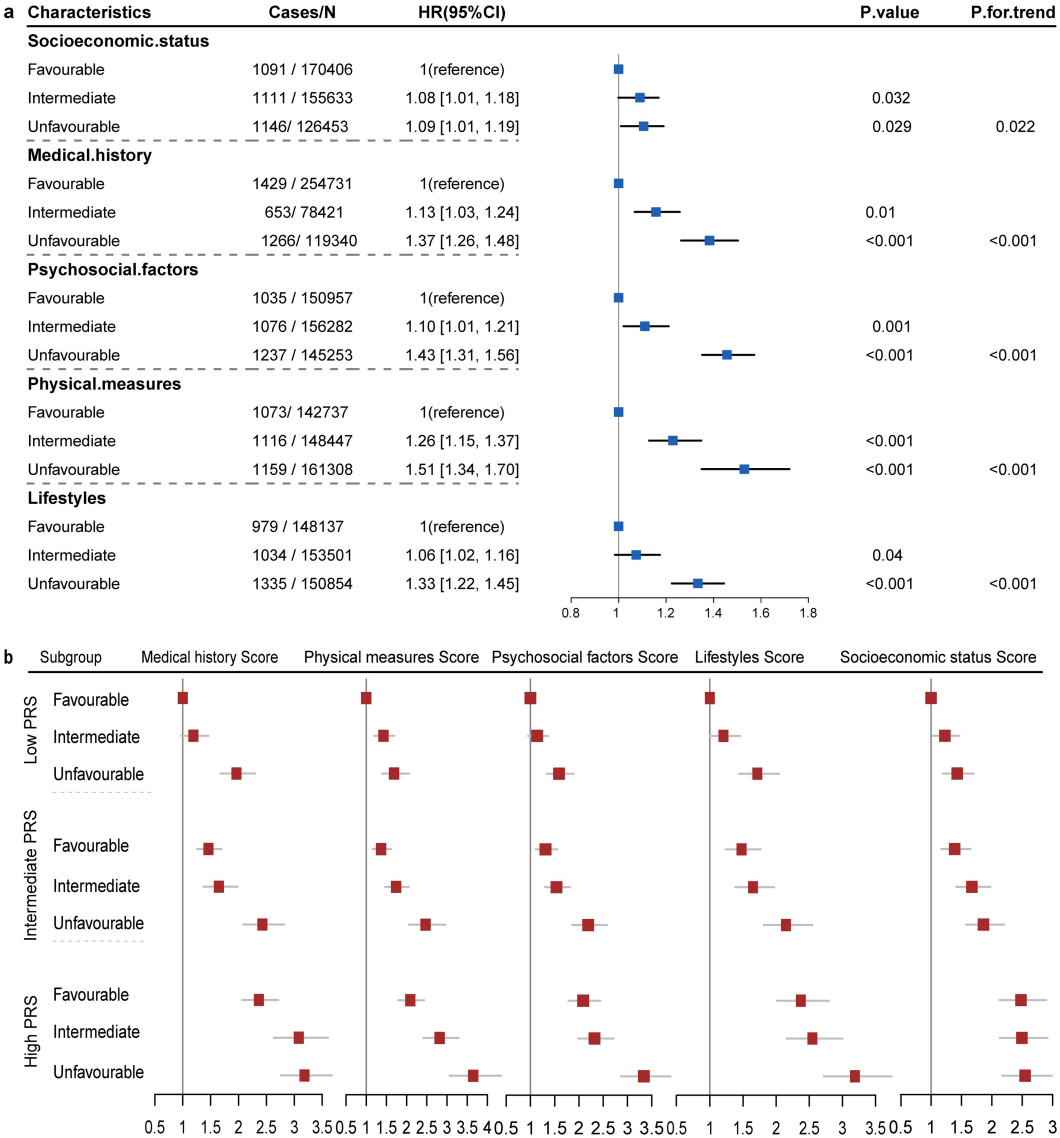
Association between weighted scores in five domains and Parkinson’s disease. **(A)** Model adjusted for age, sex, assessment center, and the scores in the five different areas. **(B)** Risk of Parkinson’s disease was stratified within each genetic risk category.

Compared to favorable characteristics, moderate factors in socioeconomic status, medical history, psychosocial factors, physical measures, and lifestyle increased the risk of PD by 6–26% (*p* < 0.05) (Figure 4). The unfavorable factors in the five domains were significantly linked to a higher PD risk, low socioeconomic status increased the PD risk by 9% (*p* = 0.029), lifestyle and medical history each contributed to a 33 and 37% increased risk (*p* < 0.001), with psychosocial factors and physical measures leading to a 43 and 51% increase in PD risk, respectively, (*p* < 0.001). Sensitivity analysis demonstrated that the impact of unweighted score on PD risk remained consistent (Supplementary Table 7).

Notably, there were marked statistical interactions between modifiable risk scores and PRS (Figure 4). Participants with both high genetic risk and modifiable risk scores had 155 to 265% greater risk of PD than participants with low genetic risk and modifiable risk scores (*p* < 0.001). Furthermore, based on genetic risk stratification, favorable profiles of four domains were tied to reduced risk of PD across different genetic categories (Table 1). In individuals with higher PRS, favorable profiles of physical measures, psychosocial factors, medical history, and lifestyle were significantly related to a lower incidence of PD, with HRs ranging from 0.62 to 0.75, *p*-values between 3.1 × 10^−13^ and 3.8 × 10^−6^.

**Table 1:** Association between five domains and Parkinson’s disease within each genetic risk category.

	Low genetic risk		Moderate genetic risk		High genetic risk	
	HR (95% CI)	*P*	HR (95% CI)	*P*	HR (95% CI)	*P*
Socioeconomic status						
Favorable	0.71 (0.59–0.84)	0.00011	0.74 (0.64–0.87)	0.00015	0.97 (0.86–1.10)	0.646
Intermediate	0.87 (0.73–1.03)	0.10416	0.90 (0.78–1.04)	0.16654	0.97 (0.86–1.10)	0.672
Unfavorable	1.00	Reference	1.00	Reference	1.00	Reference
Medical history						
Favorable	0.52 (0.44–0.61)	3.9 × 10^−16^	0.60 (0.52–0.69)	1.9 × 10^−13^	0.74 (0.66–0.83)	1.5 × 10^−7^
Intermediate	0.61 (0.51–0.72)	1.1 × 10^−6^	0.68 (0.57–0.81)	1.3 × 10^−5^	0.97 (0.84–1.11)	0.635
Unfavorable	1.00	Reference	1.00	Reference	1.00	Reference
Psychosocial factors						
Favorable	0.61 (0.51–0.72)	2.1 × 10^−8^	0.60 (0.52–0.70)	4.6 × 10^−11^	0.63 (0.56–0.72)	3.1 × 10^−13^
Intermediate	0.71 (0.59–0.84)	6.8 × 10^−5^	0.70 (0.61–0.81)	2.8 × 10^−6^	0.70 (0.62–0.79)	5.3 × 10^−9^
Unfavorable	1.00	Reference	1.00	Reference	1.00	Reference
Physical measures						
Favorable	0.52 (0.41–0.66)	1 × 10^−7^	0.55 (0.45–0.68)	3 × 10^−8^	0.62 (0.52–0.73)	5 × 10^−8^
Intermediate	0.76 (0.62–0.94)	0.01075	0.70 (0.58–0.84)	0.00013	0.82 (0.70–0.94)	0.00064
Unfavorable	1.00	Reference	1.00	Reference	1.00	Reference
Lifestyles						
Favorable	0.57 (0.48–0.68)	5.2 × 10^−10^	0.69 (0.59–0.80)	1.6 × 10^−6^	0.75 (0.67–0.85)	3.8 × 10^−6^
Intermediate	0.70 (0.59–0.83)	3 × 10^−5^	0.77 (0.66–0.89)	0.00048	0.80 (0.71–0.90)	0.00029
Unfavorable	1.00	Reference	1.00	Reference	1.00	Reference

The findings of the mediation analysis were presented in Table 2. The analysis revealed that modifiable risk scores in the four domains of medical history, psychosocial factors, socioeconomic status, and lifestyles were correlated with both the lowered lymphocyte percentage and the raised neutrophil percentage, attributed in part to PD’s mediating effect (Mediation effect size ranging from 0.001 to 0.002, *p* < 0.001). The study also found that PD mediated the relationship between lifestyle scores and total bilirubin levels, which could increase total bilirubin (Mediation effect size = 0.001, *p* < 0.001). The MR analysis supported three blood assays: lymphocyte percentage (OR = 0.84, *p* = 0.03), neutrophil percentage (OR = 1.12, *p* = 0.02), and total bilirubin (OR = 1.10, *p* = 0.04) (Supplementary Table 8).

**Table 2:** Analysis of mediating effect of Parkinson’s disease on changes of blood assays.

Exposure	Mediator	Outcome	Total effect	Direct effect	Mediation effect	
			Effect size [95% CI]	Effect size [95% CI]	Effect size [95% CI]	*p*
Medical history score	PD	Lymphocyte percentage	−0.481 [−0.506, −0.453]	−0.479 [−0.505, −0.451]	−0.002 [−0.002, −0.001]	0.001 ***
		Neutrophil percentage	0.450 [0.416, 0.478]	0.448 [0.414, 0.476]	0.002 [0.002, 0.003]	0.001 ***
Psychosocial factors score	PD	Lymphocyte percentage	−0.171 [−0.185, −0.154]	−0.170 [−0.184, −0.153]	−0.001 [−0.001, −0.001]	0.001 ***
		Neutrophil percentage	0.194 [0.177, 0.209]	0.193 [0.176, 0.208]	0.001 [0.001, 0.001]	0.001 ***
Socioeconomic status score	PD	Lymphocyte percentage	−0.464 [−0.499, −0.426]	−0.463 [−0.498, −0.425]	−0.001 [−0.002, −0.001]	0.001 ***
		Neutrophil percentage	0.593 [0.553, 0.629]	0.592 [0.552, 0.628]	0.001 [0.001, 0.002]	0.001 ***
Lifestyles score	PD	Lymphocyte percentage	−0.049 [−0.059, −0.041]	−0.048 [−0.058, −0.040]	−0.001 [−0.002, −0.001]	0.001 ***
		Neutrophil percentage	0.031 [0.022, 0.043]	0.030 [0.022, 0.042]	0.001 [0.001, 0.001]	0.001 ***
		Total bilirubin	0.057 [0.052, 0.062]	0.056 [0.052, 0.061]	0.001 [0.000, 0.001]	0.001 ***

PD, Parkinson’s disease; CI, confidence interval.

The PAF assessment revealed that by transforming individual unfavorable profiles to moderate or favorable conditions (Model 1), a potential prevention of 20.67% of PD cases could be achieved. Further optimization to favorable states for all factors (Model 2), the PAF suggested an increase in the prevention rate to 33.87% (Table 3; Supplementary Tables 9, 10). In an ideal scenario where these risk factors could be completely eliminated, physical measures demonstrated the greatest potential for prevention, attributing to a 10.62% reduction in incidence. Following closely were psychosocial factors (9.34%), medical history (5.96%), lifestyles (5.58%), and socioeconomic status (2.37%).

**Table 3:** Weighted and unweighted PAF for the five domains.

Domains	Model1			Model2		
	Unweighted PAF	Communality	Weighted PAF	Unweighted PAF	Communality	Weighted PAF
Socioeconomic status	1.47%	20.45%	0.66%	6.30%	14.20%	2.37%
Medical history	8.15%	32.41%	4.64%	12.57%	22.44%	5.96%
Psychosocial factors	11.02%	39.50%	6.93%	15.03%	38.62%	9.34%
Physical measures	9.26%	23.39%	4.48%	18.14%	34.26%	10.62%
Lifestyles	9.27%	18.27%	3.96%	12.60%	19.59%	5.58%
Overall weighted PAF			20.67%			33.87%

PAF, population attributable fraction. In Model 1, we transformed adverse factors into moderate and favorable ones. In Model 2, we converted both adverse and moderate factors into favorable ones.

## 4 Discussion

In our research, we investigated the intricate etiology of PD, identifying modifiable risk factors across five domains: socioeconomic status, medical history, psychosocial factors, physical measures, and lifestyles. Adverse profiles in the five domains were significantly associated with increased PD risk, and significant interactions were observed between the five domains and individual’s genetic predispositions in this research. Furthermore, PD was observed to potentially mediate the impact of these exposures on blood assays. Our findings suggested that effective interventions targeting these areas could prevent 20.67 to 33.87% of PD cases in the current population.

Our study identified 45 factors significantly associated with PD, covering a wide range of domains. Consistent with previous research, well-established factors included hand grip strength (Mey et al., 2023; Liu et al., 2024), walking speed (Wu et al., 2024), physical activity (Yang et al., 2015; Baumeister et al., 2021), time spent outdoors in summer (Hu et al., 2024), smoking (Larsson and Burgess, 2022), alcohol consumption (Shao et al., 2021; Dominguez-Baleon et al., 2021), measles (Harris et al., 2012), lipoprotein metabolism disorders (Dai et al., 2021), stroke (Hommel et al., 2022), diabetes (Brauer et al., 2020; Bjornevik and Ascherio, 2023), and ischaemic heart disease (Choi et al., 2020). Moreover, psychosocial measures such as loneliness (McDaniels et al., 2023), tiredness (Mantri et al., 2020), and depressive symptoms (Usnich et al., 2023) were also significant correlates. Additionally, our research illuminated relatively unexplored factors that may be associated with PD, encompassing arm fat percentage, varicella, mumps, angina pectoris, chest pain, unemployment, difficulty getting up in the morning, and emotions such as miserableness, worry and tension. In stratified analyses, these factors did not exhibit any potential directional changes, suggesting a universal impact on PD risk, unaffected by demographic and genetic variables. The current MR analysis supported only a limited number of associated factors. One reason could be the presence of reverse causality relationships (Zhao et al., 2023). Secondly, the relationship between certain factors and PD could be complex, involving nonlinear effects. Furthermore, the strength of genetic instruments could lead to discrepancies between phenotype and MR associations (Burgess et al., 2011). The observed inconsistencies in these factors necessitate further investigation to verify.

Our study uncovered significant correlations between PD and blood assays. We found that PD acts as an intermediate factor, causing a lowered lymphocyte percentage, an elevated neutrophil percentage, and higher levels of total bilirubin. This shift in the ratio of immune cells could reflect the dynamic changes in the inflammatory response within PD patients (Rocha et al., 2018; Kim et al., 2023), aiding in the assessment of disease progression. The increase in total bilirubin levels could be related to liver dysfunction (Kwo et al., 2017), suggesting PD’s potential impact on metabolic pathways. Importantly, through MR analysis, we further confirmed the causality of these associations, enriching our understanding of the pathophysiological mechanisms of PD.

When eliminating unfavorable factors, good psychological health had the greatest potential to prevent PD, preventing 6.93% of PD cases. When further eliminating both unfavorable and moderate factors, physical measure was the leading area in reducing the incidence of PD, contributing up to 10.62%, while good psychological health was close behind, preventing 9.34% of PD cases. Improving physical measures should focus on increasing hand grip strength and reducing the arm fat percentage. Research has demonstrated that strengthening grip strength may potentially confer protective effects on the nervous system (Mey et al., 2023). Additionally, a recent study has shown an enhancement in hand grip strength is correlated with better cardiovascular health (Lawman et al., 2016). A higher arm fat percentage is related to an increased metabolic syndrome risk, and effective reduction can improve metabolic health (Shi et al., 2020; Qiu et al., 2024). Existing research has confirmed that loneliness is tied to a greater probability of developing PD, an association that remains consistent across different gender, age, and PRS (Terracciano et al., 2023). Similarly, the significant positive relationship between fatigue and PD risk has also been confirmed (Siciliano et al., 2018). Additionally, interventions improving mental health benefit cardiovascular health, potentially reducing the risk of PD (Levine et al., 2021). The novelty of our findings lies in the suggestion that both physical measures and psychological health should be prioritized in public health projects aimed at developing preventive strategies for PD. Typically, public health initiatives may focus on a single aspect, such as physical activity or mental well-being, but our results highlight the importance of addressing both domains concurrently.

Medical history also contributed PD, the PAF were 5.96%. In line with previous findings, our research indicated a positive relationship between cardiovascular diseases and PD risk (Hommel et al., 2022; Choi et al., 2020). A study has demonstrated a correlation between long-term illness or disability and an increased risk of loneliness (McGlone and Long, 2021). Our findings suggested that public health programs should prioritize not only the management of common cardiovascular and metabolic diseases, but also to address the health issues of people with long-standing illness, disabilities, and a history of falls.

A healthy lifestyle could prevent 5.58% of PD cases. Consistent with previous research, increasing daily walking speed could effectively reduce the risk of PD (Liu et al., 2024). The most recent review study further emphasized the benefits of exercise, noting that it lowers the risk of developing PD, it may also help slow the development of the condition and serves as an adjunctive therapy to control symptoms (Langeskov-Christensen et al., 2024). Increasing outdoor time in the summer can improve physical activity levels (Wang et al., 2020) and increase vitamin D levels (Samefors et al., 2020). Vitamin D exerts a beneficial influence on improving neurodegenerative changes (Wang et al., 2023). In addition, the impact of factors such as changes in diet, early rising habits, and daytime napping habits on Parkinson’s has not been completely explored and requires additional studies to clarify their mechanisms.

The PAF for socioeconomic status was relatively small (2.37%). In fact, individuals with lower socioeconomic status could be more susceptible to various adverse influences throughout their lives (Kivimäki et al., 2020). They often face limited medical resources, higher psychological stress (Cundiff et al., 2022), unbalanced diets (Brettschneider et al., 2021), and lack of physical activity, all these factors might elevate the likelihood of developing PD. Therefore, improving socioeconomic conditions and reducing health inequalities arising from socioeconomic status should also be considered an essential component of a comprehensive PD prevention strategy.

Furthermore, we did not calculate the PAF for early life factors and environmental variables because their statistical significance was less than the factors we examined. The impact of early life factors on PD might not be significant due to the extended time span. Regrettably, owing to the limitations of the UK Biobank data, our study did not incorporate environmental factors such as pesticides and insecticides, even though there is evidence suggesting that contact with these chemicals could lead to a higher chance of developing PD (Perrin et al., 2020). It is worth noting that improving environmental factors has been proven to help reduce the incidence of cardiovascular diseases (Liang et al., 2020), which might contribute to the prevention of PD.

Our study indicated that active intervention in physical measures, psychological health, lifestyles, and medical history, the incidence of PD could be significantly reduced. This underscored the importance of adopting a multifaceted intervention strategy, which encompasses not only the establishment of public health policies but also the positive modification of individual health behaviors. Although we excluded factors with high collinearity, the interrelationships between them may be more complex than mere co-occurrence. Overall, our findings suggested that up to 33.87% of PD cases could be prevented through the aforementioned measures.

This study leveraged the large-scale data from UK Biobank to investigate a wide range of modifiable risk factors, providing a multi-faceted understanding of PD etiology. By introducing composite scores, it effectively assessed the combined impact of modifiable risk factors across various domains on PD risk, and analyzed their interactions with genetic predispositions. The application of PAF analyses offered new insights and scientific support for quantifying preventive potential. However, limitations were also noted. Firstly, the UK Biobank sample selection might be biased, due to a higher participation of volunteers and the relatively younger age of the sample, which could affect the accuracy of exposure-disease relationship assessments. Secondly, some variables relied on self-reporting, which might introduce inaccuracies. Furthermore, PAF estimates are dependent on national and epidemiological data, making the results potentially not generalizable to other populations, necessitating further validation across different groups. In spite of these constraints, our findings provided new insights and scientific evidence for the formulation of preventative and therapeutic strategies for PD.

## 5 Conclusion

Our comprehensive multifactorial analysis uncovered the association of PD with a variety of factors, particularly weak grip strength, long-term illness, chest pain, and disability, as well as psychological status such as anxiety, tension, and depression. By reducing or avoiding modifiable risk factors, the incidence of PD could be reduced by 33.87%. When formulating prevention strategies, special attention should be given to areas including physical measures, psychosocial factors, lifestyle, medical history, and socioeconomic status.

## Data Availability

The original contributions presented in the study are included in the article/Supplementary material, further inquiries can be directed to the corresponding authors.

## Author’s note

All the authors take responsibility for all aspects of the reliability and freedom from bias of the data presented and their discussed interpretation.
