# Supplementary material for: Joint effect of modifiable risk factors on Parkinson’s disease: a large-scale longitudinal study
Source: Front Hum Neurosci. 2025 Jan 27;19:1525248. doi: 10.3389/fnhum.2025.1525248 (PMC11808133; doi:10.3389/fnhum.2025.1525248)

Supplementary Material

# Data preprocessing

From the available exposure variables in the UK Biobank, we first excluded those that are non-modifiable, including genomics, cognitive function, and biospecimens. We focused on data collected at baseline and excluded variables with missing values exceeding 20% of the total sample, as well as those with no practical significance. For meaningful negative values that appeared in the data, we recoded them (For example, -10 means less than 1, recode it as 0.5). For negative values that are not meaningful, we marked them as missing. For example, -1 means “Do not know”, recode it as 0.5. When addressing missing variables, we adopted different strategies. For categorical variables, we used the mode to fill in missing values; for continuous variables, we used the median for filling. For the categorical variables, we re-encoded them following a logical sequence. For multiple-choice categorical variables, we employed a dummy coding method to convert them into binary categorical variables (For example, education could be encoded as "high education" or "low education"). All exposure variables after data processing were summarized in Supplementary Table2.

In the analysis of medical history, we utilized the "Date of First Occurrence" data based on the International Classification of Diseases (ICD) coding. We marked abnormal dates as missing and excluded diseases with a prevalence rate below 2%. For some dietary data, we derived a Healthy Diet Score based on the American Heart Association guidelines ^1^, using intakes of vegetables, fruits, fish, unprocessed red meat, and processed meats. Alcohol score was calculated by converting self-reported intake to daily standard units ^2^, multiplying each alcohol source by its corresponding factor ^3^. In the physical assessment section, If the assessment includes two limbs, we took the average value. Lung function evaluation was based on the mean Z-score of FVC, FEV1, and PEF5. For air pollution-related variables with multi-year data, we computed the average over the years.

# Selection of traits for Mendelian randomization

In case where exact trait was unattainable, similar traits from the UK Biobank could be used as substitutes. For example, "Time spent doing moderate physical activity" could replace "MET minutes per week for moderate activity". For variables, such as arm fat percentage, data preprocessing combined measurements from both left and right arms. However, given that GWAS summary statistics remained independent, separate MR analyses were conducted. The GWAS ID for each trait was shown in Supplementary Table5.

# Genotype data, QC, and PRS

The specifics of genotyping and quality control have been previously documented in the literature ^4^. We executed a series of quality control protocols, resulting in the exclusion of SNPs with a minor allele frequency (MAF) of less than 0.05 and those with a genotyping call rate below 95%. Additionally, SNPs that failed to meet the criteria of the Hardy-Weinberg equilibrium test (p>1×10^−6^) were also removed to rule out possible confounding factors.

The calculation of PRS for PD was based on a Clustering subset of SNPs that exceeded a particular threshold for the P-value in GWAS ^5^. We obtained the GWAS statistics of PD from the IEU Open GWAS Project (<https://gwas.mrcieu.ac.uk/>). We selected the GWAS dataset of ieu-b-7 with the largest sample size and available status, which includes a total of 482730 Europeans (comprising 33674 cases and 449056 controls). We filtered out GWAS statistical results for variants with p≥5×10^−8^ and employed linkage disequilibrium to identify independently associated variants with r^2^<0.001. PRSlice-2 in the UK Biobank Research Analysis Platform was used to calculate PRS. PRS for each participant were derived by weighting each SNP according to its effect size from GWAS and then summing these weighted values.

# Baseline Characteristics of the Sample

In the cohort of 452,492 participants, 3348 developed PD during the follow-up, with an average age at diagnosis of 62.85 years (SD=5.34). PD patients demonstrated a higher proportion of males (64.1%) compared to the control group. They also exhibited a higher mean BMI (27.72 ± 4.43 vs. 27.37 ± 4.73), lower household incomes (less than 18,000: 27.2% vs. 19.1%; 18,000-30,999: 24.3% vs. 21.7%), and lower educational levels (66.2% vs. 62.3%). Additional details were shown in Supplementary Table3.

1 Lichtenstein, A. H. *et al.* 2021 Dietary Guidance to Improve Cardiovascular Health: A Scientific Statement From the American Heart Association. *Circulation* **144**, E472-E487, doi:10.1161/cir.0000000000001031 (2021).

2 Lloyd-Jones, D. M. *et al.* Defining and Setting National Goals for Cardiovascular Health Promotion and Disease Reduction The American Heart Association's Strategic Impact Goal Through 2020 and Beyond. *Circulation* **121**, 586-613, doi:10.1161/circulationaha.109.192703 (2010).

3 Kalinowski, A. & Humphreys, K. Governmental standard drink definitions and low-risk alcohol consumption guidelines in 37 countries. *Addiction* **111**, 1293-1298, doi:10.1111/add.13341 (2016).

4 Bycroft, C. *et al.* The UK Biobank resource with deep phenotyping and genomic data. *Nature* **562**, 203-+, doi:10.1038/s41586-018-0579-z (2018).

5 Choi, S. W., Mak, T. S. H. & O'Reilly, P. F. Tutorial: a guide to performing polygenic risk score analyses. *Nat. Protoc.* **15**, 2759-2772, doi:10.1038/s41596-020-0353-1 (2020).

# Supplementary Tables and Figures

## Supplementary Tables

**Supplementary Table1 Main analysis of sources of information for 3348 Parkinson's disease**

| Number of cases | Hospital admission | Death registries | Primary care | Self-report |
| --- | --- | --- | --- | --- |
| 3348 | 2803 | 57 | 324 | 164 |

**Supplementary Table2 variables and processing**

| **Lifestyles** | | | | | | | | | | | | | | | | | | | | | | | |
| --- | --- | --- | --- | --- | --- | --- | --- | --- | --- | --- | --- | --- | --- | --- | --- | --- | --- | --- | --- | --- | --- | --- | --- |
| **Field ID** | | **Description** | | **Coding** | | | | | | | | | | | | | | | | **Coding notes** | | | |
| 1289 | | Cooked vegetable intake | | 100373 | | | | | -10 | | | Less than one | | | | | | | | set as 0.5 | | | tablespoons/day |
|  |  |  |  |  |  |  |  |  | -1 | | | Do not know | | | | | | | | set as NA | | |  |
|  |  |  |  |  |  |  |  |  | -3 | | | Prefer not to answer | | | | | | | | set as NA | | |  |
| 1299 | | Salad / raw vegetable intake | | 100373 | | | | | same as above | | | | | | | | | | |  | | | tablespoons/day |
| 1309 | | Fresh fruit intake | | 100373 | | | | | same as above | | | | | | | | | | |  | | | pieces/day |
| 1319 | | Dried fruit intake | | 100373 | | | | | same as above | | | | | | | | | | |  | | | pieces/day |
| 1329 | | Oily fish intake | | 100377 | | | | | 0 | | | Never | | | | | | | | set as 0 | | |  |
|  |  |  |  |  |  |  |  |  | 1 | | | Less than once a week | | | | | | | | set as 0.5 | | |  |
|  |  |  |  |  |  |  |  |  | 2 | | | Once a week | | | | | | | | set as 1 | | |  |
|  |  |  |  |  |  |  |  |  | 3 | | | 2-4 times a week | | | | | | | | set as 3 | | |  |
|  |  |  |  |  |  |  |  |  | 4 | | | 5-6 times a week | | | | | | | | set as 5.5 | | |  |
|  |  |  |  |  |  |  |  |  | 5 | | | Once or more daily | | | | | | | | set as 7 | | |  |
|  |  |  |  |  |  |  |  |  | -1 | | | Do not know | | | | | | | | set as NA | | |  |
|  |  |  |  |  |  |  |  |  | -3 | | | Prefer not to answer | | | | | | | | set as NA | | |  |
| 1339 | | Non-oily fish intake | | 100377 | | | | | same as above | | | | | | | | | | |  | | |  |
| 1349 | | Processed meat intake | | 100377 | | | | | same as above | | | | | | | | | | |  | | |  |
| 1359 | | Poultry intake | | 100377 | | | | | same as above | | | | | | | | | | |  | | |  |
| 1369 | | Beef intake | | 100377 | | | | | same as above | | | | | | | | | | |  | | |  |
| 1379 | | Lamb/mutton intake | | 100377 | | | | | same as above | | | | | | | | | | |  | | |  |
| 1389 | | Pork intake | | 100377 | | | | | same as above | | | | | | | | | | |  | | |  |
| 1408 | | Cheese intake | | 100377 | | | | | same as above | | | | | | | | | | |  | | |  |
| 1418 | | Milk type used | | 100387 | | | | | 1 | | | Full cream | | | | | | | | set as 1 | | | 1: Have milk,  0:Never/rarely have milk |
|  |  |  |  |  |  |  |  |  | 2 | | | Semi-skimmed | | | | | | | | set as 1 | | |  |
|  |  |  |  |  |  |  |  |  | 3 | | | Skimmed | | | | | | | | set as 1 | | |  |
|  |  |  |  |  |  |  |  |  | 4 | | | Soya | | | | | | | | set as 1 | | |  |
|  |  |  |  |  |  |  |  |  | 5 | | | Other type of milk | | | | | | | | set as 1 | | |  |
|  |  |  |  |  |  |  |  |  | 6 | | | Never/rarely have milk | | | | | | | | set as 0 | | |  |
|  |  |  |  |  |  |  |  |  | -1 | | | Do not know | | | | | | | | set as NA | | |  |
|  |  |  |  |  |  |  |  |  | -3 | | | Prefer not to answer | | | | | | | | set as NA | | |  |
| 1428 | | Spread type | | 100388 | | | | | 0 | | | Never/rarely use spread | | | | | | | | set as 0 | | | 1: Use spread,  0:Never/rarely use spread |
|  |  |  |  |  |  |  |  |  | 1 | | | Butter/spreadable butter | | | | | | | | set as 1 | | |  |
|  |  |  |  |  |  |  |  |  | 2 | | | Flora Pro-Active /Benecol | | | | | | | | set as 1 | | |  |
|  |  |  |  |  |  |  |  |  | 3 | | | Other type of spread /margarine | | | | | | | | set as 1 | | |  |
|  |  |  |  |  |  |  |  |  | -1 | | | Do not know | | | | | | | | set as NA | | |  |
|  |  |  |  |  |  |  |  |  | -3 | | | Prefer not to answer | | | | | | | | set as NA | | |  |
| 1438 | | Bread intake | | 100373 | | | | | same as above | | | | | | | | | | |  | | | slices/week |
| 1448 | | Bread type | | 100391 | | | | | 1 | | | White | | | | | | | |  | | |  |
|  |  |  |  |  |  |  |  |  | 2 | | | Brown | | | | | | | |  | | |  |
|  |  |  |  |  |  |  |  |  | 3 | | | Wholemeal or wholegrain | | | | | | | |  | | |  |
|  |  |  |  |  |  |  |  |  | 4 | | | Other type of bread | | | | | | | |  | | |  |
|  |  |  |  |  |  |  |  |  | -1 | | | Do not know | | | | | | | | set as NA | | |  |
|  |  |  |  |  |  |  |  |  | -3 | | | Prefer not to answer | | | | | | | | set as NA | | |  |
| 1458 | | Cereal intake | | 100373 | | | | | same as above | | | | | | | | | | |  | | | bowls/week |
| 1468 | | Cereal type | | 100393 | | | | | 1 | | | Bran cereal (e.g. All Bran, Branflakes) | | | | | | | |  | | |  |
|  |  |  |  |  |  |  |  |  | 2 | | | Biscuit cereal (e.g. Weetabix) | | | | | | | |  | | |  |
|  |  |  |  |  |  |  |  |  | 3 | | | Oat cereal (e.g. Ready Brek, porridge) | | | | | | | |  | | |  |
|  |  |  |  |  |  |  |  |  | 4 | | | Muesli | | | | | | | |  | | |  |
|  |  |  |  |  |  |  |  |  | 5 | | | Other (e.g. Cornflakes, Frosties) | | | | | | | |  | | |  |
|  |  |  |  |  |  |  |  |  | -1 | | | Do not know | | | | | | | | set as NA | | |  |
|  |  |  |  |  |  |  |  |  | -3 | | | Prefer not to answer | | | | | | | | set as NA | | |  |
| 1478 | | Salt added to food | | 100394 | | | | | 1 | | | Never/rarely | | | | | | | |  | | |  |
|  |  |  |  |  |  |  |  |  | 2 | | | Sometimes | | | | | | | |  | | |  |
|  |  |  |  |  |  |  |  |  | 3 | | | Usually | | | | | | | |  | | |  |
|  |  |  |  |  |  |  |  |  | 4 | | | Always | | | | | | | |  | | |  |
|  |  |  |  |  |  |  |  |  | -3 | | | Prefer not to answer | | | | | | | | set as NA | | |  |
| 1488 | | Tea intake | | 100373 | | | | | same as above | | | | | | | | | | |  | | |  |
| 1498 | | Coffee intake | | 100373 | | | | | same as above | | | | | | | | | | |  | | |  |
| 1508 | | Coffee type | | 100397 | | | | | 1 | | | Decaffeinated coffee (any type) | | | | | | | |  | | |  |
|  |  |  |  |  |  |  |  |  | 2 | | | Instant coffee | | | | | | | |  | | |  |
|  |  |  |  |  |  |  |  |  | 3 | | | Ground coffee (include espresso, filter etc) | | | | | | | |  | | |  |
|  |  |  |  |  |  |  |  |  | 4 | | | Other type of coffee | | | | | | | |  | | |  |
|  |  |  |  |  |  |  |  |  | -1 | | | Do not know | | | | | | | | set as NA | | |  |
|  |  |  |  |  |  |  |  |  | -3 | | | Prefer not to answer | | | | | | | | set as NA | | |  |
| 1518 | | Hot drink temperature | | 100398 | | | | | 1 | | | Very hot | | | | | | | |  | | |  |
|  |  |  |  |  |  |  |  |  | 2 | | | Hot | | | | | | | |  | | |  |
|  |  |  |  |  |  |  |  |  | 3 | | | Warm | | | | | | | |  | | |  |
|  |  |  |  |  |  |  |  |  | -2 | | | Do not drink hot drinks | | | | | | | | set as 0 | | |  |
|  |  |  |  |  |  |  |  |  | -3 | | | Prefer not to answer | | | | | | | | set as NA | | |  |
| 1528 | | Water intake | | 100373 | | | | | same as above | | | | | | | | | | |  | | |  |
| 1538 | | Major dietary changes in the last 5 years | | 100400 | | | | | 0 | | | No | | | | | | | |  | | |  |
|  |  |  |  |  |  |  |  |  | 1 | | | Yes, because of illness | | | | | | | |  | | |  |
|  |  |  |  |  |  |  |  |  | 2 | | | Yes, because of other reasons | | | | | | | |  | | |  |
|  |  |  |  |  |  |  |  |  | -3 | | | Prefer not to answer | | | | | | | | set as NA | | |  |
| 1120 | | Weekly usage of mobile phone in last 3 months | | 100336 | | | | | 0 | | | Less than 5mins | | | | | | | |  | | |  |
|  |  |  |  |  |  |  |  |  | 1 | | | 5-29 mins | | | | | | | |  | | |  |
|  |  |  |  |  |  |  |  |  | 2 | | | 30-59 mins | | | | | | | |  | | |  |
|  |  |  |  |  |  |  |  |  | 3 | | | 1-3 hours | | | | | | | |  | | |  |
|  |  |  |  |  |  |  |  |  | 4 | | | 4-6 hours | | | | | | | |  | | |  |
|  |  |  |  |  |  |  |  |  | 5 | | | More than 6 hours | | | | | | | |  | | |  |
|  |  |  |  |  |  |  |  |  | -1 | | | Do not know | | | | | | | | set as NA | | |  |
|  |  |  |  |  |  |  |  |  | -3 | | | Prefer not to answer | | | | | | | | set as NA | | |  |
| 1130 | | Hands-free device/speakerphone use with mobile phone in last 3 month | | 100337 | | | | | 0 | | | Never or almost never | | | | | | | |  | | |  |
|  |  |  |  |  |  |  |  |  | 1 | | | Less than half the time | | | | | | | |  | | |  |
|  |  |  |  |  |  |  |  |  | 2 | | | About half the time | | | | | | | |  | | |  |
|  |  |  |  |  |  |  |  |  | 3 | | | More than half the time | | | | | | | |  | | |  |
|  |  |  |  |  |  |  |  |  | 4 | | | Always or almost always | | | | | | | |  | | |  |
|  |  |  |  |  |  |  |  |  | -1 | | | Do not know | | | | | | | | set as NA | | |  |
|  |  |  |  |  |  |  |  |  | -3 | | | Prefer not to answer | | | | | | | | set as NA | | |  |
| 1150 | | Usual side of head for mobile phone use | | 100339 | | | | | 1 | | | Left | | | | | | | |  | | |  |
|  |  |  |  |  |  |  |  |  | 2 | | | Right | | | | | | | |  | | |  |
|  |  |  |  |  |  |  |  |  | 3 | | | Equally left and right | | | | | | | | set as 0 | | |  |
|  |  |  |  |  |  |  |  |  | -1 | | | Do not know | | | | | | | | set as NA | | |  |
|  |  |  |  |  |  |  |  |  | -3 | | | Prefer not to answer | | | | | | | | set as NA | | |  |
| 2237 | | Plays computer games | | 100639 | | | | | 0 | | | Never/rarely | | | | | | | |  | | |  |
|  |  |  |  |  |  |  |  |  | 1 | | | Sometimes | | | | | | | |  | | |  |
|  |  |  |  |  |  |  |  |  | 2 | | | Often | | | | | | | |  | | |  |
|  |  |  |  |  |  |  |  |  | -3 | | | Prefer not to answer | | | | | | | | set as NA | | |  |
| 1160 | | Sleep duration | | 100291 | | | | | -1 | | | Do not know | | | | | | | | set as NA | | | hours/day |
|  |  |  |  |  |  |  |  |  | -3 | | | Prefer not to answer | | | | | | | | set as NA | | |  |
| 1170 | | Getting up in morning | | 100341 | | | | | 1 | | | Not at all easy | | | | | | | |  | | |  |
|  |  |  |  |  |  |  |  |  | 2 | | | Not very easy | | | | | | | |  | | |  |
|  |  |  |  |  |  |  |  |  | 3 | | | Fairly easy | | | | | | | |  | | |  |
|  |  |  |  |  |  |  |  |  | 4 | | | Very easy | | | | | | | |  | | |  |
|  |  |  |  |  |  |  |  |  | -1 | | | Do not know | | | | | | | | set as NA | | |  |
|  |  |  |  |  |  |  |  |  | -3 | | | Prefer not to answer | | | | | | | | set as NA | | |  |
| 1190 | | Nap during day | | 100343 | | | | | 1 | | | Never/rarely | | | | | | | |  | | |  |
|  |  |  |  |  |  |  |  |  | 2 | | | Sometimes | | | | | | | |  | | |  |
|  |  |  |  |  |  |  |  |  | 3 | | | Usually | | | | | | | |  | | |  |
|  |  |  |  |  |  |  |  |  | -3 | | | Prefer not to answer | | | | | | | | set as NA | | |  |
| 1200 | | Sleeplessness / insomnia | | 100343 | | | | | same as above | | | | | | | | | | |  | | |  |
| 1210 | | Snoring | | 100345 | | | | | 1 | | | Yes | | | | | | | |  | | |  |
|  |  |  |  |  |  |  |  |  | 2 | | | No | | | | | | | |  | | |  |
|  |  |  |  |  |  |  |  |  | -1 | | | Do not know | | | | | | | | set as NA | | |  |
|  |  |  |  |  |  |  |  |  | -3 | | | Prefer not to answer | | | | | | | | set as NA | | |  |
| 1220 | | Daytime dozing / sleeping (narcolepsy) | | 100346 | | | | | 0 | | | Never/rarely | | | | | | | |  | | |  |
|  |  |  |  |  |  |  |  |  | 1 | | | Sometimes | | | | | | | |  | | |  |
|  |  |  |  |  |  |  |  |  | 2 | | | Often | | | | | | | |  | | |  |
|  |  |  |  |  |  |  |  |  | -1 | | | Do not know | | | | | | | | set as NA | | |  |
|  |  |  |  |  |  |  |  |  | -3 | | | Prefer not to answer | | | | | | | | set as NA | | |  |
|  |  |  |  |  |  |  |  |  | 3 | | | All of the time | | | | | | | |  | | |  |
| 1050 | | Time spend outdoors in summer | | 100329 | | | | | -10 | | | Less than an hour a day | | | | | | | | set as 0.5 | | |  |
|  |  |  |  |  |  |  |  |  | -1 | | | Do not know | | | | | | | | set as NA | | |  |
|  |  |  |  |  |  |  |  |  | -3 | | | Prefer not to answer | | | | | | | | set as NA | | |  |
| 1060 | | Time spent outdoors in winter | | 100329 | | | | | same as above | | | | | | | | | | |  | | |  |
| 2267 | | Use of sun/uv protection | | 100536 | | | | | 1 | | | Never/rarely | | | | | | | |  | | |  |
|  |  |  |  |  |  |  |  |  | 2 | | | Sometimes | | | | | | | |  | | |  |
|  |  |  |  |  |  |  |  |  | 3 | | | Most of the time | | | | | | | |  | | |  |
|  |  |  |  |  |  |  |  |  | 4 | | | Always | | | | | | | |  | | |  |
|  |  |  |  |  |  |  |  |  | 5 | | | Do not go out in sun-shine | | | | | | | | set as 0 | | |  |
|  |  |  |  |  |  |  |  |  | -1 | | | Do not know | | | | | | | | set as NA | | |  |
|  |  |  |  |  |  |  |  |  | -3 | | | Prefer not to answer | | | | | | | | set as NA | | |  |
| 2277 | | Frequency of solarium/ sunlamp use | | 100537 | | | | | -10 | | | Less than once a year | | | | | | | | set as 0.5 | | |  |
|  |  |  |  |  |  |  |  |  | -1 | | | Do not know | | | | | | | | set as NA | | |  |
|  |  |  |  |  |  |  |  |  | -3 | | | Prefer not to answer | | | | | | | | set as NA | | |  |
| 1070 | | Time spent watching television (TV) | | 100329 | | | | | same as above | | | | | | | | | | |  | | |  |
| 1080 | | Time spent using computer | | 100329 | | | | | same as above | | | | | | | | | | |  | | |  |
| 1090 | | Time spent driving | | 100329 | | | | | same as above | | | | | | | | | | |  | | |  |
| 874 | | Duration of walks | | 100291 | | | | | same as above | | | | | | | | | | |  | | |  |
| 943 | | Frequency of stair climbing in last 4 weeks | | 100314 | | | | | 0 | | | None | | | | | | | | set as 0 | | | 0: Less than 10 times a day  1: More than 10 times a day |
|  |  |  |  |  |  |  |  |  | 1 | | | 1-5 times a day | | | | | | | | set as 0 | | |  |
|  |  |  |  |  |  |  |  |  | 2 | | | 6-10 times a day | | | | | | | | set as 0 | | |  |
|  |  |  |  |  |  |  |  |  | 3 | | | 11-15 times a day | | | | | | | | set as 1 | | |  |
|  |  |  |  |  |  |  |  |  | 4 | | | 16-20 times a day | | | | | | | | set as 1 | | |  |
|  |  |  |  |  |  |  |  |  | 5 | | | More than 20 times a day | | | | | | | | set as 1 | | |  |
|  |  |  |  |  |  |  |  |  | -1 | | | Do not know | | | | | | | | set as NA | | |  |
|  |  |  |  |  |  |  |  |  | -3 | | | Prefer not to answer | | | | | | | | set as NA | | |  |
| 884 | | Number of days/week of moderate physical activity 10+ minutes | | 100291 | | | | | same as above | | | | | | | | | | |  | | |  |
| 904 | | Number of days/week of vigorous physical activity 10+ minutes | | 100291 | | | | | same as above | | | | | | | | | | |  | | |  |
| 864 | | Number of days/week walked 10+ minutes | | 100307 | | | | | -1 | | | Do not know | | | | | | | | set as NA | | |  |
|  |  |  |  |  |  |  |  |  | -2 | | | Unable to walk | | | | | | | | set as 0 | | |  |
|  |  |  |  |  |  |  |  |  | -3 | | | Prefer not to answer | | | | | | | | set as NA | | |  |
| 6160 | | Leisure/social activities | | 100328 | | | | | 1 | | | Sports club or gym | | | | | | | | set as 1 | | | 0: No leisure/ social activities  1: Have leisure/ social activities |
|  |  |  |  |  |  |  |  |  | 2 | | | Pub or social club | | | | | | | | set as 1 | | |  |
|  |  |  |  |  |  |  |  |  | 3 | | | Religious group | | | | | | | | set as 1 | | |  |
|  |  |  |  |  |  |  |  |  | 4 | | | Adult education class | | | | | | | | set as 1 | | |  |
|  |  |  |  |  |  |  |  |  | 5 | | | Other group activity | | | | | | | | set as 1 | | |  |
|  |  |  |  |  |  |  |  |  | -7 | | | None of the above | | | | | | | | set as 0 | | |  |
|  |  |  |  |  |  |  |  |  | -3 | | | Prefer not to answer | | | | | | | | set as NA | | |  |
| 924 | | Usual walking pace | | 100313 | | | | | 1 | | | Slow pace | | | | | | | |  | | |  |
|  |  |  |  |  |  |  |  |  | 2 | | | Steady average pace | | | | | | | | set as 0 | | |  |
|  |  |  |  |  |  |  |  |  | 3 | | | Brisk pace | | | | | | | |  | | |  |
|  |  |  |  |  |  |  |  |  | -7 | | | None of the above | | | | | | | | set as NA | | |  |
|  |  |  |  |  |  |  |  |  | -3 | | | Prefer not to answer | | | | | | | | set as NA | | |  |
| 22036 | | At or above moderate/ vigorous/walking recommendation | | 7 | | | | | 1 | | | Yes | | | | | | | |  | | |  |
|  |  |  |  |  |  |  |  |  | 0 | | | No | | | | | | | |  | | |  |
| 22032 | | IPAQ activity group | | 100700 | | | | | 0 | | | low | | | | | | | | set as 3 | | |  |
|  |  |  |  |  |  |  |  |  | 1 | | | moderate | | | | | | | |  | | |  |
|  |  |  |  |  |  |  |  |  | 2 | | | high | | | | | | | |  | | |  |
| 22038 | | MET minutes per week for moderate activity | |  | | | | |  | | |  | | | | | | | |  | | |  |
| 22039 | | MET minutes per week for vigorous activity | |  | | | | |  | | |  | | | | | | | |  | | |  |
| 22037 | | MET minutes per week for walking | |  | | | | |  | | |  | | | | | | | |  | | |  |
| 22040 | | Summed MET minutes per week for all activity | |  | | | | |  | | |  | | | | | | | |  | | |  |
| 22033 | | Summed days activity | |  | | | | |  | | |  | | | | | | | |  | | |  |
| 22034 | | Summed minutes activity | |  | | | | |  | | |  | | | | | | | |  | | |  |
| 6155 | | Vitamin and mineral supplements | | 100629 | | | | | 1 | | | Vitamin A | | | | | | | | set as 1 | | | 0: No vitamin and mineral supplements  1: Have vitamin and mineral supplements |
|  |  |  |  |  |  |  |  |  | 2 | | | Vitamin B | | | | | | | | set as 1 | | |  |
|  |  |  |  |  |  |  |  |  | 3 | | | Vitamin C | | | | | | | | set as 1 | | |  |
|  |  |  |  |  |  |  |  |  | 4 | | | Vitamin D | | | | | | | | set as 1 | | |  |
|  |  |  |  |  |  |  |  |  | 5 | | | Vitamin E | | | | | | | | set as 1 | | |  |
|  |  |  |  |  |  |  |  |  | 6 | | | Folic acid or Folate (Vit B9) | | | | | | | | set as 1 | | |  |
|  |  |  |  |  |  |  |  |  | 7 | | | Multivitamins +/- minerals | | | | | | | | set as 1 | | |  |
|  |  |  |  |  |  |  |  |  | -7 | | | None of the above | | | | | | | | set as 0 | | |  |
|  |  |  |  |  |  |  |  |  | -3 | | | Prefer not to answer | | | | | | | | set as NA | | |  |
| 6179 | | Mineral and other dietary supplements | | 100630 | | | | | 1 | | | Fish oil (including cod liver oil) | | | | | | | | set as 1 | | | 0: No mineral and other dietary supplements  1: Have mineral and other dietary supplements |
|  |  |  |  |  |  |  |  |  | 2 | | | Glucosamine | | | | | | | | set as 1 | | |  |
|  |  |  |  |  |  |  |  |  | 3 | | | Calcium | | | | | | | | set as 1 | | |  |
|  |  |  |  |  |  |  |  |  | 4 | | | Zinc | | | | | | | | set as 1 | | |  |
|  |  |  |  |  |  |  |  |  | 5 | | | Iron | | | | | | | | set as 1 | | |  |
|  |  |  |  |  |  |  |  |  | 6 | | | Selenium | | | | | | | | set as 1 | | |  |
|  |  |  |  |  |  |  |  |  | -7 | | | None of the above | | | | | | | | set as 0 | | |  |
|  |  |  |  |  |  |  |  |  | -3 | | | Prefer not to answer | | | | | | | | set as NA | | |  |
| 2139 | | Age first had sexual intercourse | | 100504 | | | | | -2 | | | Never had sex | | | | | | | |  | | |  |
|  |  |  |  |  |  |  |  |  | -3 | | | Prefer not to answer | | | | | | | | set as NA | | |  |
|  |  |  |  |  |  |  |  |  | -1 | | | Do not know | | | | | | | | set as NA | | |  |
| 2149 | | Lifetime number of sexual partners | | 100291 | | | | | same as above | | | | | | | | | | |  | | |  |
| 2159 | | Ever had same-sex intercourse | | 100352 | | | | | 1 | | | Yes | | | | | | | |  | | |  |
|  |  |  |  |  |  |  |  |  | 0 | | | No | | | | | | | |  | | |  |
|  |  |  |  |  |  |  |  |  | -3 | | | Prefer not to answer | | | | | | | | set as NA | | |  |
| 1568 | | Average weekly red wine intake | | 100291 | | | | | same as above | | | | | | | | | | |  | | | glasses |
| 4407 | | Average monthly red wine intake | | 100291 | | | | | same as above | | | | | | | | | | |  | | | glasses |
| 1578 | | Average weekly champagne plus white wine intake | | 100291 | | | | | same as above | | | | | | | | | | |  | | | glasses |
| 4418 | | Average monthly champagne plus white wine intake | | 100291 | | | | | same as above | | | | | | | | | | |  | | | glasses |
| 1588 | | Average weekly beer plus cider intake | | 100291 | | | | | same as above | | | | | | | | | | |  | | | pints |
| 4429 | | Average monthly beer plus cider intake | | 100291 | | | | | same as above | | | | | | | | | | |  | | | pints |
| 1598 | | Average weekly spirits intake | | 100291 | | | | | same as above | | | | | | | | | | |  | | | measures |
| 4440 | | Average monthly spirits intake | | 100291 | | | | | same as above | | | | | | | | | | |  | | | measures |
| 1608 | | Average weekly fortified wine intake | | 100291 | | | | | same as above | | | | | | | | | | |  | | | glasses |
| 4451 | | Average monthly fortified wine intake | | 100291 | | | | | same as above | | | | | | | | | | |  | | | glasses |
| 5364 | | Average weekly intake of other alcoholic drinks | | 100291 | | | | | same as above | | | | | | | | | | |  | | | glasses |
| 4462 | | Average monthly intake of other alcoholic drinks | | 100291 | | | | | same as above | | | | | | | | | | |  | | | glasses |
| 20117 | | Alcohol drinker status | | 90 | | | | | -3 | | | Prefer not to answer | | | | | | | | set as NA | | |  |
|  |  |  |  |  |  |  |  |  | 0 | | | Never | | | | | | | |  | | |  |
|  |  |  |  |  |  |  |  |  | 1 | | | Previous | | | | | | | |  | | |  |
|  |  |  |  |  |  |  |  |  | 2 | | | Current | | | | | | | |  | | |  |
| 1269 | | Exposure to tobacco smoke at home | | 100291 | | | | | same as above | | | | | | | | | | |  | | |  |
| 1279 | | Exposure to tobacco smoke outside home | | 100291 | | | | | same as above | | | | | | | | | | |  | | |  |
| 20116 | | Smoking status | | 90 | | | | | same as above | | | | | | | | | | |  | | |  |
| **Early life** | | | | | | | | | | | | | | | | | | | | | | | |
| **Field ID** | **Description** | | | | **Coding** | | | | | | | | | | | | | | | | **Coding notes** | | |
| 1677 | Breastfed as a baby | | | | 100349 | | | | | 1 | | | | Yes | | | | | | |  | | |
|  |  |  |  |  |  |  |  |  |  | 0 | | | | No | | | | | | |  | | |
|  |  |  |  |  |  |  |  |  |  | -1 | | | | Do not know | | | | | | | set as NA | | |
|  |  |  |  |  |  |  |  |  |  | -3 | | | | Prefer not to answer | | | | | | | set as NA | | |
| 1687 | Comparative body size at age 10 | | | | 100428 | | | | | 1 | | | | Thinner | | | | | | |  | | |
|  |  |  |  |  |  |  |  |  |  | 2 | | | | Plumper | | | | | | |  | | |
|  |  |  |  |  |  |  |  |  |  | 3 | | | | About average | | | | | | | set as 0 | | |
|  |  |  |  |  |  |  |  |  |  | -1 | | | | Do not know | | | | | | | set as NA | | |
|  |  |  |  |  |  |  |  |  |  | -3 | | | | Prefer not to answer | | | | | | | set as NA | | |
| 1697 | Comparative height size at age 10 | | | | 100429 | | | | | 1 | | | | Shorter | | | | | | |  | | |
|  |  |  |  |  |  |  |  |  |  | 2 | | | | Taller | | | | | | |  | | |
|  |  |  |  |  |  |  |  |  |  | 3 | | | | About average | | | | | | | set as 0 | | |
|  |  |  |  |  |  |  |  |  |  | -1 | | | | Do not know | | | | | | | set as NA | | |
|  |  |  |  |  |  |  |  |  |  | -3 | | | | Prefer not to answer | | | | | | | set as NA | | |
| 1707 | Handedness (chirality/laterality) | | | | 100430 | | | | | 1 | | | | Right-handed | | | | | | |  | | |
|  |  |  |  |  |  |  |  |  |  | 2 | | | | Left-handed | | | | | | |  | | |
|  |  |  |  |  |  |  |  |  |  | 3 | | | | Use both right and left hands equally | | | | | | | set as 0 | | |
|  |  |  |  |  |  |  |  |  |  | -3 | | | | Prefer not to answer | | | | | | | set as NA | | |
| 1767 | Adopted as a child | | | | 100349 | | | | | same as above | | | | | | | | | | |  | | |
| 1777 | Part of a multiple birth | | | | 100349 | | | | | same as above | | | | | | | | | | |  | | |
| 1787 | Maternal smoking around birth | | | | 100349 | | | | | same as above | | | | | | | | | | |  | | |
| **Psychosocial factors** | | | | | | | | | | | | | | | | | | | | | | | |
| **Field ID** | **Description** | | | **Coding** | | | | | | | | | | | | **Coding notes** | | | | | | | |
| 1031 | Frequency of friend/family visits | | | 100327 | | | | | 1 | | Almost daily | | | | | set as 0 | | | | | | Turn data into binary classification：1：Seldom visit friends/family; 0: Always visit friends/family | |
|  |  |  |  |  |  |  |  |  | 2 | | 2-4 times a week | | | | | set as 0 | | | | | |  |  |
|  |  |  |  |  |  |  |  |  | 3 | | About once a week | | | | | set as 0 | | | | | |  |  |
|  |  |  |  |  |  |  |  |  | 4 | | About once a month | | | | | set as 1 | | | | | |  |  |
|  |  |  |  |  |  |  |  |  | 5 | | Once every few months | | | | | set as 1 | | | | | |  |  |
|  |  |  |  |  |  |  |  |  | 6 | | Never or almost never | | | | | set as 1 | | | | | |  |  |
|  |  |  |  |  |  |  |  |  | 7 | | No friends/family outside household | | | | | set as 1 | | | | | |  |  |
|  |  |  |  |  |  |  |  |  | -1 | | Do not know | | | | | set as NA | | | | | |  |  |
|  |  |  |  |  |  |  |  |  | -3 | | Prefer not to answer | | | | | set as NA | | | | | |  |  |
| 1920 | Mood swings | | | 100349 | | | | | 1 | | Yes | | | | |  | | | | | |  | |
|  |  |  |  |  |  |  |  |  | 0 | | No | | | | |  | | | | | |  | |
|  |  |  |  |  |  |  |  |  | -1 | | Do not know | | | | | set as NA | | | | | |  | |
|  |  |  |  |  |  |  |  |  | -3 | | Prefer not to answer | | | | | set as NA | | | | | |  | |
| 1930 | Miserableness | | | 100349 | | | | | same as above | | | | | | | | | | | | | | |
| 1940 | Irritability | | | 100349 | | | | | same as above | | | | | | | | | | | | | | |
| 1950 | Sensitivity / hurt feelings | | | 100349 | | | | | same as above | | | | | | | | | | | | | | |
| 1960 | Fed-up feelings | | | 100349 | | | | | same as above | | | | | | | | | | | | | | |
| 1970 | Nervous feelings | | | 100349 | | | | | same as above | | | | | | | | | | | | | | |
| 1980 | Worrier / anxious feelings | | | 100349 | | | | | same as above | | | | | | | | | | | | | | |
| 1990 | Tense / 'highly strung' | | | 100349 | | | | | same as above | | | | | | | | | | | | | | |
| 2000 | Worry too long after embarrassment | | | 100349 | | | | | same as above | | | | | | | | | | | | | | |
| 2010 | Suffer from 'nerves' | | | 100349 | | | | | same as above | | | | | | | | | | | | | | |
| 2020 | Loneliness, isolation | | | 100349 | | | | | same as above | | | | | | | | | | | | | | |
| 2030 | Guilty feelings | | | 100349 | | | | | same as above | | | | | | | | | | | | | | |
| 2040 | Risk taking | | | 100349 | | | | | same as above | | | | | | | | | | | | | | |
| 2050 | Frequency of depressed mood in last 2 weeks | | | 100484 | | | | | 1 | | Not at all | | | | |  | | | | | |  | |
|  |  |  |  |  |  |  |  |  | 2 | | Several days | | | | |  | | | | | |  | |
|  |  |  |  |  |  |  |  |  | 3 | | More than half the days | | | | |  | | | | | |  | |
|  |  |  |  |  |  |  |  |  | 4 | | Nearly every day | | | | |  | | | | | |  | |
|  |  |  |  |  |  |  |  |  | -1 | | Do not know | | | | | set as NA | | | | | |  | |
|  |  |  |  |  |  |  |  |  | -3 | | Prefer not to answer | | | | | set as NA | | | | | |  | |
| 2060 | Frequency of unenthusiasm / disinterest in last 2 weeks | | | 100484 | | | | | same as above | | | | | | | | | | | | | | |
| 2070 | Frequency of tenseness / restlessness in last 2 weeks | | | 100484 | | | | | same as above | | | | | | | | | | | | | | |
| 2080 | Frequency of tiredness / lethargy in last 2 weeks | | | 100484 | | | | | same as above | | | | | | | | | | | | | | |
| 2090 | Seen doctor (GP) for nerves, anxiety, tension or depression | | | 100349 | | | | | same as above | | | | | | | | | | | | | | |
| 2100 | Seen a psychiatrist for nerves, anxiety, tension or depression | | | 100349 | | | | | same as above | | | | | | | | | | | | | | |
| 2110 | Able to confide | | | 100501 | | | | | 5 | | Almost daily | | | | | set as 0 | | | | | | 0: Frequently able to confide  1: Rarely able to confide | |
|  |  |  |  |  |  |  |  |  | 4 | | 2-4 times a week | | | | | set as 0 | | | | | |  |  |
|  |  |  |  |  |  |  |  |  | 3 | | About once a week | | | | | set as 0 | | | | | |  |  |
|  |  |  |  |  |  |  |  |  | 2 | | About once a month | | | | | set as 1 | | | | | |  |  |
|  |  |  |  |  |  |  |  |  | 1 | | Once every few months | | | | | set as 1 | | | | | |  |  |
|  |  |  |  |  |  |  |  |  | 0 | | Never or almost never | | | | | set as 1 | | | | | |  |  |
|  |  |  |  |  |  |  |  |  | -1 | | Do not know | | | | | set as NA | | | | | |  |  |
|  |  |  |  |  |  |  |  |  | -3 | | Prefer not to answer | | | | | set as NA | | | | | |  |  |
| **Socioeconomic status** | | | | | | | | | | | | | | | | | | | | | | | |
| **Field ID** | **Description** | | **Coding** | | | | | | | | | | | | | | | **Coding notes** | | | | | |
| 738 | Average total household income before tax | | 100294 | | 1 | | Less than 18,000 | | | | | | | | | | |  | | | | |  |
|  |  |  |  |  | 2 | | 18,000 to 30,999 | | | | | | | | | | |  | | | | |  |
|  |  |  |  |  | 3 | | 31,000 to 51,999 | | | | | | | | | | |  | | | | |  |
|  |  |  |  |  | 4 | | 52,000 to 100,000 | | | | | | | | | | |  | | | | |  |
|  |  |  |  |  | 5 | | Greater than 100,000 | | | | | | | | | | |  | | | | |  |
|  |  |  |  |  | -1 | | Do not know | | | | | | | | | | | set as NA | | | | |  |
|  |  |  |  |  | -3 | | Prefer not to answer | | | | | | | | | | | set as NA | | | | |  |
| 6142 | Current employment status | | 100295 | | 1 | | In paid employment or self-employed | | | | | | | | | | | set as 0 | | | | | 1：unemployed;  0: employed |
|  |  |  |  |  | 2 | | Retired | | | | | | | | | | | set as 0 | | | | |  |
|  |  |  |  |  | 3 | | Looking after home and/or family | | | | | | | | | | | set as 1 | | | | |  |
|  |  |  |  |  | 4 | | Unable to work because of sickness or disability | | | | | | | | | | | set as 1 | | | | |  |
|  |  |  |  |  | 5 | | Unemployed | | | | | | | | | | | set as 1 | | | | |  |
|  |  |  |  |  | 6 | | Doing unpaid or voluntary work | | | | | | | | | | | set as 0 | | | | |  |
|  |  |  |  |  | 7 | | Full or part-time student | | | | | | | | | | | set as 0 | | | | |  |
|  |  |  |  |  | -7 | | None of the above | | | | | | | | | | | set as NA | | | | |  |
|  |  |  |  |  | -3 | | Prefer not to answer | | | | | | | | | | | set as NA | | | | |  |
| 6138 | Qualifications | | 100305 | | 1 | | College or University degree | | | | | | | | | | | set as 0 | | | | | 1: lower education;  0: higher education |
|  |  |  |  |  | 2 | | A levels/AS levels or equivalent | | | | | | | | | | | set as 1 | | | | |  |
|  |  |  |  |  | 3 | | O levels/GCSEs or equivalent | | | | | | | | | | | set as 1 | | | | |  |
|  |  |  |  |  | 4 | | CSEs or equivalent | | | | | | | | | | | set as 1 | | | | |  |
|  |  |  |  |  | 5 | | NVQ or HND or HNC or equivalent | | | | | | | | | | | set as 1 | | | | |  |
|  |  |  |  |  | 6 | | Other professional qualifications eg: nursing, teaching | | | | | | | | | | | set as 0 | | | | |  |
|  |  |  |  |  | -7 | | None of the above | | | | | | | | | | | set as NA | | | | |  |
|  |  |  |  |  | -3 | | Prefer not to answer | | | | | | | | | | | set as NA | | | | |  |
| 22189 | Townsend deprivation index at recruitment | | | | | | | | | | | | | | | | |  | | | | |  |
| **Physical measures** | | | | | | | | | | | | | | | | | | | | | | | |
| **Field ID** | **Description** | | | | | | | **Coding notes** | | | | | | | | | | | | | | | |
| 23099 | Body fat percentage | | | | | | |  | | | | | | | | |  | | | | | | |
| 23105 | Basal metabolic rate | | | | | | |  | | | | | | | | |  | | | | | | |
| 23111 | Leg fat percentage (right) | | | | | | | Mean values of left and right | | | | | | | | | Leg fat percentage | | | | | | |
| 23115 | Leg fat percentage (left) | | | | | | |  |  |  |  |  |  |  |  |  |  |  |  |  |  |  |  |
| 23119 | Arm fat percentage (right) | | | | | | | Mean values of left and right | | | | | | | | | Arm fat percentage | | | | | | |
| 23123 | Arm fat percentage (left) | | | | | | |  |  |  |  |  |  |  |  |  |  |  |  |  |  |  |  |
| 23127 | Trunk fat percentage | | | | | | |  | | | | | | | | |  | | | | | | |
| 3062 | Forced vital capacity (FVC) | | | | | | | Mean values of z-scores of 3 fields | | | | | | | | | Lung function | | | | | | |
| 3063 | Forced expiratory volume in 1-second (FEV1) | | | | | | |  |  |  |  |  |  |  |  |  |  |  |  |  |  |  |  |
| 3064 | Peak expiratory flow (PEF) | | | | | | |  |  |  |  |  |  |  |  |  |  |  |  |  |  |  |  |
| 94 | Diastolic blood pressure, manual reading | | | | | | | Mean values of 2 arrays | | | | | | | | | Diastolic blood pressure | | | | | | |
| 4079 | Diastolic blood pressure, automated reading | | | | | | |  |  |  |  |  |  |  |  |  |  |  |  |  |  |  |  |
| 93 | Systolic blood pressure, manual reading | | | | | | | Mean values of 2 arrays | | | | | | | | | Systolic blood pressure | | | | | | |
| 4080 | Systolic blood pressure, automated reading | | | | | | |  |  |  |  |  |  |  |  |  |  |  |  |  |  |  |  |
| 46 | Hand grip strength (left) | | | | | | | Mean values of 2 arrays | | | | | | | | | Hand grip strength | | | | | | |
| 47 | Hand grip strength (right) | | | | | | |  |  |  |  |  |  |  |  |  |  |  |  |  |  |  |  |
| 21001 | body mass index | | | | | | |  | | | | | | | | |  | | | | | | |
| 48 | Waist circumference | | | | | | |  | | | | | | | | |  | | | | | | |
| 49 | Hip circumference | | | | | | |  | | | | | | | | |  | | | | | | |
| 95 | Pulse rate (during blood-pressure measurement) | | | | | | | Mean values of 2 arrays | | | | | | | | | Pulse rate | | | | | | |
| 102 | Pulse rate, automated reading | | | | | | |  |  |  |  |  |  |  |  |  |  |  |  |  |  |  |  |
| **Local environment** | | | | | | | | | | | | | | | | | | | | | | | |
| **Field ID** | **Description** | | | | | | | **Coding** | | | | | | | | | | | **Coding notes** | | | | |
| 21100 | Water hardness (USGS classification) | | | | | | | 1180 | | | | | 0 | | 0-60 mg/L | | | | set as 0 | | | | <120 mg/L |
|  |  |  |  |  |  |  |  |  |  |  |  |  | 1 | | 60-120 mg/L | | | | set as 0 | | | |  |
|  |  |  |  |  |  |  |  |  |  |  |  |  | 2 | | 120-180 mg/L | | | | set as 1 | | | | >=120 mg/L |
|  |  |  |  |  |  |  |  |  |  |  |  |  | 3 | | >180 mg/L | | | | set as 1 | | | |  |
| 21101 | Water hardness (WHO classification) | | | | | | | 1181 | | | | | 0 | | <200 mg/L | | | |  | | | |  |
|  |  |  |  |  |  |  |  |  |  |  |  |  | 1 | | >=200 mg/L | | | |  | | | |  |
| 21103 | CaCO3 concentration | | | | | | | | | | | | | | | | | |  | | | |  |
| 21104 | Ca concentration | | | | | | | | | | | | | | | | | |  | | | |  |
| 21105 | Mg concentration | | | | | | | | | | | | | | | | | |  | | | |  |
| 24003 | Nitrogen dioxide air pollution; 2010 | | | | | | | | | | | | | | | | | | Mean values | | | | NO2 |
| 24016 | Nitrogen dioxide air pollution; 2005 | | | | | | | | | | | | | | | | | |  |  |  |  |  |
| 24017 | Nitrogen dioxide air pollution; 2006 | | | | | | | | | | | | | | | | | |  |  |  |  |  |
| 24018 | Nitrogen dioxide air pollution; 2007 | | | | | | | | | | | | | | | | | |  |  |  |  |  |
| 24004 | Nitrogen oxides air pollution; 2010 | | | | | | | | | | | | | | | | | |  | | | |  |
| 24006 | Particulate matter air pollution (pm2.5); 2010 | | | | | | | | | | | | | | | | | |  | | | |  |
| 24007 | Particulate matter air pollution (pm2.5) absorbance; 2010 | | | | | | | | | | | | | | | | | |  | | | |  |
| 24008 | Particulate matter air pollution 2.5-10um; 2010 | | | | | | | | | | | | | | | | | |  | | | |  |
| 24005 | Particulate matter air pollution (pm10); 2010 | | | | | | | | | | | | | | | | | | Mean values | | | | PM10 |
| 24019 | Particulate matter air pollution (pm10); 2007 | | | | | | | | | | | | | | | | | |  |  |  |  |  |
| 24020 | Average daytime sound level of noise pollution | | | | | | | | | | | | | | | | | |  | | | |  |
| 24021 | Average evening sound level of noise pollution | | | | | | | | | | | | | | | | | |  | | | |  |
| 24022 | Average night-time sound level of noise pollution | | | | | | | | | | | | | | | | | |  | | | |  |
| 24023 | Average 16-hour sound level of noise pollution | | | | | | | | | | | | | | | | | |  | | | |  |
| 24024 | Average 24-hour sound level of noise pollution | | | | | | | | | | | | | | | | | |  | | | |  |
| 24500 | Greenspace percentage, buffer 1000m | | | | | | | | | | | | | | | | | |  | | | |  |
| 24501 | Domestic garden percentage, buffer 1000m | | | | | | | | | | | | | | | | | |  | | | |  |
| 24502 | Water percentage, buffer 1000m | | | | | | | | | | | | | | | | | |  | | | |  |
| 24503 | Greenspace percentage, buffer 300m | | | | | | | | | | | | | | | | | |  | | | |  |
| 24504 | Domestic garden percentage, buffer 300m | | | | | | | | | | | | | | | | | |  | | | |  |
| 24505 | Water percentage, buffer 300m | | | | | | | | | | | | | | | | | |  | | | |  |
| 24506 | Natural environment percentage, buffer 1000m | | | | | | | | | | | | | | | | | |  | | | |  |
| 24507 | Natural environment percentage, buffer 300m | | | | | | | | | | | | | | | | | |  | | | |  |
| 24508 | Distance (Euclidean) to coast | | | | | | | | | | | | | | | | | |  | | | |  |
| **Medical history** | | | | | | | | | | | | | | | | | | | | | | | |
| **Field ID** | **Description** | | | | | **Coding** | | | | | | | | | | | | | | **Coding notes** | | | |
| 2178 | Overall health rating | | | | | 100508 | | | | | | 1 | | | Excellent | | | | |  | | |  |
|  |  |  |  |  |  |  |  |  |  |  |  | 2 | | | Good | | | | |  | | |  |
|  |  |  |  |  |  |  |  |  |  |  |  | 3 | | | Fair | | | | |  | | |  |
|  |  |  |  |  |  |  |  |  |  |  |  | 4 | | | Poor | | | | |  | | |  |
|  |  |  |  |  |  |  |  |  |  |  |  | -1 | | | Do not know | | | | | set as NA | | |  |
|  |  |  |  |  |  |  |  |  |  |  |  | -3 | | | Prefer not to answer | | | | | set as NA | | |  |
| 2188 | Long-standing illness, disability or infirmity | | | | | 100349 | | | | | | 1 | | | Yes | | | | |  | | |  |
|  |  |  |  |  |  |  |  |  |  |  |  | 0 | | | No | | | | |  | | |  |
|  |  |  |  |  |  |  |  |  |  |  |  | -1 | | | Do not know | | | | | set as NA | | |  |
|  |  |  |  |  |  |  |  |  |  |  |  | -3 | | | Prefer not to answer | | | | | set as NA | | |  |
| 2296 | Falls in the last year | | | | | 100539 | | | | | | 1 | | | No falls | | | | | set as 0 | | | 1: fall;  0: no fall |
|  |  |  |  |  |  |  |  |  |  |  |  | 2 | | | Only one fall | | | | | set as 0 | | |  |
|  |  |  |  |  |  |  |  |  |  |  |  | 3 | | | More than one fall | | | | | set as 1 | | |  |
|  |  |  |  |  |  |  |  |  |  |  |  | -3 | | | Prefer not to answer | | | | | set as NA | | |  |
| 2306 | Weight change compared with 1 year ago | | | | | 100540 | | | | | | 0 | | | No - weigh about the same | | | | |  | | |  |
|  |  |  |  |  |  |  |  |  |  |  |  | 2 | | | Yes - gained weight | | | | |  | | |  |
|  |  |  |  |  |  |  |  |  |  |  |  | 3 | | | Yes - lost weight | | | | |  | | |  |
|  |  |  |  |  |  |  |  |  |  |  |  | -1 | | | Do not know | | | | | set as NA | | |  |
|  |  |  |  |  |  |  |  |  |  |  |  | -3 | | | Prefer not to answer | | | | | set as NA | | |  |
| 2335 | Chest pain or discomfort | | | | | 100349 | | | | | | same as above | | | | | | | |  | | |  |
| 6146 | Attendance/disability/mobility allowance | | | | | 100510 | | | | | | 1 | | | Attendance allowance | | | | | set as 1 | | | 1: disability; 0: no disability |
|  |  |  |  |  |  |  |  |  |  |  |  | 2 | | | Disability living allowance | | | | | set as 1 | | |  |
|  |  |  |  |  |  |  |  |  |  |  |  | 3 | | | Blue badge | | | | | set as 1 | | |  |
|  |  |  |  |  |  |  |  |  |  |  |  | -7 | | | None of the above | | | | | set as 0 | | |  |
|  |  |  |  |  |  |  |  |  |  |  |  | -1 | | | Do not know | | | | | set as NA | | |  |
|  |  |  |  |  |  |  |  |  |  |  |  | -3 | | | Prefer not to answer | | | | | set as NA | | |  |
| 6149 | Mouth/teeth dental problems | | | | | 100538 | | | | | | 1 | | | Mouth ulcers | | | | | set as 1 | | | 1: Mouth/teeth dental problems;  0: No mouth /teeth dental problems |
|  |  |  |  |  |  |  |  |  |  |  |  | 2 | | | Painful gums | | | | | set as 1 | | |  |
|  |  |  |  |  |  |  |  |  |  |  |  | 3 | | | Bleeding gums | | | | | set as 1 | | |  |
|  |  |  |  |  |  |  |  |  |  |  |  | 4 | | | Loose teeth | | | | | set as 1 | | |  |
|  |  |  |  |  |  |  |  |  |  |  |  | 5 | | | Toothache | | | | | set as 1 | | |  |
|  |  |  |  |  |  |  |  |  |  |  |  | 6 | | | Dentures | | | | | set as 1 | | |  |
|  |  |  |  |  |  |  |  |  |  |  |  | -7 | | | None of the above | | | | | set as 0 | | |  |
|  |  |  |  |  |  |  |  |  |  |  |  | -3 | | | Prefer not to answer | | | | | set as NA | | |  |
| 130176 | Date B01 first reported (varicella [chickenpox]) | | | | | | | | | | | | | | | | | | | | | | |
| 130184 | Date B05 first reported (measles) | | | | | | | | | | | | | | | | | | | | | | |
| 130188 | Date B07 first reported (viral warts) | | | | | | | | | | | | | | | | | | | | | | |
| 130216 | Date B26 first reported (mumps) | | | | | | | | | | | | | | | | | | | | | | |
| 130226 | Date B35 first reported (dermatophytosis) | | | | | | | | | | | | | | | | | | | | | | |
| 130622 | Date D50 first reported (iron deficiency anaemia) | | | | | | | | | | | | | | | | | | | | | | |
| 130696 | Date E03 first reported (other hypothyroidism) | | | | | | | | | | | | | | | | | | | | | | |
| 130708 | Date E11 first reported (non-insulin-dependent diabetes mellitus) | | | | | | | | | | | | | | | | | | | | | | |
| 130714 | Date E14 first reported (unspecified diabetes mellitus) | | | | | | | | | | | | | | | | | | | | | | |
| 130814 | Date E78 first reported (disorders of lipoprotein metabolism and other lipidaemias) | | | | | | | | | | | | | | | | | | | | | | |
| 130868 | Date F17 first reported (mental and behavioural disorders due to use of tobacco) | | | | | | | | | | | | | | | | | | | | | | |
| 130906 | Date F41 first reported (other anxiety disorders) | | | | | | | | | | | | | | | | | | | | | | |
| 130910 | Date F43 first reported (reaction to severe stress, and adjustment disorders) | | | | | | | | | | | | | | | | | | | | | | |
| 131142 | Date H10 first reported (conjunctivitis) | | | | | | | | | | | | | | | | | | | | | | |
| 131166 | Date H26 first reported (other cataract) | | | | | | | | | | | | | | | | | | | | | | |
| 131222 | Date H60 first reported (otitis externa) | | | | | | | | | | | | | | | | | | | | | | |
| 131224 | Date H61 first reported (other disorders of external ear) | | | | | | | | | | | | | | | | | | | | | | |
| 131262 | Date H92 first reported (otalgia and effusion of ear) | | | | | | | | | | | | | | | | | | | | | | |
| 131264 | Date H93 first reported (other disorders of ear, not elsewhere classified) | | | | | | | | | | | | | | | | | | | | | | |
| 131296 | Date I20 first reported (angina pectoris) | | | | | | | | | | | | | | | | | | | | | | |
| 131298 | Date I21 first reported (acute myocardial infarction) | | | | | | | | | | | | | | | | | | | | | | |
| 131306 | Date I25 first reported (chronic ischaemic heart disease) | | | | | | | | | | | | | | | | | | | | | | |
| 131368 | Date I64 first reported (stroke, not specified as haemorrhage or infarction) | | | | | | | | | | | | | | | | | | | | | | |
| 131396 | Date I80 first reported (phlebitis and thrombophlebitis) | | | | | | | | | | | | | | | | | | | | | | |
| 131402 | Date I83 first reported (varicose veins of lower extremities) | | | | | | | | | | | | | | | | | | | | | | |
| 131404 | Date I84 first reported (haemorrhoids) | | | | | | | | | | | | | | | | | | | | | | |
| 131426 | Date J01 first reported (acute sinusitis) | | | | | | | | | | | | | | | | | | | | | | |
| 131428 | Date J02 first reported (acute pharyngitis) | | | | | | | | | | | | | | | | | | | | | | |
| 131430 | Date J03 first reported (acute tonsillitis) | | | | | | | | | | | | | | | | | | | | | | |
| 131436 | Date J06 first reported (acute upper respiratory infections of multiple and unspecified sites) | | | | | | | | | | | | | | | | | | | | | | |
| 131456 | Date J18 first reported (pneumonia, organism unspecified) | | | | | | | | | | | | | | | | | | | | | | |
| 131462 | Date J22 first reported (unspecified acute lower respiratory infection) | | | | | | | | | | | | | | | | | | | | | | |
| 131464 | Date J30 first reported (vasomotor and allergic rhinitis) | | | | | | | | | | | | | | | | | | | | | | |
| 131472 | Date J34 first reported (other disorders of nose and nasal sinuses) | | | | | | | | | | | | | | | | | | | | | | |
| 131494 | Date J45 first reported (asthma) | | | | | | | | | | | | | | | | | | | | | | |
| 131584 | Date K21 first reported (gastro-oesophageal reflux medical history) | | | | | | | | | | | | | | | | | | | | | | |
| 131598 | Date K29 first reported (gastritis and duodenitis) | | | | | | | | | | | | | | | | | | | | | | |
| 131600 | Date K30 first reported (dyspepsia)  Date K40 first reported (inguinal hernia) | | | | | | | | | | | | | | | | | | | | | | |
| 131612 |  |  |  |  |  |  |  |  |  |  |  |  |  |  |  |  |  |  |  |  |  |  |  |
| 131620 | Date K44 first reported (diaphragmatic hernia) | | | | | | | | | | | | | | | | | | | | | | |
| 131630 | Date K52 first reported (other non-infective gastro-enteritis and colitis) | | | | | | | | | | | | | | | | | | | | | | |
| 131636 | Date K57 first reported (diverticular medical history of intestine) | | | | | | | | | | | | | | | | | | | | | | |
| 131638 | Date K58 first reported (irritable bowel syndrome) | | | | | | | | | | | | | | | | | | | | | | |
| 131646 | Date K62 first reported (other Medical historys of anus and rectum) | | | | | | | | | | | | | | | | | | | | | | |
| 131650 | Date K64 first reported (haemorrhoids and perianal venous thrombosis) | | | | | | | | | | | | | | | | | | | | | | |
| 131674 | Date K80 first reported (cholelithiasis) | | | | | | | | | | | | | | | | | | | | | | |
| 131702 | Date L03 first reported (cellulitis) | | | | | | | | | | | | | | | | | | | | | | |
| 131720 | Date L20 first reported (atopic dermatitis) | | | | | | | | | | | | | | | | | | | | | | |
| 131740 | Date L30 first reported (other dermatitis) | | | | | | | | | | | | | | | | | | | | | | |
| 131742 | Date L40 first reported (psoriasis) | | | | | | | | | | | | | | | | | | | | | | |
| 131806 | Date L82 first reported (seborrhoeic keratosis) | | | | | | | | | | | | | | | | | | | | | | |
| 131836 | Date L98 first reported (other disorders of skin and subcutaneous tissue, not elsewhere classified) | | | | | | | | | | | | | | | | | | | | | | |
| 131858 | Date M10 first reported (gout) | | | | | | | | | | | | | | | | | | | | | | |
| 131864 | Date M13 first reported (other arthritis) | | | | | | | | | | | | | | | | | | | | | | |
| 131876 | Date M19 first reported (other arthrosis) | | | | | | | | | | | | | | | | | | | | | | |
| 131878 | Date M20 first reported (acquired deformities of fingers and toes) | | | | | | | | | | | | | | | | | | | | | | |
| 131884 | Date M23 first reported (internal derangement of knee) | | | | | | | | | | | | | | | | | | | | | | |
| 131888 | Date M25 first reported (other joint disorders, not elsewhere classified) | | | | | | | | | | | | | | | | | | | | | | |
| 131916 | Date M47 first reported (spondylosis) | | | | | | | | | | | | | | | | | | | | | | |
| 131924 | Date M51 first reported (other intervertebral disk disorders) | | | | | | | | | | | | | | | | | | | | | | |
| 131928 | Date M54 first reported (dorsalgia) | | | | | | | | | | | | | | | | | | | | | | |
| 131950 | Date M72 first reported (fibroblastic disorders) | | | | | | | | | | | | | | | | | | | | | | |
| 131954 | Date M75 first reported (shoulder lesions) | | | | | | | | | | | | | | | | | | | | | | |
| 131958 | Date M77 first reported (other enthesopathies) | | | | | | | | | | | | | | | | | | | | | | |
| 131960 | Date M79 first reported (other soft tissue disorders, not elsewhere classified) | | | | | | | | | | | | | | | | | | | | | | |
| 132054 | Date N30 first reported (cystitis) | | | | | | | | | | | | | | | | | | | | | | |
| 132070 | Date N39 first reported (other disorders of urinary system) | | | | | | | | | | | | | | | | | | | | | | |
| 132072 | Date N40 first reported (hyperplasia of prostate) | | | | | | | | | | | | | | | | | | | | | | |
| 132096 | Date N60 first reported (benign mammary dysplasia) | | | | | | | | | | | | | | | | | | | | | | |
| 132102 | Date N63 first reported (unspecified lump in breast) | | | | | | | | | | | | | | | | | | | | | | |
| 132124 | Date N81 first reported (female genital prolapse) | | | | | | | | | | | | | | | | | | | | | | |
| 132130 | Date N84 first reported (polyp of female genital tract) | | | | | | | | | | | | | | | | | | | | | | |
| 132146 | Date N92 first reported (excessive, frequent and irregular menstruation) | | | | | | | | | | | | | | | | | | | | | | |
| 132150 | Date N94 first reported (pain and other conditions associated with female genital organs and menstrual cycle) | | | | | | | | | | | | | | | | | | | | | | |
| 132152 | Date N95 first reported (menopausal and other perimenopausal disorders) | | | | | | | | | | | | | | | | | | | | | | |
| 132276 | Date O80 first reported (single spontaneous delivery) | | | | | | | | | | | | | | | | | | | | | | |
| 131492 | Date J44 first reported (other chronic obstructive pulmonary disease) | | | | | | | | | | | | | | | | | | | | | | |

**Supplementary Table3 Baseline characteristics of studied population**

| Characteristics | Parkinson's disease | | Total population  (n = 452492) |
| --- | --- | --- | --- |
|  | Yes (n = 3348) | No (n = 449144) |  |
| Age [years (mean ± SD) ] | 62.85 ± 5.34 | 56.48 ± 8.12 | 56.53 ± 8.12 |
| Sex[n (%)] | | | |
| female | 1202 (35.9) | 237992 (53) | 239194 (52.9) |
| male | 2146 (64.1) | 211152(47) | 213298 (47.1) |
| follow-up time [months (mean ± SD)] | 111.77 ± 42.95 | 175.16 ± 12.28 | 174.69 ± 13.88 |
| deprivation index (mean ± SD) | -1.46 ± 3.01 | -1.31 ± 3.09 | -1.31 ± 3.09 |
| Household income [n (%)] | | | |
| Less than 18,000 | 910 (27.2) | 85908 (19.1) | 86818 (19.2) |
| 18,000 to 30,999 | 813 (24.3) | 97350 (21.7) | 98163 (21.7) |
| 31000 to 51,999 | 1205 (36.0) | 165527 (36.9) | 166732 (36.8) |
| 52,000 to 100,000 | 319 (9.5) | 79174 (17.6) | 79493 (17.6) |
| Greater than 100,000 | 101 (3.0) | 21185 (4.7) | 21286 (4.7) |
| Higher education [n (%)] | | | |
| yes | 1130 (33.8) | 169179 (37.7) | 170309 (37.6) |
| no | 2218 (66.2) | 279965 (62.3) | 282183 (62.4) |
| BMI[kg/m^2^ (mean±SD)] | 27.72 ± 4.43 | 27.37 ± 4.73 | 27.38± 4.73 |

SD, stand deviation; Higher education, College or University degree and Other professional qualifications eg: nursing, teaching; BMI, body mass index.

**Supplementary Table4 Associations between risk factors and Parkinson's disease (Full sample and by age, gender and PRS)**

| Exposures | Full sample | | Age<65 | | Age>65 | | Male | | Female | | Low PRS | | Median PRS | | High PRS | | Category |
| --- | --- | --- | --- | --- | --- | --- | --- | --- | --- | --- | --- | --- | --- | --- | --- | --- | --- |
|  | HR | P | HR | P | HR | P | HR | P | HR | P | HR | P | HR | P | HR | P |  |
| Long-standing illness, disability or infirmity | 1.38 | 3.63E-20 | 1.45 | 1.49E-14 | 1.31 | 1.58E-07 | 1.38 | 1.21E-13 | 1.38 | 5.29E-08 | low | 1.03E-09 | 1.35 | 1.84E-06 | 1.34 | 1.84E-08 | Medical history |
| Falls in the last year | 1.42 | 1.15E-08 | 1.52 | 3.07E-07 | 1.31 | 3.66E-03 | 1.41 | 2.56E-05 | 1.43 | 1.15E-04 | 1.74 | 3.58E-06 | 1.48 | 2.91E-04 | 1.24 | 2.36E-02 | Medical history |
| Weight change compared with 1 year ago gained weight | 1.05 | 2.34E-01 | 1.03 | 5.76E-01 | 1.11 | 6.83E-02 | 1.04 | 4.70E-01 | 1.11 | 9.50E-02 | 1.10 | 2.42E-01 | 1.08 | 2.92E-01 | 1.05 | 3.91E-01 | Medical history |
| Weight change compared with 1 year ago lost weight | 1.11 | 3.21E-02 | 1.14 | 3.94E-02 | 1.08 | 3.04E-01 | 1.09 | 1.37E-01 | 1.15 | 7.64E-02 | 1.17 | 1.22E-01 | 1.06 | 4.87E-01 | 1.13 | 7.63E-02 | Medical history |
| Chest pain or discomfort | 1.42 | 1.43E-18 | 1.44 | 1.71E-10 | 1.39 | 1.06E-07 | 1.35 | 9.79E-09 | 1.55 | 5.43E-10 | 1.53 | 5.45E-07 | 1.56 | 1.66E-09 | 1.27 | 1.44E-04 | Medical history |
| Attendance/disability/mobility allowance | 1.49 | 3.06E-12 | 1.81 | 2.35E-14 | 1.24 | 1.05E-02 | 1.40 | 3.14E-06 | 1.68 | 4.03E-08 | 2.10 | 6.71E-13 | 1.45 | 3.34E-04 | 1.24 | 1.80E-02 | Medical history |
| Mouth/teeth dental problems | 1.01 | 7.16E-01 | 1.05 | 3.57E-01 | 0.99 | 7.73E-01 | 1.00 | 9.65E-01 | 1.04 | 5.05E-01 | 1.02 | 7.71E-01 | 1.10 | 1.26E-01 | 0.97 | 5.19E-01 | Medical history |
| COPD | 1.00 | 9.84E-01 | 1.24 | 1.49E-01 | 0.84 | 2.49E-01 | 0.96 | 7.56E-01 | 1.19 | 3.17E-01 | 1.00 | 9.96E-01 | 0.90 | 5.17E-01 | 1.09 | 5.65E-01 | Medical history |
| non-insulin-dependent diabetes | 1.62 | 6.55E-12 | 1.90 | 2.73E-10 | 1.44 | 2.09E-04 | 1.61 | 9.33E-10 | 1.50 | 5.65E-03 | 2.21 | 3.77E-10 | 1.74 | 3.74E-06 | 1.21 | 1.09E-01 | Medical history |
| unspecified diabetes mellitus | 1.45 | 2.35E-09 | 1.66 | 8.45E-09 | 1.29 | 3.61E-03 | 1.45 | 5.42E-08 | 1.32 | 3.15E-02 | 1.76 | 1.26E-06 | 1.53 | 5.49E-05 | 1.20 | 6.95E-02 | Medical history |
| varicella [chickenpox] | 0.52 | 1.41E-06 | 0.60 | 7.86E-04 | 0.32 | 3.19E-04 | 0.58 | 4.73E-04 | 0.40 | 6.85E-04 | 0.60 | 6.19E-02 | 0.37 | 5.77E-04 | 0.57 | 2.82E-03 | Medical history |
| measles | 0.50 | 7.85E-06 | 0.50 | 1.98E-04 | 0.48 | 1.13E-02 | 0.54 | 7.76E-04 | 0.40 | 2.86E-03 | 0.59 | 8.50E-02 | 0.35 | 1.70E-03 | 0.55 | 5.03E-03 | Medical history |
| viral warts | 0.83 | 1.66E-01 | 0.69 | 5.86E-02 | 1.00 | 9.88E-01 | 0.86 | 3.67E-01 | 0.78 | 2.69E-01 | 0.60 | 1.13E-01 | 0.82 | 4.11E-01 | 0.96 | 8.10E-01 | Medical history |
| mumps | 0.49 | 1.87E-04 | 0.50 | 2.60E-03 | 0.45 | 2.41E-02 | 0.50 | 2.35E-03 | 0.46 | 2.96E-02 | 0.33 | 2.90E-02 | 0.53 | 5.99E-02 | 0.51 | 1.20E-02 | Medical history |
| dermatophytosis | 1.15 | 6.90E-02 | 1.21 | 7.33E-02 | 1.09 | 4.51E-01 | 1.17 | 9.37E-02 | 1.12 | 4.58E-01 | 1.49 | 6.07E-03 | 1.05 | 7.24E-01 | 1.04 | 7.15E-01 | Medical history |
| iron deficiency anaemia | 1.17 | 2.15E-01 | 1.43 | 2.26E-02 | 0.90 | 6.11E-01 | 0.93 | 7.33E-01 | 1.37 | 4.50E-02 | 1.29 | 3.07E-01 | 1.20 | 4.07E-01 | 1.10 | 6.10E-01 | Medical history |
| other hypothyroidism | 1.16 | 5.47E-02 | 1.21 | 7.86E-02 | 1.11 | 3.41E-01 | 1.05 | 7.56E-01 | 1.21 | 3.59E-02 | 1.32 | 7.20E-02 | 1.42 | 5.55E-03 | 0.93 | 5.79E-01 | Medical history |
| disorders of lipoprotein metabolism | 1.17 | 1.57E-04 | 1.18 | 6.40E-03 | 1.16 | 8.79E-03 | 1.16 | 2.70E-03 | 1.19 | 2.04E-02 | 1.34 | 3.61E-04 | 1.18 | 2.11E-02 | 1.08 | 2.23E-01 | Medical history |
| mental and behavioural disorders due to use of tobacco | 0.85 | 7.88E-02 | 0.88 | 2.80E-01 | 0.82 | 1.43E-01 | 0.77 | 1.94E-02 | 1.06 | 6.90E-01 | 0.93 | 6.87E-01 | 0.94 | 6.70E-01 | 0.76 | 5.27E-02 | Medical history |
| other anxiety disorders | 1.44 | 2.31E-05 | 1.68 | 1.11E-06 | 1.11 | 4.88E-01 | 1.51 | 3.22E-04 | 1.36 | 1.99E-02 | 1.81 | 3.72E-04 | 1.42 | 2.64E-02 | 1.28 | 6.05E-02 | Medical history |
| reaction to severe stress, and adjustment disorders | 1.03 | 8.36E-01 | 1.10 | 5.83E-01 | 0.89 | 6.48E-01 | 0.99 | 9.54E-01 | 1.08 | 7.17E-01 | 1.19 | 5.44E-01 | 1.11 | 6.60E-01 | 0.89 | 6.05E-01 | Medical history |
| conjunctivitis | 0.93 | 4.92E-01 | 1.00 | 9.81E-01 | 0.86 | 3.16E-01 | 1.00 | 9.97E-01 | 0.85 | 3.03E-01 | 1.12 | 5.62E-01 | 0.94 | 7.49E-01 | 0.84 | 2.41E-01 | Medical history |
| other cataract | 1.05 | 5.43E-01 | 1.16 | 2.99E-01 | 1.01 | 8.94E-01 | 1.01 | 8.96E-01 | 1.12 | 4.16E-01 | 1.19 | 3.07E-01 | 1.02 | 9.13E-01 | 1.01 | 9.13E-01 | Medical history |
| otitis externa | 1.19 | 7.57E-02 | 0.98 | 8.97E-01 | 1.43 | 7.35E-03 | 1.16 | 2.27E-01 | 1.27 | 1.69E-01 | 1.24 | 2.98E-01 | 1.29 | 1.49E-01 | 1.10 | 5.26E-01 | Medical history |
| other disorders of external ear | 0.98 | 8.33E-01 | 1.07 | 5.99E-01 | 0.91 | 4.42E-01 | 0.94 | 5.45E-01 | 1.10 | 5.68E-01 | 1.55 | 3.78E-03 | 0.79 | 1.87E-01 | 0.83 | 1.90E-01 | Medical history |
| otalgia and effusion of ear | 1.18 | 1.82E-01 | 0.93 | 7.10E-01 | 1.47 | 1.80E-02 | 1.03 | 8.52E-01 | 1.36 | 7.59E-02 | 1.18 | 5.28E-01 | 1.05 | 8.35E-01 | 1.26 | 1.83E-01 | Medical history |
| other disorders of ear, not elsewhere classified | 1.04 | 7.31E-01 | 1.09 | 5.60E-01 | 0.98 | 9.10E-01 | 1.14 | 2.50E-01 | 0.79 | 2.65E-01 | 1.36 | 1.01E-01 | 1.13 | 4.76E-01 | 0.81 | 2.21E-01 | Medical history |
| angina pectoris | 1.49 | 6.92E-12 | 1.47 | 4.14E-05 | 1.52 | 1.69E-08 | 1.42 | 1.52E-07 | 1.74 | 1.81E-06 | 1.64 | 1.56E-05 | 1.54 | 3.52E-05 | 1.39 | 1.98E-04 | Medical history |
| acute myocardial infarction | 0.96 | 6.10E-01 | 0.87 | 3.48E-01 | 1.02 | 8.51E-01 | 0.91 | 3.37E-01 | 1.29 | 2.51E-01 | 1.30 | 7.95E-02 | 0.97 | 8.37E-01 | 0.77 | 7.21E-02 | Medical history |
| chronic ischaemic heart disease | 1.36 | 1.44E-06 | 1.38 | 2.09E-03 | 1.37 | 1.24E-04 | 1.32 | 9.13E-05 | 1.61 | 1.71E-03 | 1.56 | 3.02E-04 | 1.30 | 2.60E-02 | 1.31 | 5.81E-03 | Medical history |
| stroke, not specified as haemorrhage or infarction | 1.55 | 6.32E-06 | 2.25 | 6.79E-10 | 1.13 | 3.84E-01 | 1.56 | 6.24E-05 | 1.52 | 3.73E-02 | 1.90 | 3.46E-04 | 1.43 | 4.88E-02 | 1.48 | 9.56E-03 | Medical history |
| phlebitis and thrombophlebitis | 1.04 | 7.26E-01 | 1.06 | 6.90E-01 | 1.02 | 8.93E-01 | 1.01 | 9.20E-01 | 1.07 | 6.70E-01 | 1.21 | 3.37E-01 | 0.93 | 6.89E-01 | 1.04 | 8.10E-01 | Medical history |
| varicose veins of lower extremities | 0.96 | 6.78E-01 | 0.87 | 3.36E-01 | 1.04 | 7.57E-01 | 0.95 | 6.70E-01 | 0.98 | 8.78E-01 | 0.90 | 6.00E-01 | 1.07 | 6.78E-01 | 0.91 | 5.38E-01 | Medical history |
| haemorrhoids | 1.10 | 3.01E-01 | 1.11 | 4.32E-01 | 1.09 | 4.97E-01 | 1.17 | 1.47E-01 | 0.95 | 7.77E-01 | 1.48 | 2.25E-02 | 1.21 | 2.27E-01 | 0.85 | 2.84E-01 | Medical history |
| acute sinusitis | 1.09 | 3.99E-01 | 0.99 | 9.31E-01 | 1.20 | 1.86E-01 | 0.97 | 8.44E-01 | 1.20 | 1.66E-01 | 1.01 | 9.66E-01 | 1.21 | 2.49E-01 | 1.07 | 6.71E-01 | Medical history |
| acute pharyngitis | 0.92 | 4.54E-01 | 0.90 | 4.50E-01 | 0.96 | 7.78E-01 | 0.85 | 2.66E-01 | 1.02 | 8.91E-01 | 1.01 | 9.56E-01 | 0.96 | 8.26E-01 | 0.85 | 3.26E-01 | Medical history |
| acute tonsillitis | 0.80 | 7.82E-02 | 0.80 | 1.50E-01 | 0.82 | 3.11E-01 | 0.90 | 4.84E-01 | 0.66 | 5.29E-02 | 0.76 | 3.02E-01 | 1.03 | 8.70E-01 | 0.68 | 4.87E-02 | Medical history |
| acute upper respiratory infections of multiple | 1.05 | 5.46E-01 | 1.09 | 3.79E-01 | 0.99 | 9.42E-01 | 1.07 | 4.56E-01 | 1.00 | 9.67E-01 | 1.10 | 5.30E-01 | 1.08 | 5.33E-01 | 1.00 | 9.88E-01 | Medical history |
| pneumonia, organism unspecified | 0.93 | 4.92E-01 | 0.92 | 5.93E-01 | 0.94 | 6.64E-01 | 0.77 | 6.68E-02 | 1.29 | 1.29E-01 | 0.86 | 5.23E-01 | 1.26 | 1.69E-01 | 0.75 | 1.02E-01 | Medical history |
| unspecified acute lower respiratory infection | 1.01 | 8.86E-01 | 1.12 | 2.40E-01 | 0.91 | 3.45E-01 | 0.94 | 5.12E-01 | 1.13 | 2.70E-01 | 1.13 | 3.50E-01 | 0.89 | 3.91E-01 | 1.04 | 7.28E-01 | Medical history |
| vasomotor and allergic rhinitis | 0.98 | 7.66E-01 | 0.94 | 5.15E-01 | 1.03 | 7.45E-01 | 1.03 | 7.39E-01 | 0.89 | 3.45E-01 | 0.91 | 5.46E-01 | 0.97 | 7.98E-01 | 1.01 | 9.20E-01 | Medical history |
| other disorders of nose and nasal sinuses | 1.05 | 6.71E-01 | 1.12 | 4.46E-01 | 0.96 | 8.04E-01 | 1.11 | 3.90E-01 | 0.88 | 5.63E-01 | 1.21 | 3.51E-01 | 1.19 | 3.52E-01 | 0.89 | 5.04E-01 | Medical history |
| asthma | 1.09 | 1.14E-01 | 1.07 | 3.45E-01 | 1.11 | 1.66E-01 | 1.06 | 3.88E-01 | 1.13 | 1.46E-01 | 1.11 | 3.34E-01 | 1.08 | 4.23E-01 | 1.10 | 2.18E-01 | Medical history |
| gastro-oesophageal reflux Medical history | 1.12 | 6.81E-02 | 1.11 | 2.21E-01 | 1.12 | 1.78E-01 | 1.13 | 9.04E-02 | 1.08 | 4.31E-01 | 1.01 | 9.37E-01 | 1.15 | 1.81E-01 | 1.15 | 1.20E-01 | Medical history |
| gastritis and duodenitis | 1.10 | 2.02E-01 | 1.16 | 1.41E-01 | 1.04 | 6.80E-01 | 1.12 | 2.12E-01 | 1.06 | 6.56E-01 | 1.22 | 1.69E-01 | 0.81 | 1.63E-01 | 1.24 | 3.45E-02 | Medical history |
| dyspepsia | 1.21 | 7.90E-02 | 1.23 | 1.72E-01 | 1.18 | 2.58E-01 | 1.08 | 6.19E-01 | 1.38 | 3.66E-02 | 1.35 | 1.71E-01 | 1.01 | 9.49E-01 | 1.28 | 1.10E-01 | Medical history |
| inguinal hernia | 1.13 | 8.45E-02 | 1.06 | 6.29E-01 | 1.20 | 5.84E-02 | 1.14 | 7.75E-02 | 0.98 | 9.60E-01 | 0.98 | 8.80E-01 | 1.10 | 4.92E-01 | 1.23 | 4.62E-02 | Medical history |
| diaphragmatic hernia | 1.16 | 3.65E-02 | 1.24 | 3.41E-02 | 1.09 | 3.45E-01 | 1.16 | 9.86E-02 | 1.16 | 1.99E-01 | 1.24 | 1.10E-01 | 1.14 | 2.89E-01 | 1.13 | 2.24E-01 | Medical history |
| other non-infective gastro-enteritis and colitis | 1.09 | 3.79E-01 | 1.10 | 4.84E-01 | 1.08 | 5.74E-01 | 1.01 | 9.69E-01 | 1.21 | 1.91E-01 | 1.29 | 1.86E-01 | 1.04 | 8.19E-01 | 1.03 | 8.65E-01 | Medical history |
| diverticular Medical history of intestine | 1.06 | 4.97E-01 | 0.93 | 5.90E-01 | 1.14 | 1.72E-01 | 1.07 | 4.93E-01 | 1.03 | 8.17E-01 | 1.36 | 3.85E-02 | 0.96 | 7.91E-01 | 0.97 | 7.77E-01 | Medical history |
| irritable bowel syndrome | 1.03 | 7.30E-01 | 1.13 | 2.66E-01 | 0.89 | 4.29E-01 | 0.98 | 8.79E-01 | 1.07 | 5.49E-01 | 0.78 | 2.49E-01 | 0.95 | 7.51E-01 | 1.21 | 1.16E-01 | Medical history |
| other Medical historys of anus and rectum | 1.22 | 6.75E-03 | 1.25 | 3.42E-02 | 1.20 | 8.67E-02 | 1.25 | 1.31E-02 | 1.17 | 2.33E-01 | 1.47 | 7.06E-03 | 1.04 | 7.83E-01 | 1.22 | 7.08E-02 | Medical history |
| haemorrhoids and perianal venous thrombosis | 1.08 | 3.19E-01 | 1.09 | 4.66E-01 | 1.08 | 5.17E-01 | 1.10 | 3.22E-01 | 1.05 | 7.61E-01 | 1.06 | 7.11E-01 | 0.92 | 5.86E-01 | 1.21 | 9.55E-02 | Medical history |
| cholelithiasis | 1.19 | 4.63E-02 | 1.27 | 5.63E-02 | 1.13 | 3.39E-01 | 1.32 | 2.89E-02 | 1.09 | 4.64E-01 | 1.02 | 9.01E-01 | 1.10 | 5.76E-01 | 1.37 | 1.11E-02 | Medical history |
| cellulitis | 1.14 | 1.51E-01 | 1.26 | 6.72E-02 | 1.03 | 8.53E-01 | 1.08 | 5.24E-01 | 1.29 | 1.04E-01 | 1.63 | 2.64E-03 | 1.17 | 3.49E-01 | 0.89 | 4.41E-01 | Medical history |
| atopic dermatitis | 1.21 | 1.10E-01 | 1.32 | 6.67E-02 | 1.07 | 7.16E-01 | 1.09 | 5.74E-01 | 1.39 | 6.32E-02 | 1.47 | 7.93E-02 | 1.07 | 7.67E-01 | 1.16 | 3.95E-01 | Medical history |
| other dermatitis | 0.96 | 5.31E-01 | 0.90 | 3.16E-01 | 1.02 | 8.69E-01 | 0.98 | 7.97E-01 | 0.92 | 4.78E-01 | 1.43 | 5.24E-03 | 0.79 | 1.05E-01 | 0.84 | 1.16E-01 | Medical history |
| psoriasis | 0.93 | 5.53E-01 | 0.87 | 4.02E-01 | 1.00 | 9.93E-01 | 0.93 | 5.98E-01 | 0.94 | 7.78E-01 | 1.45 | 6.79E-02 | 0.77 | 2.74E-01 | 0.78 | 2.03E-01 | Medical history |
| seborrhoeic keratosis | 0.84 | 8.19E-02 | 0.71 | 3.42E-02 | 0.94 | 6.51E-01 | 0.76 | 3.84E-02 | 0.97 | 8.59E-01 | 0.92 | 6.87E-01 | 0.58 | 1.49E-02 | 0.96 | 7.62E-01 | Medical history |
| other disorders of skin and subcutaneous tissue, not elsewhere classified | 1.01 | 8.60E-01 | 1.08 | 4.90E-01 | 0.95 | 6.42E-01 | 0.96 | 6.51E-01 | 1.11 | 3.96E-01 | 1.05 | 7.40E-01 | 1.05 | 7.53E-01 | 0.97 | 8.16E-01 | Medical history |
| gout | 0.79 | 2.48E-02 | 0.92 | 5.56E-01 | 0.69 | 1.29E-02 | 0.79 | 2.62E-02 | 0.86 | 7.07E-01 | 1.12 | 5.13E-01 | 0.55 | 8.45E-03 | 0.77 | 9.58E-02 | Medical history |
| other arthritis | 1.07 | 4.95E-01 | 1.06 | 6.88E-01 | 1.07 | 5.78E-01 | 1.21 | 1.06E-01 | 0.87 | 3.88E-01 | 1.43 | 4.33E-02 | 0.97 | 8.71E-01 | 0.95 | 7.47E-01 | Medical history |
| gonarthrosis [arthrosis of knee] | 1.15 | 8.50E-02 | 1.15 | 2.75E-01 | 1.16 | 1.68E-01 | 1.11 | 3.31E-01 | 1.25 | 1.08E-01 | 1.36 | 5.48E-02 | 1.11 | 4.84E-01 | 1.09 | 4.97E-01 | Medical history |
| other arthrosis | 1.08 | 1.14E-01 | 1.19 | 1.37E-02 | 0.99 | 8.92E-01 | 1.06 | 3.77E-01 | 1.11 | 1.62E-01 | 1.34 | 2.90E-03 | 1.09 | 3.54E-01 | 0.97 | 6.74E-01 | Medical history |
| acquired deformities of fingers and toes | 0.94 | 5.92E-01 | 1.16 | 3.54E-01 | 0.72 | 9.36E-02 | 1.05 | 8.13E-01 | 0.88 | 4.18E-01 | 1.13 | 6.22E-01 | 0.95 | 8.29E-01 | 0.84 | 3.67E-01 | Medical history |
| internal derangement of knee | 1.05 | 6.47E-01 | 1.07 | 6.35E-01 | 1.02 | 9.06E-01 | 0.95 | 7.20E-01 | 1.33 | 1.39E-01 | 1.36 | 1.38E-01 | 0.63 | 7.71E-02 | 1.19 | 2.67E-01 | Medical history |
| other joint disorders, not elsewhere classified | 0.96 | 4.80E-01 | 0.98 | 7.98E-01 | 0.94 | 4.25E-01 | 0.96 | 5.43E-01 | 0.97 | 7.07E-01 | 1.16 | 1.75E-01 | 0.96 | 6.77E-01 | 0.87 | 1.04E-01 | Medical history |
| spondylosis | 1.28 | 1.43E-03 | 1.31 | 2.14E-02 | 1.26 | 2.73E-02 | 1.22 | 5.27E-02 | 1.38 | 7.47E-03 | 1.04 | 8.06E-01 | 1.29 | 6.75E-02 | 1.40 | 2.13E-03 | Medical history |
| other intervertebral disk disorders | 1.27 | 5.88E-03 | 1.20 | 1.47E-01 | 1.35 | 1.43E-02 | 1.26 | 3.44E-02 | 1.30 | 7.58E-02 | 1.29 | 1.61E-01 | 1.20 | 2.72E-01 | 1.31 | 3.22E-02 | Medical history |
| dorsalgia | 1.14 | 2.21E-02 | 1.15 | 6.96E-02 | 1.12 | 1.77E-01 | 1.13 | 9.86E-02 | 1.16 | 1.08E-01 | 1.14 | 2.76E-01 | 1.01 | 9.23E-01 | 1.23 | 1.15E-02 | Medical history |
| fibroblastic disorders | 1.24 | 1.84E-02 | 1.19 | 2.19E-01 | 1.29 | 3.76E-02 | 1.33 | 5.95E-03 | 0.98 | 9.37E-01 | 1.29 | 1.63E-01 | 1.18 | 3.49E-01 | 1.26 | 8.52E-02 | Medical history |
| shoulder lesions | 1.20 | 2.56E-02 | 1.05 | 6.84E-01 | 1.34 | 8.21E-03 | 1.18 | 1.10E-01 | 1.24 | 1.14E-01 | 1.33 | 8.13E-02 | 1.19 | 2.57E-01 | 1.15 | 2.59E-01 | Medical history |
| other enthesopathies | 0.89 | 2.04E-01 | 0.86 | 2.26E-01 | 0.92 | 5.16E-01 | 0.88 | 2.39E-01 | 0.92 | 5.87E-01 | 0.83 | 3.36E-01 | 1.01 | 9.47E-01 | 0.85 | 2.23E-01 | Medical history |
| other soft tissue disorders, not elsewhere classified | 1.11 | 6.13E-02 | 1.14 | 1.06E-01 | 1.09 | 3.18E-01 | 1.13 | 1.00E-01 | 1.09 | 3.48E-01 | 1.28 | 3.40E-02 | 1.12 | 2.52E-01 | 1.03 | 7.54E-01 | Medical history |
| cystitis | 1.15 | 2.41E-01 | 1.18 | 3.30E-01 | 1.13 | 4.97E-01 | 1.20 | 4.37E-01 | 1.14 | 3.72E-01 | 1.06 | 8.23E-01 | 1.47 | 5.23E-02 | 1.01 | 9.71E-01 | Medical history |
| other disorders of urinary system | 1.18 | 2.10E-02 | 1.09 | 4.50E-01 | 1.29 | 1.14E-02 | 1.32 | 1.03E-02 | 1.09 | 3.98E-01 | 1.14 | 3.93E-01 | 1.45 | 2.06E-03 | 1.05 | 6.61E-01 | Medical history |
| unspecified lump in breast | 0.98 | 8.92E-01 | 0.62 | 3.42E-02 | 1.56 | 1.59E-02 | 0.70 | 6.13E-01 | 1.00 | 9.77E-01 | 0.96 | 8.93E-01 | 0.98 | 9.40E-01 | 0.97 | 8.98E-01 | Medical history |
| menopausal and other perimenopausal disorders | 0.99 | 8.72E-01 | 0.88 | 3.50E-01 | 1.09 | 5.24E-01 | 5.43 | 9.07E-02 | 0.98 | 8.08E-01 | 0.92 | 6.97E-01 | 1.04 | 8.02E-01 | 0.97 | 8.35E-01 | Medical history |
| Breastfed as a baby | 0.98 | 7.39E-01 | 0.93 | 2.73E-01 | 1.06 | 4.78E-01 | 0.94 | 3.75E-01 | 1.04 | 6.26E-01 | 0.90 | 3.12E-01 | 1.00 | 9.94E-01 | 1.01 | 8.67E-01 | Early life |
| Comparative body size at age 10 Thinner | 1.00 | 1.00E+00 | 1.00 | 9.72E-01 | 0.98 | 7.60E-01 | 0.96 | 3.70E-01 | 1.06 | 3.76E-01 | 1.02 | 7.78E-01 | 0.98 | 7.26E-01 | 0.99 | 8.64E-01 | Early life |
| Comparative body size at age 10 Plumper | 1.13 | 1.68E-02 | 1.11 | 1.25E-01 | 1.15 | 5.98E-02 | 1.14 | 4.66E-02 | 1.12 | 1.40E-01 | 1.10 | 3.56E-01 | 1.11 | 2.29E-01 | 1.17 | 3.16E-02 | Early life |
| Comparative height size at age 10 Shorter | 0.96 | 3.48E-01 | 0.98 | 6.90E-01 | 0.93 | 2.90E-01 | 0.92 | 1.74E-01 | 1.00 | 9.65E-01 | 0.96 | 6.31E-01 | 0.95 | 5.23E-01 | 0.96 | 5.71E-01 | Early life |
| Comparative height size at age 10 Taller | 1.09 | 2.90E-02 | 1.10 | 8.28E-02 | 1.06 | 3.37E-01 | 1.10 | 6.64E-02 | 1.06 | 4.13E-01 | 1.08 | 3.43E-01 | 1.04 | 6.21E-01 | 1.10 | 9.04E-02 | Early life |
| Right-handed | 1.08 | 5.50E-01 | 0.89 | 4.58E-01 | 1.19 | 3.57E-01 | 1.12 | 4.64E-01 | 0.83 | 3.69E-01 | 1.27 | 4.18E-01 | 0.77 | 1.81E-01 | 1.17 | 4.02E-01 | Early life |
| Left-handed | 1.08 | 5.98E-01 | 0.94 | 7.41E-01 | 1.11 | 5.91E-01 | 1.09 | 5.83E-01 | 0.89 | 6.04E-01 | 1.22 | 5.21E-01 | 0.83 | 3.86E-01 | 1.15 | 4.93E-01 | Early life |
| Adopted as a child | 1.01 | 9.25E-01 | 1.09 | 6.31E-01 | 0.89 | 6.16E-01 | 0.96 | 8.19E-01 | 1.11 | 6.41E-01 | 0.99 | 9.64E-01 | 0.94 | 8.17E-01 | 1.08 | 6.94E-01 | Early life |
| Part of a multiple birth | 1.03 | 7.88E-01 | 1.05 | 7.63E-01 | 1.01 | 9.52E-01 | 1.02 | 8.95E-01 | 1.05 | 7.88E-01 | 1.02 | 9.22E-01 | 1.20 | 3.65E-01 | 0.94 | 7.30E-01 | Early life |
| Maternal smoking around birth | 1.00 | 9.66E-01 | 1.00 | 9.55E-01 | 1.00 | 9.69E-01 | 1.02 | 6.79E-01 | 0.97 | 6.23E-01 | 1.00 | 9.96E-01 | 0.96 | 6.33E-01 | 1.04 | 5.48E-01 | Early life |
| Seldom visit friends/family | 1.06 | 1.63E-01 | 1.04 | 4.68E-01 | 1.09 | 1.69E-01 | 1.08 | 1.23E-01 | 1.01 | 8.64E-01 | 0.98 | 8.00E-01 | 1.17 | 3.48E-02 | 1.03 | 6.57E-01 | Psychosocial factors |
| Mood swings | 1.13 | 4.50E-04 | 1.14 | 4.68E-03 | 1.12 | 2.90E-02 | 1.07 | 1.52E-01 | 1.26 | 8.89E-05 | 1.13 | 1.09E-01 | 1.09 | 1.62E-01 | 1.17 | 2.18E-03 | Psychosocial factors |
| Miserableness | 1.18 | 6.05E-06 | 1.21 | 7.60E-05 | 1.14 | 1.63E-02 | 1.16 | 1.56E-03 | 1.21 | 1.01E-03 | 1.17 | 3.72E-02 | 1.15 | 3.31E-02 | 1.21 | 3.11E-04 | Psychosocial factors |
| Irritability | 1.00 | 9.90E-01 | 1.03 | 6.40E-01 | 0.97 | 6.39E-01 | 0.98 | 7.54E-01 | 1.03 | 6.31E-01 | 0.95 | 5.22E-01 | 1.03 | 7.01E-01 | 1.02 | 7.86E-01 | Psychosocial factors |
| Sensitivity / hurt feelings | 1.10 | 5.50E-03 | 1.20 | 1.67E-04 | 1.00 | 9.83E-01 | 1.13 | 4.46E-03 | 1.05 | 4.20E-01 | 1.11 | 1.60E-01 | 1.04 | 5.57E-01 | 1.16 | 5.00E-03 | Psychosocial factors |
| Fed-up feelings | 1.21 | 1.17E-07 | 1.25 | 3.70E-06 | 1.16 | 4.42E-03 | 1.17 | 3.80E-04 | 1.27 | 4.88E-05 | 1.33 | 1.13E-04 | 1.18 | 1.17E-02 | 1.19 | 1.13E-03 | Psychosocial factors |
| Nervous feelings | 1.39 | 4.42E-17 | 1.53 | 6.17E-16 | 1.24 | 4.38E-04 | 1.40 | 6.10E-11 | 1.38 | 1.22E-07 | 1.20 | 3.47E-02 | 1.44 | 2.34E-07 | 1.46 | 2.32E-11 | Psychosocial factors |
| Worrier / anxious feelings | 1.32 | 9.41E-15 | 1.44 | 3.10E-13 | 1.20 | 4.60E-04 | 1.30 | 9.03E-10 | 1.35 | 1.99E-06 | 1.29 | 5.90E-04 | 1.33 | 1.19E-05 | 1.33 | 6.11E-08 | Psychosocial factors |
| Tense / 'highly strung' | 1.25 | 8.93E-07 | 1.31 | 6.05E-06 | 1.17 | 2.01E-02 | 1.21 | 1.25E-03 | 1.30 | 1.46E-04 | 1.21 | 5.19E-02 | 1.20 | 2.76E-02 | 1.30 | 6.79E-05 | Psychosocial factors |
| Worry too long after embarrassment | 1.08 | 2.54E-02 | 1.14 | 6.63E-03 | 1.01 | 8.04E-01 | 1.08 | 7.13E-02 | 1.08 | 1.86E-01 | 1.08 | 3.15E-01 | 1.03 | 6.90E-01 | 1.13 | 2.01E-02 | Psychosocial factors |
| Suffer from 'nerves' | 1.42 | 5.48E-18 | 1.49 | 1.29E-13 | 1.34 | 4.31E-06 | 1.41 | 3.26E-11 | 1.45 | 2.70E-08 | 1.30 | 2.77E-03 | 1.43 | 9.20E-07 | 1.49 | 1.24E-11 | Psychosocial factors |
| Loneliness, isolation | 1.30 | 4.98E-09 | 1.35 | 3.95E-07 | 1.24 | 1.81E-03 | 1.27 | 9.35E-05 | 1.35 | 1.05E-05 | 1.33 | 2.22E-03 | 1.31 | 7.25E-04 | 1.28 | 1.57E-04 | Psychosocial factors |
| Guilty feelings | 1.18 | 3.53E-05 | 1.27 | 5.57E-06 | 1.07 | 2.49E-01 | 1.19 | 9.24E-04 | 1.16 | 1.24E-02 | 1.24 | 9.17E-03 | 1.20 | 9.80E-03 | 1.14 | 2.30E-02 | Psychosocial factors |
| Risk taking | 0.93 | 6.83E-02 | 0.93 | 1.62E-01 | 0.93 | 2.42E-01 | 0.93 | 1.22E-01 | 0.93 | 3.31E-01 | 0.91 | 2.57E-01 | 0.96 | 6.05E-01 | 0.91 | 1.33E-01 | Psychosocial factors |
| Several days depressed mood in last 2 weeks | 1.28 | 1.22E-07 | 1.23 | 4.83E-04 | 1.31 | 8.66E-05 | 1.20 | 3.40E-03 | 1.35 | 1.10E-05 | 1.25 | 2.00E-02 | 1.35 | 1.97E-04 | 1.21 | 3.98E-03 | Psychosocial factors |
| More than half the days depressed mood in last 2 weeks | 1.38 | 2.31E-03 | 1.60 | 1.10E-04 | 1.01 | 9.63E-01 | 1.29 | 7.18E-02 | 1.46 | 1.16E-02 | 1.87 | 7.92E-04 | 1.21 | 3.31E-01 | 1.22 | 2.08E-01 | Psychosocial factors |
| Nearly every day depressed mood in last 2 weeks | 1.63 | 8.67E-05 | 1.93 | 1.68E-06 | 0.97 | 9.00E-01 | 1.73 | 3.36E-04 | 1.39 | 9.99E-02 | 2.06 | 1.46E-03 | 1.92 | 1.07E-03 | 1.15 | 5.08E-01 | Psychosocial factors |
| Several days unenthusiasm / disinterest in last 2 weeks | 1.22 | 3.52E-05 | 1.25 | 2.36E-04 | 1.17 | 3.62E-02 | 1.18 | 6.20E-03 | 1.27 | 1.58E-03 | 1.40 | 3.99E-04 | 1.25 | 6.79E-03 | 1.10 | 1.83E-01 | Psychosocial factors |
| More than half the days unenthusiasm / disinterest in last 2 weeks | 1.36 | 3.39E-03 | 1.28 | 6.57E-02 | 1.42 | 2.75E-02 | 1.42 | 5.16E-03 | 1.17 | 3.72E-01 | 1.52 | 4.53E-02 | 1.18 | 3.86E-01 | 1.33 | 5.64E-02 | Psychosocial factors |
| Nearly every day unenthusiasm / disinterest in last 2 weeks | 1.42 | 3.44E-03 | 1.56 | 1.57E-03 | 1.15 | 4.98E-01 | 1.34 | 4.60E-02 | 1.47 | 3.84E-02 | 1.94 | 1.77E-03 | 1.07 | 7.80E-01 | 1.33 | 9.58E-02 | Psychosocial factors |
| Several days tenseness / restlessness in last 2 weeks | 1.27 | 2.59E-08 | 1.30 | 2.07E-06 | 1.20 | 5.60E-03 | 1.22 | 2.92E-04 | 1.31 | 4.44E-05 | 1.34 | 7.43E-04 | 1.28 | 8.90E-04 | 1.19 | 4.56E-03 | Psychosocial factors |
| More than half the days tenseness / restlessness in last 2 weeks | 1.34 | 8.48E-03 | 1.32 | 4.33E-02 | 1.47 | 1.86E-02 | 1.36 | 2.34E-02 | 1.38 | 5.31E-02 | 1.57 | 3.50E-02 | 1.34 | 1.27E-01 | 1.29 | 1.05E-01 | Psychosocial factors |
| Nearly every day tenseness / restlessness in last 2 weeks | 1.89 | 4.32E-08 | 2.22 | 1.31E-09 | 1.20 | 4.32E-01 | 1.93 | 3.97E-06 | 1.67 | 6.51E-03 | 2.25 | 2.67E-04 | 1.75 | 6.71E-03 | 1.68 | 2.39E-03 | Psychosocial factors |
| Several days tiredness / lethargy in last 2 weeks | 1.21 | 3.33E-07 | 1.18 | 9.00E-04 | 1.23 | 7.24E-05 | 1.25 | 1.66E-06 | 1.14 | 3.52E-02 | 1.31 | 5.13E-04 | 1.17 | 2.01E-02 | 1.19 | 1.22E-03 | Psychosocial factors |
| More than half the days tiredness / lethargy in last 2 weeks | 1.32 | 3.44E-04 | 1.36 | 1.61E-03 | 1.33 | 1.33E-02 | 1.30 | 5.89E-03 | 1.37 | 5.71E-03 | 1.67 | 4.14E-04 | 1.18 | 2.20E-01 | 1.30 | 1.77E-02 | Psychosocial factors |
| Nearly every day tiredness / lethargy in last 2 weeks | 1.62 | 1.30E-11 | 1.75 | 7.29E-11 | 1.49 | 3.23E-04 | 1.62 | 8.72E-08 | 1.65 | 1.05E-06 | 2.16 | 4.52E-09 | 1.53 | 6.35E-04 | 1.50 | 9.02E-05 | Psychosocial factors |
| Seen doctor (GP) for nerves, anxiety, tension or depression | 1.35 | 1.34E-16 | 1.43 | 2.35E-13 | 1.25 | 5.89E-05 | 1.33 | 1.41E-09 | 1.39 | 1.64E-08 | 1.50 | 1.27E-07 | 1.32 | 3.46E-05 | 1.32 | 3.31E-07 | Psychosocial factors |
| Seen a psychiatrist for nerves, anxiety, tension or depression | 1.56 | 5.90E-20 | 1.68 | 1.18E-16 | 1.39 | 1.50E-05 | 1.48 | 5.33E-10 | 1.68 | 6.96E-12 | 1.76 | 5.91E-09 | 1.61 | 3.17E-08 | 1.42 | 1.42E-06 | Psychosocial factors |
| Rarely able to confide | 0.96 | 3.42E-01 | 0.91 | 9.10E-02 | 1.02 | 6.71E-01 | 0.98 | 5.98E-01 | 0.94 | 3.70E-01 | 1.17 | 4.60E-02 | 0.91 | 1.81E-01 | 0.90 | 7.83E-02 | Psychosocial factors |
| SBP | 1.00 | 3.36E-04 | 1.00 | 1.67E-03 | 1.00 | 4.48E-02 | 0.99 | 1.95E-05 | 1.00 | 8.66E-01 | 0.99 | 1.91E-04 | 1.00 | 9.75E-01 | 1.00 | 2.17E-02 | Physical measures |
| DBP | 0.99 | 2.62E-03 | 0.99 | 7.08E-03 | 1.00 | 7.69E-02 | 0.99 | 8.35E-06 | 1.00 | 7.13E-01 | 0.99 | 2.49E-04 | 1.00 | 4.46E-01 | 1.00 | 1.93E-01 | Physical measures |
| Pulse | 1.00 | 2.52E-01 | 1.00 | 2.08E-02 | 1.00 | 4.47E-01 | 1.00 | 3.69E-01 | 1.00 | 1.66E-01 | 1.00 | 6.02E-01 | 1.01 | 1.72E-02 | 1.00 | 8.53E-01 | Physical measures |
| Leg | 1.00 | 1.72E-01 | 1.01 | 6.82E-02 | 1.00 | 9.34E-01 | 1.00 | 8.96E-01 | 1.01 | 4.64E-02 | 1.02 | 1.37E-03 | 1.01 | 1.42E-01 | 1.00 | 4.53E-01 | Physical measures |
| Arm | 1.01 | 8.40E-05 | 1.01 | 1.69E-04 | 1.01 | 9.73E-02 | 1.01 | 2.65E-02 | 1.01 | 8.34E-04 | 1.03 | 1.54E-06 | 1.01 | 1.28E-02 | 1.00 | 2.40E-01 | Physical measures |
| Hand | 0.98 | 1.59E-24 | 0.98 | 8.62E-15 | 0.98 | 8.91E-12 | 0.98 | 1.04E-18 | 0.97 | 2.56E-10 | 0.97 | 1.88E-12 | 0.97 | 3.51E-10 | 0.98 | 1.53E-07 | Physical measures |
| Lung function | 0.94 | 1.22E-02 | 0.92 | 1.17E-02 | 0.96 | 2.79E-01 | 0.96 | 1.15E-01 | 0.87 | 6.45E-03 | 0.86 | 3.92E-03 | 0.92 | 6.36E-02 | 0.99 | 7.43E-01 | Physical measures |
| Body fat percentage | 1.01 | 1.50E-02 | 1.01 | 1.12E-02 | 1.00 | 4.52E-01 | 1.00 | 5.10E-01 | 1.01 | 3.13E-03 | 1.02 | 3.93E-04 | 1.01 | 1.22E-01 | 1.00 | 8.47E-01 | Physical measures |
| Basal metabolic rate | 1.00 | 2.14E-01 | 1.00 | 1.32E-01 | 1.00 | 9.73E-01 | 1.00 | 5.16E-01 | 1.00 | 1.30E-01 | 1.00 | 1.97E-01 | 1.00 | 1.09E-01 | 1.00 | 9.07E-01 | Physical measures |
| Trunk fat percentage | 1.01 | 1.30E-02 | 1.01 | 2.04E-02 | 1.00 | 2.78E-01 | 1.00 | 5.28E-01 | 1.01 | 2.03E-03 | 1.02 | 9.36E-04 | 1.01 | 1.24E-01 | 1.00 | 7.11E-01 | Physical measures |
| body mass index | 1.01 | 1.37E-01 | 1.01 | 4.69E-02 | 1.00 | 9.42E-01 | 1.00 | 9.78E-01 | 1.01 | 2.58E-02 | 1.02 | 4.46E-03 | 1.01 | 3.66E-02 | 1.00 | 4.19E-01 | Physical measures |
| Waist circumference | 1.01 | 4.25E-04 | 1.01 | 4.33E-04 | 1.00 | 2.06E-01 | 1.00 | 2.27E-02 | 1.01 | 5.48E-03 | 1.01 | 6.26E-05 | 1.01 | 7.98E-03 | 1.00 | 5.29E-01 | Physical measures |
| Hip circumference | 1.01 | 5.76E-03 | 1.01 | 4.40E-02 | 1.01 | 6.59E-02 | 1.00 | 2.53E-01 | 1.01 | 5.72E-03 | 1.01 | 7.88E-03 | 1.01 | 1.40E-02 | 1.00 | 5.83E-01 | Physical measures |
| NO2 | 1.00 | 8.83E-02 | 1.01 | 4.50E-02 | 1.00 | 6.59E-01 | 1.01 | 2.57E-02 | 1.00 | 8.83E-01 | 1.01 | 3.42E-02 | 1.01 | 8.00E-02 | 1.00 | 6.05E-01 | Local environment |
| NOx | 1.00 | 9.23E-01 | 1.00 | 1.26E-01 | 1.00 | 1.63E-01 | 1.00 | 6.86E-01 | 1.00 | 6.75E-01 | 1.00 | 2.72E-01 | 1.00 | 4.08E-01 | 1.00 | 2.38E-01 | Local environment |
| PM2.5 | 1.00 | 7.85E-01 | 1.03 | 1.53E-01 | 0.97 | 2.92E-01 | 1.01 | 6.11E-01 | 0.99 | 8.36E-01 | 1.04 | 3.16E-01 | 1.04 | 1.95E-01 | 0.97 | 2.37E-01 | Local environment |
| PM2.5absorbance | 0.98 | 7.52E-01 | 1.03 | 7.85E-01 | 0.93 | 4.89E-01 | 1.02 | 8.41E-01 | 0.93 | 4.98E-01 | 1.05 | 7.51E-01 | 1.08 | 5.36E-01 | 0.87 | 1.86E-01 | Local environment |
| PM2.5_10 | 0.99 | 5.35E-01 | 1.01 | 6.17E-01 | 0.96 | 1.37E-01 | 0.99 | 8.19E-01 | 0.98 | 6.34E-01 | 1.08 | 6.19E-02 | 0.95 | 1.41E-01 | 0.97 | 3.20E-01 | Local environment |
| PM10 | 1.01 | 2.67E-01 | 1.02 | 7.60E-02 | 1.00 | 8.12E-01 | 1.02 | 7.23E-02 | 1.00 | 8.53E-01 | 1.05 | 1.80E-02 | 1.01 | 3.87E-01 | 0.99 | 3.93E-01 | Local environment |
| Average daytime sound level of noise pollution | 1.00 | 4.49E-01 | 1.00 | 7.75E-01 | 1.00 | 4.32E-01 | 1.00 | 8.86E-01 | 0.99 | 2.82E-01 | 1.00 | 6.23E-01 | 0.99 | 4.72E-01 | 1.00 | 8.67E-01 | Local environment |
| Average evening sound level of noise pollution | 1.00 | 4.48E-01 | 1.00 | 7.74E-01 | 1.00 | 4.32E-01 | 1.00 | 8.84E-01 | 0.99 | 2.82E-01 | 1.00 | 6.23E-01 | 0.99 | 4.71E-01 | 1.00 | 8.67E-01 | Local environment |
| Average night-time sound level of noise pollution | 1.00 | 4.49E-01 | 1.00 | 7.75E-01 | 1.00 | 4.32E-01 | 1.00 | 8.86E-01 | 0.99 | 2.82E-01 | 1.00 | 6.23E-01 | 0.99 | 4.73E-01 | 1.00 | 8.67E-01 | Local environment |
| Average 16-hour sound level of noise pollution | 1.00 | 4.48E-01 | 1.00 | 7.75E-01 | 1.00 | 4.32E-01 | 1.00 | 8.86E-01 | 0.99 | 2.81E-01 | 1.00 | 6.23E-01 | 0.99 | 4.72E-01 | 1.00 | 8.66E-01 | Local environment |
| Average 24-hour sound level of noise pollution | 1.00 | 4.48E-01 | 1.00 | 7.75E-01 | 1.00 | 4.32E-01 | 1.00 | 8.85E-01 | 0.99 | 2.81E-01 | 1.00 | 6.23E-01 | 0.99 | 4.72E-01 | 1.00 | 8.67E-01 | Local environment |
| Greenspace percentage, buffer 1000m | 1.00 | 8.25E-02 | 1.00 | 4.62E-02 | 1.00 | 6.17E-01 | 1.00 | 5.34E-02 | 1.00 | 7.76E-01 | 1.00 | 7.67E-02 | 1.00 | 4.48E-02 | 1.00 | 6.46E-01 | Local environment |
| Domestic garden percentage, buffer 1000m | 1.00 | 9.46E-02 | 1.00 | 1.13E-01 | 1.00 | 4.34E-01 | 1.00 | 2.50E-02 | 1.00 | 8.07E-01 | 1.00 | 2.87E-01 | 1.01 | 5.54E-02 | 1.00 | 9.40E-01 | Local environment |
| Water percentage, buffer 1000m | 1.00 | 6.36E-01 | 1.00 | 8.08E-01 | 1.01 | 3.64E-01 | 1.00 | 7.09E-01 | 1.01 | 2.03E-01 | 1.00 | 8.57E-01 | 1.00 | 7.09E-01 | 1.00 | 7.48E-01 | Local environment |
| Greenspace percentage, buffer 300m | 1.00 | 2.69E-01 | 1.00 | 3.31E-02 | 1.00 | 5.48E-01 | 1.00 | 9.13E-02 | 1.00 | 6.60E-01 | 1.00 | 6.26E-01 | 1.00 | 8.02E-02 | 1.00 | 8.30E-01 | Local environment |
| Domestic garden percentage, buffer 300m | 1.00 | 4.64E-01 | 1.00 | 1.12E-01 | 1.00 | 5.28E-01 | 1.00 | 8.46E-02 | 1.00 | 2.63E-01 | 1.00 | 7.43E-01 | 1.00 | 1.25E-01 | 1.00 | 9.70E-01 | Local environment |
| Water percentage, buffer 300m | 1.01 | 3.92E-02 | 1.01 | 2.76E-01 | 1.01 | 6.64E-02 | 1.01 | 3.23E-01 | 1.02 | 3.49E-02 | 1.01 | 5.81E-01 | 1.01 | 3.97E-01 | 1.01 | 4.82E-02 | Local environment |
| Natural environment percentage, buffer 1000m | 1.00 | 1.34E-01 | 1.00 | 6.93E-02 | 1.00 | 7.21E-01 | 1.00 | 7.38E-02 | 1.00 | 9.35E-01 | 1.00 | 4.18E-02 | 1.00 | 2.35E-01 | 1.00 | 7.42E-01 | Local environment |
| Natural environment percentage, buffer 300m | 1.00 | 2.41E-01 | 1.00 | 5.26E-03 | 1.00 | 2.28E-01 | 1.00 | 7.44E-02 | 1.00 | 6.49E-01 | 1.00 | 3.18E-01 | 1.00 | 4.44E-01 | 1.00 | 7.42E-01 | Local environment |
| Distance (Euclidean) to coast | 1.00 | 3.92E-01 | 1.00 | 6.76E-01 | 1.00 | 4.33E-01 | 1.00 | 5.09E-01 | 1.00 | 5.94E-01 | 1.00 | 8.49E-01 | 1.00 | 3.11E-01 | 1.00 | 9.16E-01 | Local environment |
| Water hardness (USGS classification) | 1.06 | 1.19E-01 | 0.99 | 8.27E-01 | 1.13 | 1.42E-02 | 1.05 | 2.46E-01 | 1.06 | 3.03E-01 | 1.04 | 5.88E-01 | 1.08 | 2.50E-01 | 1.03 | 5.98E-01 | Local environment |
| Water hardness (WHO classification) | 1.08 | 2.36E-02 | 1.08 | 1.75E-01 | 1.17 | 1.91E-03 | 1.08 | 7.34E-02 | 1.08 | 1.75E-01 | 1.10 | 2.07E-01 | 1.11 | 1.06E-01 | 1.04 | 4.96E-01 | Local environment |
| CaCO3 concentration | 1.00 | 3.52E-02 | 1.00 | 7.82E-01 | 1.00 | 6.12E-03 | 1.00 | 8.22E-02 | 1.00 | 2.46E-01 | 1.00 | 2.97E-01 | 1.00 | 1.57E-01 | 1.00 | 4.67E-01 | Local environment |
| Ca concentration | 1.00 | 7.76E-04 | 1.00 | 1.27E-01 | 1.00 | 1.01E-03 | 1.00 | 1.10E-03 | 1.00 | 2.23E-01 | 1.00 | 4.47E-02 | 1.00 | 6.80E-02 | 1.00 | 1.28E-01 | Local environment |
| Mg concentration | 1.01 | 2.16E-01 | 1.00 | 6.58E-01 | 1.01 | 2.09E-01 | 1.00 | 4.69E-01 | 1.01 | 2.90E-01 | 1.01 | 2.30E-01 | 0.99 | 4.40E-01 | 1.01 | 1.25E-01 | Local environment |
| income 18,000 to 30,999 | 0.90 | 2.36E-02 | 0.80 | 1.29E-03 | 0.93 | 2.90E-01 | 0.90 | 6.29E-02 | 0.85 | 3.47E-02 | 0.80 | 1.88E-02 | 0.76 | 1.53E-03 | 1.01 | 8.42E-01 | Socioeconomic status |
| income 31,000 to 51,999 | 0.96 | 3.08E-01 | 0.86 | 1.19E-02 | 1.03 | 6.64E-01 | 0.94 | 2.61E-01 | 0.96 | 5.35E-01 | 0.89 | 1.95E-01 | 0.87 | 7.54E-02 | 1.02 | 7.28E-01 | Socioeconomic status |
| income 52,000 to 100,000 | 0.79 | 3.36E-04 | 0.70 | 1.11E-05 | 0.94 | 5.98E-01 | 0.81 | 7.54E-03 | 0.73 | 8.63E-03 | 0.57 | 1.97E-04 | 0.64 | 2.28E-04 | 1.00 | 9.60E-01 | Socioeconomic status |
| income Greater than 100,000 | 0.99 | 9.02E-01 | 0.92 | 4.86E-01 | 1.03 | 8.86E-01 | 1.00 | 9.80E-01 | 0.96 | 8.26E-01 | 0.62 | 7.82E-02 | 1.00 | 9.98E-01 | 1.11 | 4.66E-01 | Socioeconomic status |
| Lower education | 1.02 | 5.37E-01 | 1.05 | 3.31E-01 | 0.99 | 9.08E-01 | 0.99 | 7.71E-01 | 1.10 | 1.49E-01 | 1.27 | 3.15E-03 | 0.94 | 3.34E-01 | 1.01 | 8.32E-01 | Socioeconomic status |
| No employment | 1.51 | 1.17E-08 | 1.55 | 5.03E-09 | 0.96 | 9.11E-01 | 1.49 | 9.83E-06 | 1.56 | 2.96E-04 | 1.81 | 4.69E-05 | 1.25 | 1.13E-01 | 1.57 | 1.69E-05 | Socioeconomic status |
| Townsend deprivation index at recruitment | 1.00 | 4.33E-01 | 1.01 | 1.66E-01 | 1.00 | 8.60E-01 | 1.00 | 7.13E-01 | 1.01 | 4.16E-01 | 1.02 | 5.16E-02 | 1.01 | 1.46E-01 | 0.99 | 1.59E-01 | Socioeconomic status |
| Alcohol Score | 0.97 | 3.12E-05 | 0.96 | 3.93E-04 | 0.97 | 1.28E-02 | 0.97 | 2.63E-04 | 0.95 | 2.42E-02 | 0.97 | 8.57E-02 | 0.96 | 1.42E-02 | 0.96 | 2.11E-03 | Lifestyles |
| Vegetable score | 1.09 | 6.05E-01 | 0.96 | 8.51E-01 | 1.32 | 3.13E-01 | 1.27 | 2.11E-01 | 0.66 | 9.71E-02 | 1.03 | 9.18E-01 | 1.09 | 7.62E-01 | 1.07 | 7.75E-01 | Lifestyles |
| Fruit score | 0.95 | 4.72E-01 | 1.01 | 9.41E-01 | 0.87 | 2.14E-01 | 1.03 | 7.22E-01 | 0.75 | 4.20E-02 | 0.89 | 4.41E-01 | 0.91 | 4.84E-01 | 1.01 | 9.56E-01 | Lifestyles |
| Fish score | 0.86 | 3.37E-02 | 0.85 | 7.70E-02 | 0.87 | 2.17E-01 | 0.82 | 1.77E-02 | 0.97 | 8.24E-01 | 0.84 | 2.38E-01 | 0.77 | 2.17E-02 | 0.92 | 4.23E-01 | Lifestyles |
| Processedmeat score | 1.03 | 4.23E-01 | 0.96 | 4.00E-01 | 1.12 | 4.07E-02 | 1.05 | 2.97E-01 | 1.00 | 9.44E-01 | 1.05 | 5.23E-01 | 1.00 | 9.74E-01 | 1.02 | 6.97E-01 | Lifestyles |
| Unprocessedmeat score | 1.22 | 7.02E-03 | 1.20 | 5.57E-02 | 1.26 | 4.64E-02 | 1.23 | 3.78E-02 | 1.21 | 5.90E-02 | 1.29 | 1.03E-01 | 1.34 | 1.82E-02 | 1.09 | 4.25E-01 | Lifestyles |
| DietScore | 1.02 | 5.69E-01 | 0.98 | 5.40E-01 | 1.07 | 1.25E-01 | 1.03 | 3.66E-01 | 1.00 | 9.42E-01 | 1.02 | 7.45E-01 | 0.99 | 8.44E-01 | 1.01 | 7.34E-01 | Lifestyles |
| Number of days/week walked 10+ minutes | 0.99 | 3.44E-01 | 0.98 | 1.96E-01 | 1.00 | 9.17E-01 | 1.00 | 8.18E-01 | 0.98 | 1.87E-01 | 0.98 | 3.87E-01 | 0.99 | 7.35E-01 | 0.99 | 5.65E-01 | Lifestyles |
| Duration of walks | 1.00 | 1.97E-03 | 1.00 | 1.83E-02 | 1.00 | 4.28E-02 | 1.00 | 2.35E-04 | 1.00 | 9.56E-01 | 1.00 | 2.05E-01 | 1.00 | 1.96E-01 | 1.00 | 1.30E-02 | Lifestyles |
| Number of days/week of moderate physical activity 10+ minutes | 0.97 | 3.99E-05 | 0.96 | 3.63E-04 | 0.98 | 3.40E-02 | 0.97 | 7.58E-04 | 0.97 | 1.82E-02 | 0.98 | 1.27E-01 | 0.97 | 1.65E-02 | 0.97 | 2.60E-03 | Lifestyles |
| Number of days/week of vigorous physical activity 10+ minutes | 0.99 | 2.74E-01 | 0.98 | 5.56E-02 | 1.01 | 6.15E-01 | 1.00 | 7.89E-01 | 0.97 | 1.18E-01 | 1.00 | 8.15E-01 | 0.99 | 4.40E-01 | 0.99 | 3.92E-01 | Lifestyles |
| Usual walking pace Slow pace | 1.35 | 8.42E-09 | 1.51 | 3.92E-09 | 1.18 | 2.07E-02 | 1.32 | 1.84E-05 | 1.36 | 1.76E-04 | 1.62 | 5.86E-07 | 1.32 | 2.40E-03 | 1.21 | 1.33E-02 | Lifestyles |
| Usual walking pace Brisk pace | 0.80 | 1.53E-08 | 0.80 | 1.07E-05 | 0.78 | 2.69E-05 | 0.81 | 1.34E-05 | 0.76 | 1.32E-05 | 0.73 | 1.54E-04 | 0.74 | 1.68E-05 | 0.85 | 3.91E-03 | Lifestyles |
| Frequency of stair climbing in last 4 weeks | 0.99 | 8.12E-01 | 0.91 | 5.25E-02 | 1.09 | 1.05E-01 | 1.04 | 4.38E-01 | 0.92 | 1.57E-01 | 0.98 | 8.33E-01 | 0.94 | 3.85E-01 | 1.02 | 6.98E-01 | Lifestyles |
| Vitamin and mineral supplements | 0.97 | 3.62E-01 | 0.98 | 7.55E-01 | 0.95 | 3.11E-01 | 0.96 | 4.59E-01 | 0.97 | 5.92E-01 | 0.90 | 1.77E-01 | 0.93 | 2.72E-01 | 1.02 | 6.55E-01 | Lifestyles |
| Leisure/social activities | 0.96 | 2.59E-01 | 0.96 | 4.25E-01 | 0.96 | 4.31E-01 | 1.04 | 4.39E-01 | 0.83 | 3.40E-03 | 0.98 | 8.00E-01 | 0.90 | 1.22E-01 | 0.98 | 7.56E-01 | Lifestyles |
| Mineral and other dietary supplements | 0.96 | 3.09E-01 | 0.98 | 6.10E-01 | 0.95 | 2.96E-01 | 1.00 | 9.60E-01 | 0.90 | 7.84E-02 | 0.89 | 1.20E-01 | 0.93 | 2.76E-01 | 1.02 | 6.51E-01 | Lifestyles |
| Age first had sexual intercourse | 1.00 | 3.30E-01 | 0.99 | 1.38E-01 | 1.00 | 9.01E-01 | 1.00 | 5.12E-01 | 0.99 | 4.40E-01 | 0.99 | 1.97E-01 | 1.00 | 5.33E-01 | 1.00 | 8.82E-01 | Lifestyles |
| Lifetime number of sexual partners | 1.00 | 4.40E-01 | 1.00 | 6.22E-01 | 1.00 | 5.36E-01 | 1.00 | 4.46E-01 | 1.00 | 9.18E-01 | 1.00 | 4.45E-01 | 1.00 | 6.73E-01 | 1.00 | 1.03E-01 | Lifestyles |
| Ever had same-sex intercourse | 0.94 | 5.92E-01 | 0.92 | 5.85E-01 | 0.98 | 9.07E-01 | 0.96 | 7.71E-01 | 0.85 | 5.48E-01 | 0.63 | 1.24E-01 | 1.32 | 1.31E-01 | 0.84 | 3.33E-01 | Lifestyles |
| Time spend outdoors in summer | 0.97 | 2.17E-05 | 0.98 | 5.10E-02 | 0.95 | 2.20E-05 | 0.97 | 2.66E-03 | 0.95 | 9.70E-04 | 0.96 | 1.57E-02 | 0.98 | 1.11E-01 | 0.97 | 3.08E-03 | Lifestyles |
| Time spent outdoors in winter | 0.97 | 1.03E-02 | 0.99 | 5.48E-01 | 0.95 | 1.79E-03 | 0.98 | 9.86E-02 | 0.95 | 1.77E-02 | 0.96 | 5.68E-02 | 0.98 | 1.87E-01 | 0.98 | 2.58E-01 | Lifestyles |
| Time spent watching television (TV) | 1.04 | 1.48E-04 | 1.06 | 4.55E-06 | 1.01 | 4.62E-01 | 1.03 | 1.13E-02 | 1.05 | 2.88E-03 | 1.06 | 4.13E-03 | 1.05 | 1.21E-02 | 1.03 | 4.51E-02 | Lifestyles |
| Time spent using computer | 0.97 | 6.96E-02 | 0.98 | 3.66E-01 | 0.96 | 6.16E-02 | 0.96 | 1.67E-02 | 1.01 | 6.18E-01 | 0.95 | 8.71E-02 | 0.99 | 7.51E-01 | 0.97 | 1.63E-01 | Lifestyles |
| Time spent driving | 0.98 | 2.26E-01 | 0.97 | 1.47E-01 | 1.00 | 9.92E-01 | 0.99 | 4.40E-01 | 0.95 | 1.96E-01 | 1.02 | 6.09E-01 | 0.94 | 4.58E-02 | 0.99 | 8.09E-01 | Lifestyles |
| Weekly usage of mobile phone in last 3 months 5-29 mins | 1.10 | 3.18E-02 | 1.16 | 1.60E-02 | 1.03 | 5.96E-01 | 1.19 | 2.10E-03 | 0.95 | 4.92E-01 | 1.17 | 7.74E-02 | 0.96 | 6.16E-01 | 1.14 | 4.14E-02 | Lifestyles |
| 30-59 mins | 0.96 | 4.89E-01 | 1.04 | 6.20E-01 | 0.87 | 1.72E-01 | 1.05 | 5.52E-01 | 0.84 | 8.33E-02 | 1.07 | 6.08E-01 | 0.82 | 7.71E-02 | 1.02 | 8.51E-01 | Lifestyles |
| 1-3 hours | 0.89 | 1.18E-01 | 0.94 | 4.89E-01 | 0.81 | 9.89E-02 | 1.00 | 9.90E-01 | 0.68 | 2.99E-03 | 1.04 | 7.85E-01 | 0.77 | 5.73E-02 | 0.86 | 1.80E-01 | Lifestyles |
| 4-6 hours | 0.95 | 6.97E-01 | 1.00 | 9.88E-01 | 1.06 | 8.19E-01 | 1.12 | 4.63E-01 | 0.74 | 2.08E-01 | 0.91 | 7.39E-01 | 0.81 | 3.85E-01 | 1.12 | 5.26E-01 | Lifestyles |
| More than 6 hours | 0.79 | 1.26E-01 | 0.89 | 4.84E-01 | 0.47 | 9.43E-02 | 0.79 | 2.01E-01 | 0.77 | 3.14E-01 | 0.90 | 7.35E-01 | 0.72 | 2.17E-01 | 0.75 | 2.14E-01 | Lifestyles |
| Hands-free device/speakerphone use with mobile phone in last 3 month Less than half the time | 0.88 | 9.20E-02 | 0.92 | 3.57E-01 | 0.81 | 1.49E-01 | 0.90 | 2.29E-01 | 0.83 | 2.83E-01 | 0.89 | 4.58E-01 | 0.86 | 2.96E-01 | 0.89 | 2.77E-01 | Lifestyles |
| About half the time | 0.97 | 7.75E-01 | 1.01 | 9.64E-01 | 0.83 | 4.54E-01 | 1.00 | 9.70E-01 | 0.74 | 3.53E-01 | 1.01 | 9.82E-01 | 0.81 | 3.73E-01 | 1.01 | 9.49E-01 | Lifestyles |
| More than half the time | 0.57 | 6.83E-03 | 0.46 | 3.03E-03 | 1.11 | 7.34E-01 | 0.64 | 3.14E-02 | 0.48 | 2.10E-01 | 0.63 | 2.61E-01 | 0.45 | 5.02E-02 | 0.73 | 2.35E-01 | Lifestyles |
| Always or almost always | 1.04 | 7.75E-01 | 1.13 | 4.02E-01 | 0.81 | 4.13E-01 | 1.05 | 7.51E-01 | 1.00 | 9.93E-01 | 1.07 | 7.79E-01 | 1.34 | 1.50E-01 | 0.82 | 3.41E-01 | Lifestyles |
| Usual side of head for mobile phone use Left | 1.09 | 3.19E-01 | 1.07 | 5.14E-01 | 1.10 | 5.08E-01 | 1.09 | 4.39E-01 | 1.07 | 6.23E-01 | 1.23 | 2.70E-01 | 0.91 | 5.03E-01 | 1.17 | 2.15E-01 | Lifestyles |
| Right | 1.14 | 1.31E-01 | 1.12 | 2.76E-01 | 1.17 | 2.47E-01 | 1.17 | 1.35E-01 | 1.09 | 5.51E-01 | 1.23 | 2.50E-01 | 0.94 | 6.67E-01 | 1.26 | 6.25E-02 | Lifestyles |
| Sleep duration | 1.05 | 1.47E-03 | 1.02 | 4.11E-01 | 1.09 | 1.77E-04 | 1.04 | 3.72E-02 | 1.07 | 1.21E-02 | 1.00 | 9.02E-01 | 1.06 | 3.37E-02 | 1.07 | 2.34E-03 | Lifestyles |
| Getting up in morning Not very easy | 0.82 | 5.35E-02 | 0.74 | 1.31E-02 | 1.08 | 6.62E-01 | 0.82 | 1.61E-01 | 0.86 | 2.89E-01 | 0.87 | 5.12E-01 | 0.91 | 6.11E-01 | 0.78 | 9.06E-02 | Lifestyles |
| Getting up in morning Fairly easy | 0.69 | 9.92E-05 | 0.66 | 1.10E-04 | 0.89 | 4.82E-01 | 0.67 | 1.35E-03 | 0.80 | 9.19E-02 | 0.69 | 5.74E-02 | 0.81 | 2.25E-01 | 0.69 | 4.36E-03 | Lifestyles |
| Getting up in morning Very easy | 0.63 | 7.98E-07 | 0.56 | 2.01E-07 | 0.82 | 2.40E-01 | 0.61 | 8.09E-05 | 0.68 | 4.74E-03 | 0.68 | 5.03E-02 | 0.69 | 3.06E-02 | 0.61 | 1.51E-04 | Lifestyles |
| Nap during day Sometimes | 1.12 | 2.13E-03 | 1.13 | 1.26E-02 | 1.13 | 2.55E-02 | 1.09 | 4.85E-02 | 1.18 | 4.71E-03 | 1.16 | 4.50E-02 | 1.02 | 7.09E-01 | 1.18 | 1.31E-03 | Lifestyles |
| Nap during day Usually | 1.32 | 1.01E-05 | 1.40 | 1.31E-04 | 1.32 | 6.90E-04 | 1.29 | 2.26E-04 | 1.50 | 9.02E-04 | 1.32 | 2.87E-02 | 1.41 | 1.29E-03 | 1.33 | 1.76E-03 | Lifestyles |
| Sleeplessness / insomnia Sometimes | 0.95 | 2.53E-01 | 1.00 | 9.91E-01 | 0.91 | 1.05E-01 | 0.97 | 4.80E-01 | 0.93 | 3.80E-01 | 0.92 | 3.38E-01 | 0.95 | 4.83E-01 | 0.98 | 7.95E-01 | Lifestyles |
| Sleeplessness / insomnia Usually | 0.96 | 3.40E-01 | 1.08 | 2.36E-01 | 0.84 | 1.06E-02 | 0.97 | 5.79E-01 | 0.94 | 4.50E-01 | 1.03 | 7.72E-01 | 0.97 | 6.87E-01 | 0.92 | 2.52E-01 | Lifestyles |
| Snoring | 0.90 | 5.24E-03 | 0.88 | 6.77E-03 | 0.95 | 3.50E-01 | 0.96 | 2.92E-01 | 0.80 | 5.20E-04 | 0.89 | 1.10E-01 | 0.93 | 2.52E-01 | 0.89 | 2.42E-02 | Lifestyles |
| Daytime dozing / sleeping (narcolepsy) Sometimes | 1.14 | 6.04E-04 | 1.16 | 5.41E-03 | 1.14 | 1.22E-02 | 1.19 | 1.74E-04 | 1.07 | 2.74E-01 | 1.19 | 2.52E-02 | 1.10 | 1.77E-01 | 1.16 | 8.57E-03 | Lifestyles |
| Daytime dozing / sleeping (narcolepsy) Often/All of the time | 1.27 | 8.92E-03 | 1.34 | 1.60E-02 | 1.23 | 1.00E-01 | 1.35 | 3.72E-03 | 1.14 | 4.29E-01 | 1.47 | 2.35E-02 | 1.24 | 1.70E-01 | 1.21 | 1.55E-01 | Lifestyles |
| Exposure to tobacco smoke at home | 1.00 | 8.01E-01 | 1.00 | 7.06E-01 | 1.00 | 9.72E-01 | 1.00 | 6.13E-01 | 1.00 | 8.11E-01 | 1.00 | 9.00E-01 | 1.01 | 8.97E-02 | 0.99 | 4.22E-01 | Lifestyles |
| Exposure to tobacco smoke outside home | 1.01 | 3.26E-01 | 1.01 | 1.52E-01 | 1.00 | 9.17E-01 | 1.00 | 5.02E-01 | 1.01 | 3.95E-01 | 1.01 | 4.73E-01 | 1.01 | 4.87E-02 | 0.99 | 5.29E-01 | Lifestyles |
| Cheese intake | 0.98 | 3.74E-02 | 0.97 | 3.70E-02 | 0.99 | 3.77E-01 | 0.98 | 6.09E-02 | 0.98 | 3.35E-01 | 0.98 | 3.53E-01 | 0.98 | 2.42E-01 | 0.98 | 1.10E-01 | Lifestyles |
| Milk type used | 0.94 | 4.93E-01 | 0.90 | 4.13E-01 | 0.99 | 9.40E-01 | 0.90 | 3.63E-01 | 1.01 | 9.58E-01 | 1.19 | 4.43E-01 | 0.90 | 5.45E-01 | 0.87 | 3.06E-01 | Lifestyles |
| Spread type | 0.88 | 2.81E-02 | 0.88 | 7.79E-02 | 0.90 | 1.95E-01 | 0.89 | 1.20E-01 | 0.88 | 1.21E-01 | 0.85 | 1.73E-01 | 0.85 | 9.96E-02 | 0.95 | 5.04E-01 | Lifestyles |
| Bread intake | 1.00 | 3.16E-01 | 1.00 | 6.66E-01 | 1.00 | 3.28E-01 | 1.00 | 2.94E-01 | 1.00 | 8.76E-01 | 0.99 | 8.28E-02 | 1.00 | 8.22E-01 | 1.00 | 9.48E-01 | Lifestyles |
| Bread type Brown | 1.00 | 9.43E-01 | 0.92 | 3.13E-01 | 1.09 | 3.13E-01 | 1.01 | 8.62E-01 | 0.95 | 6.17E-01 | 0.85 | 2.24E-01 | 0.91 | 3.69E-01 | 1.13 | 1.55E-01 | Lifestyles |
| Wholemeal or wholegrain | 1.05 | 2.10E-01 | 1.03 | 6.22E-01 | 1.07 | 2.28E-01 | 1.12 | 2.39E-02 | 0.94 | 3.54E-01 | 1.22 | 1.78E-02 | 0.89 | 1.02E-01 | 1.08 | 1.88E-01 | Lifestyles |
| Other type of bread | 0.97 | 7.18E-01 | 0.87 | 2.85E-01 | 1.12 | 3.74E-01 | 0.91 | 4.95E-01 | 1.01 | 9.70E-01 | 0.93 | 7.25E-01 | 0.99 | 9.60E-01 | 1.00 | 9.84E-01 | Lifestyles |
| Cereal intake | 1.01 | 8.86E-02 | 1.01 | 1.90E-01 | 1.01 | 2.98E-01 | 1.01 | 1.58E-01 | 1.01 | 3.40E-01 | 1.02 | 1.36E-01 | 1.01 | 5.82E-01 | 1.01 | 2.90E-01 | Lifestyles |
| Biscuit cereal (e.g. Weetabix) | 0.93 | 2.80E-01 | 1.00 | 9.71E-01 | 0.84 | 4.66E-02 | 0.92 | 2.51E-01 | 0.91 | 3.88E-01 | 0.98 | 8.45E-01 | 0.82 | 7.96E-02 | 0.96 | 6.51E-01 | Lifestyles |
| Oat cereal (e.g. Ready Brek, porridge) | 0.90 | 5.45E-02 | 0.93 | 3.27E-01 | 0.82 | 9.41E-03 | 0.84 | 1.01E-02 | 0.94 | 4.33E-01 | 0.77 | 1.56E-02 | 0.82 | 3.70E-02 | 0.97 | 7.31E-01 | Lifestyles |
| Muesli | 1.00 | 9.84E-01 | 0.97 | 6.85E-01 | 0.99 | 9.41E-01 | 0.91 | 2.25E-01 | 1.10 | 3.24E-01 | 0.86 | 2.29E-01 | 0.94 | 5.54E-01 | 1.06 | 4.88E-01 | Lifestyles |
| Other (e.g. Cornflakes, Frosties) | 0.96 | 4.84E-01 | 0.98 | 7.75E-01 | 0.88 | 1.51E-01 | 0.89 | 1.23E-01 | 1.00 | 9.65E-01 | 0.80 | 7.58E-02 | 0.93 | 4.88E-01 | 1.01 | 9.31E-01 | Lifestyles |
| Salt added to food Sometimes | 0.92 | 4.91E-02 | 0.99 | 8.90E-01 | 0.85 | 5.46E-03 | 0.95 | 3.40E-01 | 0.88 | 3.94E-02 | 0.92 | 2.93E-01 | 0.89 | 9.43E-02 | 0.96 | 4.25E-01 | Lifestyles |
| Usually | 0.90 | 4.51E-02 | 0.96 | 5.33E-01 | 0.84 | 2.29E-02 | 0.95 | 4.26E-01 | 0.80 | 1.92E-02 | 0.86 | 1.74E-01 | 0.90 | 2.85E-01 | 0.92 | 3.09E-01 | Lifestyles |
| Always | 0.98 | 8.03E-01 | 1.07 | 5.41E-01 | 0.92 | 4.53E-01 | 1.06 | 5.11E-01 | 0.86 | 3.01E-01 | 1.13 | 4.17E-01 | 1.04 | 8.02E-01 | 0.90 | 3.80E-01 | Lifestyles |
| Tea intake | 1.00 | 9.82E-01 | 1.01 | 3.79E-01 | 0.99 | 3.62E-01 | 0.99 | 3.44E-01 | 1.01 | 1.55E-01 | 0.99 | 6.00E-01 | 1.00 | 8.43E-01 | 1.00 | 6.86E-01 | Lifestyles |
| Coffee intake | 1.01 | 2.91E-01 | 1.00 | 7.00E-01 | 1.01 | 2.93E-01 | 1.01 | 1.86E-01 | 1.00 | 9.57E-01 | 1.02 | 3.19E-01 | 1.00 | 9.25E-01 | 1.01 | 3.79E-01 | Lifestyles |
| Instant coffee | 0.88 | 4.85E-03 | 1.02 | 7.60E-01 | 0.75 | 6.42E-06 | 0.85 | 7.98E-03 | 0.91 | 1.57E-01 | 0.92 | 4.07E-01 | 0.88 | 1.13E-01 | 0.85 | 1.48E-02 | Lifestyles |
| Ground coffee (include espresso, filter etc) | 0.83 | 2.62E-03 | 0.93 | 3.83E-01 | 0.74 | 4.44E-04 | 0.85 | 2.44E-02 | 0.79 | 1.31E-02 | 0.69 | 3.99E-03 | 0.86 | 1.43E-01 | 0.86 | 8.18E-02 | Lifestyles |
| Other type of coffee | 0.82 | 2.37E-01 | 0.84 | 4.52E-01 | 0.71 | 1.45E-01 | 0.93 | 6.99E-01 | 0.56 | 4.34E-02 | 0.76 | 4.35E-01 | 0.63 | 1.53E-01 | 0.85 | 4.88E-01 | Lifestyles |
| Hot drink temperature Very hot | 1.04 | 8.70E-01 | 1.08 | 7.78E-01 | 0.90 | 7.49E-01 | 1.21 | 5.13E-01 | 0.80 | 4.73E-01 | 0.51 | 5.35E-02 | 1.36 | 4.63E-01 | 1.33 | 4.27E-01 | Lifestyles |
| Hot drink temperature Hot | 1.09 | 6.85E-01 | 1.10 | 7.19E-01 | 0.95 | 8.82E-01 | 1.25 | 4.42E-01 | 0.84 | 5.57E-01 | 0.58 | 1.04E-01 | 1.26 | 5.71E-01 | 1.43 | 3.14E-01 | Lifestyles |
| Hot drink temperature Warm | 1.14 | 5.53E-01 | 1.10 | 7.23E-01 | 1.03 | 9.41E-01 | 1.22 | 4.93E-01 | 0.95 | 8.67E-01 | 0.60 | 1.44E-01 | 1.35 | 4.73E-01 | 1.44 | 3.08E-01 | Lifestyles |
| Water intake | 1.01 | 1.82E-01 | 1.01 | 4.27E-01 | 1.02 | 1.97E-01 | 1.03 | 8.59E-03 | 0.98 | 2.52E-01 | 1.05 | 5.18E-03 | 1.00 | 8.97E-01 | 1.00 | 9.99E-01 | Lifestyles |
| Major dietary changes because of illness | 1.32 | 1.16E-08 | 1.41 | 9.52E-08 | 1.20 | 8.36E-03 | 1.30 | 3.99E-06 | 1.31 | 1.08E-03 | 1.58 | 7.75E-07 | 1.24 | 1.18E-02 | 1.22 | 5.15E-03 | Lifestyles |
| Major dietary changes because of other reasons | 0.98 | 5.97E-01 | 1.02 | 7.45E-01 | 0.93 | 2.22E-01 | 0.95 | 3.14E-01 | 1.02 | 7.41E-01 | 1.05 | 6.02E-01 | 0.85 | 3.62E-02 | 1.04 | 5.08E-01 | Lifestyles |
| Plays computer games Sometimes | 0.89 | 1.82E-02 | 0.93 | 2.32E-01 | 0.86 | 4.01E-02 | 0.90 | 7.77E-02 | 0.89 | 1.53E-01 | 0.90 | 2.87E-01 | 0.90 | 2.30E-01 | 0.91 | 1.57E-01 | Lifestyles |
| Plays computer games Often | 0.86 | 1.42E-01 | 0.89 | 4.00E-01 | 0.84 | 2.39E-01 | 0.92 | 5.32E-01 | 0.78 | 1.39E-01 | 1.12 | 5.45E-01 | 1.00 | 9.97E-01 | 0.67 | 1.77E-02 | Lifestyles |
| Use of sun/uv protection Never/rarely | 0.81 | 3.11E-01 | 1.17 | 6.28E-01 | 0.64 | 9.04E-02 | 0.88 | 6.55E-01 | 0.90 | 7.09E-01 | 0.65 | 2.72E-01 | 1.53 | 3.46E-01 | 0.69 | 1.81E-01 | Lifestyles |
| Use of sun/uv protection Sometimes | 0.82 | 3.32E-01 | 1.10 | 7.59E-01 | 0.69 | 1.48E-01 | 0.91 | 7.28E-01 | 0.81 | 4.45E-01 | 0.74 | 4.31E-01 | 1.29 | 5.68E-01 | 0.75 | 2.86E-01 | Lifestyles |
| Use of sun/uv protection Most of the time | 0.79 | 2.44E-01 | 1.14 | 6.75E-01 | 0.63 | 6.48E-02 | 0.90 | 6.99E-01 | 0.76 | 3.31E-01 | 0.70 | 3.56E-01 | 1.35 | 5.02E-01 | 0.71 | 1.98E-01 | Lifestyles |
| Use of sun/uv protection Always | 0.79 | 2.40E-01 | 1.09 | 7.95E-01 | 0.65 | 9.09E-02 | 0.94 | 8.28E-01 | 0.72 | 2.36E-01 | 0.76 | 4.85E-01 | 1.23 | 6.46E-01 | 0.71 | 2.03E-01 | Lifestyles |
| Frequency of solarium/sunlamp use | 0.99 | 1.27E-01 | 0.99 | 3.05E-01 | 0.98 | 2.56E-01 | 0.99 | 2.71E-01 | 0.98 | 2.79E-01 | 0.89 | 3.67E-02 | 0.99 | 4.51E-01 | 1.00 | 8.12E-01 | Lifestyles |
| Previous Smoking | 0.90 | 4.66E-03 | 0.89 | 1.72E-02 | 0.89 | 2.03E-02 | 0.89 | 9.27E-03 | 0.90 | 6.79E-02 | 0.83 | 1.19E-02 | 0.94 | 3.61E-01 | 0.90 | 4.73E-02 | Lifestyles |
| Current Smoking | 0.66 | 9.80E-09 | 0.72 | 1.71E-04 | 0.54 | 3.00E-07 | 0.62 | 3.20E-08 | 0.72 | 7.27E-03 | 0.58 | 2.90E-04 | 0.68 | 2.36E-03 | 0.68 | 1.39E-04 | Lifestyles |
| Previous alcohol | 1.18 | 1.28E-01 | 1.18 | 2.29E-01 | 1.17 | 2.87E-01 | 1.45 | 1.66E-02 | 1.08 | 5.81E-01 | 1.11 | 6.22E-01 | 1.19 | 3.24E-01 | 1.27 | 1.12E-01 | Lifestyles |
| Current alcohol | 0.75 | 2.38E-04 | 0.68 | 2.23E-04 | 0.83 | 9.34E-02 | 0.94 | 6.16E-01 | 0.66 | 1.52E-05 | 0.73 | 4.75E-02 | 0.70 | 7.55E-03 | 0.83 | 1.06E-01 | Lifestyles |
| IPAQ activity group high | 0.94 | 8.92E-02 | 0.93 | 1.35E-01 | 0.97 | 5.46E-01 | 0.93 | 1.20E-01 | 0.97 | 6.13E-01 | 0.95 | 5.26E-01 | 1.01 | 8.46E-01 | 0.90 | 6.14E-02 | Lifestyles |
| IPAQ activity group low | 1.06 | 2.66E-01 | 1.11 | 1.47E-01 | 1.00 | 9.56E-01 | 1.07 | 3.08E-01 | 1.05 | 6.29E-01 | 0.97 | 8.20E-01 | 0.99 | 9.55E-01 | 1.16 | 5.26E-02 | Lifestyles |
| At or above moderate/vigorous/walking recommendation | 0.90 | 2.88E-02 | 0.85 | 1.09E-02 | 0.98 | 7.66E-01 | 0.90 | 8.76E-02 | 0.89 | 1.68E-01 | 0.96 | 7.22E-01 | 0.99 | 9.26E-01 | 0.81 | 3.34E-03 | Lifestyles |
| MET minutes per week for walking | 1.00 | 4.69E-04 | 1.00 | 6.72E-03 | 1.00 | 2.69E-02 | 1.00 | 3.66E-04 | 1.00 | 3.13E-01 | 1.00 | 2.76E-02 | 1.00 | 1.61E-01 | 1.00 | 1.83E-02 | Lifestyles |
| MET minutes per week for moderate activity | 1.00 | 1.23E-06 | 1.00 | 1.98E-03 | 1.00 | 1.81E-04 | 1.00 | 3.72E-06 | 1.00 | 6.48E-02 | 1.00 | 7.96E-03 | 1.00 | 1.86E-02 | 1.00 | 7.62E-04 | Lifestyles |
| MET minutes per week for vigorous activity | 1.00 | 3.19E-03 | 1.00 | 6.94E-03 | 1.00 | 1.64E-01 | 1.00 | 2.83E-02 | 1.00 | 3.44E-02 | 1.00 | 3.06E-01 | 1.00 | 8.07E-02 | 1.00 | 3.00E-02 | Lifestyles |
| Summed MET minutes per week for all activity | 1.00 | 1.31E-06 | 1.00 | 3.15E-04 | 1.00 | 1.40E-03 | 1.00 | 1.20E-05 | 1.00 | 3.28E-02 | 1.00 | 1.47E-02 | 1.00 | 2.00E-02 | 1.00 | 5.39E-04 | Lifestyles |

**Supplementary Table5 GWAS ID in MRC IEU OpenGWAS database for exposures**

| ID exposure | Trait |
| --- | --- |
| ukb-b-13764 | Long-standing illness, disability or infirmity |
| ukb-b-2535 | Falls in the last year |
| ukb-b-10591 | Chest pain or discomfort |
| ukb-b-3855 | Attendance/disability/mobility allowance: None of the above |
| ukb-b-20214 | Attendance/disability/mobility allowance: Disability living allowance |
| ukb-b-16671 | Attendance/disability/mobility allowance: Attendance allowance |
| ukb-b-1867 | Attendance/disability/mobility allowance: Blue badge |
| ukb-e-E11_AFR | E11 Non-insulin-dependent diabetes mellitus |
| finn-b-E4_DIABETES | Diabetes mellitus |
| finn-b-AB1_VARICELLA | Varicella [chickenpox] |
| finn-b-AB1_MEASLES | Measles |
| finn-b-AB1_MUMPS | Mumps |
| finn-b-E4_LIPOPROT | Disorders of lipoprotein metabolism and other lipidaemias |
| ukb-b-15686 | Diagnoses - secondary ICD10: I20.9 Angina pectoris, unspecified |
| ukb-b-8184 | Diagnoses - secondary ICD10: I25.9 Chronic ischaemic heart disease, unspecified |
| ukb-b-8714 | Vascular/heart problems diagnosed by doctor: Stroke |
| ukb-b-18994 | Miserableness |
| ukb-b-19809 | Fed-up feelings |
| ukb-b-20544 | Nervous feelings |
| ukb-b-6519 | Worrier / anxious feelings |
| ukb-b-10093 | Tense / 'highly strung' |
| ukb-b-19957 | Suffer from 'nerves' |
| ukb-b-8476 | Loneliness, isolation |
| ukb-b-10169 | Guilty feelings |
| ukb-b-3822 | Frequency of depressed mood in last 2 weeks |
| ukb-b-1419 | Frequency of unenthusiasm / disinterest in last 2 weeks |
| ukb-b-5664 | Frequency of tenseness / restlessness in last 2 weeks |
| ukb-b-929 | Frequency of tiredness / lethargy in last 2 weeks |
| ukb-b-6991 | Seen doctor (GP) for nerves, anxiety, tension or depression |
| ukb-b-18336 | Seen a psychiatrist for nerves, anxiety, tension or depression |
| ukb-b-20188 | Arm fat percentage (left) |
| ukb-b-12854 | Arm fat percentage (right) |
| ukb-b-7478 | Hand grip strength (left) |
| ukb-b-10215 | Hand grip strength (right) |
| ukb-b-6740 | Current employment status: In paid employment or self-employed |
| ukb-b-20437 | Current employment status: Full or part-time student |
| ukb-b-12885 | Current employment status: Unemployed |
| ukb-b-6748 | Current employment status: None of the above |
| ukb-b-19852 | Current employment status: Retired |
| ukb-b-9056 | Current employment status: Looking after home and/or family |
| ukb-b-8810 | Current employment status: Doing unpaid or voluntary work |
| ukb-b-14080 | Current employment status: Unable to work because of sickness or disability |
| ukb-b-13978 | Average monthly intake of other alcoholic drinks |
| ukb-b-217 | Average monthly champagne plus white wine intake |
| ukb-b-2603 | Average monthly fortified wine intake |
| ukb-b-17300 | Average monthly red wine intake |
| ukb-b-20321 | Average monthly beer plus cider intake |
| ukb-b-10830 | Average monthly spirits intake |
| ukb-b-1707 | Average weekly spirits intake |
| ukb-b-1070 | Average weekly fortified wine intake |
| ukb-b-3831 | Average weekly intake of other alcoholic drinks |
| ukb-b-5174 | Average weekly beer plus cider intake |
| ukb-b-5716 | Average weekly champagne plus white wine intake |
| ukb-b-5239 | Average weekly red wine intake |
| ukb-b-4710 | Number of days/week of moderate physical activity 10+ minutes |
| ukb-b-4711 | Usual walking pace |
| ukb-b-969 | Time spend outdoors in summer |
| ukb-b-2772 | Getting up in morning |
| ukb-b-4616 | Nap during day |
| ukb-d-1538_1 | Major dietary changes in the last 5 years: Yes, because of illness |
| ukb-a-225 | Smoking status: Current |
| ukb-b-2115 | Time spent doing moderate physical activity |
| ukb-b-8865 | Time spent doing light physical activity |
| ukb-b-13702 | Time spent doing vigorous physical activity |

**Supplementary Table6 Two-sample mendelian randomization analyses identified risk factors for Parkinson's disease**

| Trait | Inverse variance weighted | | | | MR Egger | | | | Weighted median | | | | Heterogeity | | Horizontal Pleiotropy | | |
| --- | --- | --- | --- | --- | --- | --- | --- | --- | --- | --- | --- | --- | --- | --- | --- | --- | --- |
|  | OR | LCI | UCI | p | OR | LCI | UCI | p | OR | LCI | UCI | p | Q | Q.pval | P.Egger.intercept | P.MR.PRESSO.global.test | Pval_distortion test |
| Smoking status: Current | 0.142495192 | 0.044921 | 0.4520125 | 0.000939 | 0.033185 | 0.0006178 | 1.782549657 | 0.097327 | 0.0592595 | 0.011667 | 0.300993694 | 0.000654 | 98.01197 | 0.264451 | 0.455674 | 0.236 | NA |
| Arm fat percentage (right) | 0.751516779 | 0.629848 | 0.8966879 | 0.001524 | 0.6623451 | 0.400796 | 1.094574507 | 0.108868 | 0.8713428 | 0.6527866 | 1.163072688 | 0.349937 | 381.6634 | 0.117518 | 0.598869 | 0.067 | NA |
| Arm fat percentage (left) | 0.763232969 | 0.641846 | 0.9075771 | 0.002233 | 0.7190975 | 0.4384175 | 1.179472074 | 0.19236 | 0.8713044 | 0.6509486 | 1.166253717 | 0.354389 | 368.006 | 0.220823 | 0.801247 | 0.143 | NA |
| Usual walking pace | 2.795036762 | 1.415724 | 5.5181889 | 0.003059 | 0.7465669 | 0.0501518 | 11.11351421 | 0.832833 | 1.9805873 | 0.7781219 | 5.041274768 | 0.15166 | 63.7115 | 0.148858 | 0.326716 | 0.169 | NA |
| E11 Non-insulin-dependent diabetes mellitus | 1.061079295 | 1.018351 | 1.1056005 | 0.004696 | 1.0692581 | 1.0048832 | 1.137756909 | 0.168865 | 1.0613478 | 1.0091215 | 1.116277138 | 0.020739 | 0.173218 | 0.981792 | 0.777108 | 0.944 | NA |
| Seen doctor (GP) for nerves, anxiety, tension or depression | 4.756238004 | 1.56667 | 14.439418 | 0.005916 | 5.3592845 | 0.0105765 | 2715.644161 | 0.600795 | 3.5579831 | 0.6848771 | 18.48396439 | 0.131109 | 35.51299 | 0.396859 | 0.969759 | 0.461 | NA |
| Long-standing illness, disability or infirmity | 5.351525723 | 1.388307 | 20.628592 | 0.014827 | 136.65926 | 0.0532354 | 350814.8585 | 0.233135 | 2.3379189 | 0.3651861 | 14.96733979 | 0.369957 | 15.9699 | 0.817377 | 0.420779 | 0.812 | NA |
| Time spent doing moderate physical activity | 0.64980565 | 0.449384 | 0.9396139 | 0.021962 | 0.6160311 | 0.3105915 | 1.221843984 | 0.214901 | 0.6565902 | 0.4053466 | 1.063560812 | 0.087343 | 3.827692 | 0.799396 | 0.862101 | 0.815 | NA |
| Tense / 'highly strung' | 5.570926333 | 1.271963 | 24.399471 | 0.022654 | 2930.8798 | 0.0457774 | 187648251.3 | 0.167379 | 14.084496 | 1.7839697 | 111.1975262 | 0.012105 | 36.64804 | 0.261888 | 0.271496 | 0.34 | NA |
| Falls in the last year | 3.235357556 | 1.171441 | 8.9356062 | 0.023495 | 5.9024029 | 0.1060558 | 328.4909035 | 0.391008 | 2.8842153 | 0.7377926 | 11.27511712 | 0.127803 | 31.58002 | 0.967636 | 0.763201 | 0.969 | NA |
| Guilty feelings | 8.12883501 | 1.321015 | 50.020606 | 0.023802 | 0.0173825 | 2.36E-10 | 1280149.945 | 0.666578 | 3.3186277 | 0.3065869 | 35.92225095 | 0.323585 | 22.92616 | 0.193444 | 0.512694 | 0.181 | NA |
| Worrier / anxious feelings | 2.688829119 | 1.11368 | 6.4918136 | 0.027848 | 0.4291751 | 0.0032123 | 57.33960525 | 0.736118 | 4.2916652 | 1.3514852 | 13.62825874 | 0.013476 | 72.99222 | 0.06318 | 0.4582 | 0.052 | NA |
| Frequency of unenthusiasm / disinterest in last 2 weeks | 4.909749355 | 1.093979 | 22.034823 | 0.037778 | 33.60949 | 0.1883665 | 5996.807761 | 0.220546 | 5.3540825 | 0.7337379 | 39.06871642 | 0.09799 | 6.288273 | 0.710756 | 0.469159 | 0.729 | NA |
| Getting up in morning | 0.598261498 | 0.368303 | 0.9717988 | 0.037933 | 0.6201609 | 0.1255804 | 3.062576832 | 0.559727 | 0.8888325 | 0.4432089 | 1.782507736 | 0.739943 | 73.04924 | 0.205172 | 0.96318 | 0.204 | NA |
| Nervous feelings | 3.090717833 | 1.01479 | 9.4133095 | 0.047051 | 0.0386949 | 0.0001445 | 10.36074914 | 0.260648 | 2.4015986 | 0.5286589 | 10.91001247 | 0.256556 | 54.91038 | 0.105231 | 0.124796 | 0.127 | NA |
| Current employment status: Unemployed | 27266.44746 | 0.378972 | 19617803 | 0.073462 | 1669.7425 | 1.19E-33 | 2.33E+39 | 0.866223 | 1135.5106 | 0.0014852 | 868130775.5 | 0.308768 | 1.77994 | 0.987031 | 0.948934 | 0.987 | NA |
| Seen a psychiatrist for nerves, anxiety, tension or depression | 4.008957044 | 0.82572 | 19.463895 | 0.084988 | 0.9981656 | 0.000984 | 1012.558123 | 0.999587 | 1.4183867 | 0.1495326 | 13.45406563 | 0.760745 | 48.19137 | 0.817248 | 0.687461 | 0.875 | NA |
| Attendance/disability/mobility allowance: Disability living allowance | 0.125715208 | 0.008307 | 1.9024698 | 0.134648 | 0.000717 | 6.32E-08 | 8.129193123 | 0.134436 | 0.0863188 | 0.0020555 | 3.624913836 | 0.198915 | 60.37655 | 0.256372 | 0.262104 | 0.322 | NA |
| Time spend outdoors in summer | 0.613409327 | 0.318794 | 1.1802949 | 0.143308 | 0.6002121 | 0.0148218 | 24.30574455 | 0.7883 | 0.6375334 | 0.2922676 | 1.390673519 | 0.257956 | 73.59054 | 0.001334 | 0.990718 | <0.001 | 0.989 |
| Diagnoses - secondary ICD10: I25.9 Chronic ischaemic heart disease, unspecified | 788.6495913 | 0.070936 | 8768014.7 | 0.160519 | 0.0001031 | 4.51E-17 | 235760925.6 | 0.541417 | 3.5043109 | 2.92E-05 | 420470.2063 | 0.833544 | 13.50383 | 0.261674 | 0.275612 | 0.376 | NA |
| Current employment status: Unable to work because of sickness or disability | 16.99160627 | 0.314434 | 918.20502 | 0.164039 | 177.94015 | 1.75E-06 | 18104909043 | 0.585719 | 16.930639 | 0.0992513 | 2888.086962 | 0.2806 | 41.83273 | 0.114418 | 0.799701 | 0.17 | NA |
| Current employment status: Doing unpaid or voluntary work | 136.2728618 | 0.102957 | 180369.21 | 0.180215 | 28.488851 | 1.82E-10 | 4.45E+12 | 0.803266 | 91.451464 | 0.0175498 | 476549.7332 | 0.301056 | 21.05643 | 0.07182 | 0.903087 | 0.085 | NA |
| Varicella [chickenpox] | 0.959473055 | 0.902126 | 1.0204657 | 0.188273 | 0.9326246 | 0.824663 | 1.054720122 | 0.347508 | 0.9649165 | 0.8935096 | 1.042030061 | 0.362589 | 1.492596 | 0.827952 | 0.637495 | 0.531 | NA |
| Average monthly spirits intake | 1.365960605 | 0.853331 | 2.1865466 | 0.193866 | 2.9622025 | 0.7651632 | 11.4676757 | 0.146928 | 1.4518121 | 0.7778448 | 2.709741568 | 0.241624 | 13.01702 | 0.292215 | 0.260646 | 0.343 | NA |
| Average weekly beer plus cider intake | 0.617470324 | 0.298322 | 1.27804511 | 0.193945 | 0.3248989 | 0.0385616 | 2.737419656 | 0.315666 | 0.5964301 | 0.2091931 | 1.700480785 | 0.333647 | 15.99921 | 0.592602 | 0.53819 | 0.413 | NA |
| Average weekly fortified wine intake | 3.16905407 | 0.546098 | 18.390308 | 0.198555 | 0.8524977 | 0.0061256 | 118.6426549 | 0.950132 | 1.7061031 | 0.1743164 | 16.69830205 | 0.646224 | 22.56751 | 0.310526 | 0.582377 | 0.35 | NA |
| Average weekly champagne plus white wine intake | 1.501853191 | 0.806577 | 2.7964633 | 0.199747 | 0.551262 | 0.0636468 | 4.774628845 | 0.59145 | 1.6918332 | 0.6934852 | 4.127412257 | 0.247852 | 50.02237 | 0.280749 | 0.34717 | 0.291 | NA |
| Time spent doing light physical activity | 0.997596444 | 0.993883 | 1.0013238 | 0.205974 | 0.9941052 | 0.9863338 | 1.001937931 | 0.15097 | 1.0025272 | 0.997206 | 1.007876866 | 0.35259 | 27.43442 | 0.54828 | 0.32824 | 0.525 | NA |
| Average weekly intake of other alcoholic drinks | 4.520974123 | 0.429233 | 47.617986 | 0.209135 | 11.634917 | 0.1309525 | 1033.743467 | 0.295352 | 4.1397726 | 0.152379 | 112.4677329 | 0.399085 | 16.64815 | 0.826153 | 0.632421 | 0.822 | NA |
| Mumps | 1.027160693 | 0.983332 | 1.0729427 | 0.228392 | 1.0199599 | 0.9530687 | 1.091545875 | 0.607933 | 1.0336558 | 0.9780356 | 1.092439123 | 0.240797 | 1.484324 | 0.829415 | 0.807904 | 0.88 | NA |
| Measles | 0.985434482 | 0.961528 | 1.0099357 | 0.241609 | 0.9872484 | 0.9567423 | 1.018727279 | 0.481504 | 0.9834076 | 0.9546294 | 1.01305335 | 0.269526 | 1.785211 | 0.775187 | 0.865447 | 0.506 | NA |
| Miserableness | 0.558925662 | 0.208522 | 1.4981566 | 0.247506 | 0.1170899 | 0.0020126 | 6.812181048 | 0.307604 | 0.6556165 | 0.1616489 | 2.659053874 | 0.554529 | 32.45161 | 0.723351 | 0.442007 | 0.734 | NA |
| Major dietary changes in the last 5 years: Yes, because of illness | 0.20046647 | 0.011826 | 3.3982378 | 0.265749 | 0.1017555 | 1.48E-06 | 7009.15938 | 0.708201 | 0.248484 | 0.0084812 | 7.280106977 | 0.419087 | 1.209658 | 0.943947 | 0.907781 | 0.95 | NA |
| Hand grip strength (right) | 0.832546796 | 0.60105 | 1.1532062 | 0.270253 | 1.1748196 | 0.3534561 | 3.90487262 | 0.793046 | 0.883136 | 0.538206 | 1.449127609 | 0.622828 | 102.2364 | 0.954545 | 0.560351 | 0.936 | NA |
| Fed-up feelings | 0.613235184 | 0.25313 | 1.4856269 | 0.278724 | 0.3507881 | 0.0048135 | 25.56403122 | 0.634435 | 0.804686 | 0.2375928 | 2.725333333 | 0.726984 | 36.84129 | 0.830463 | 0.795373 | 0.884 | NA |
| Average monthly beer plus cider intake | 1.385004733 | 0.762574 | 2.5154768 | 0.284735 | 1.7641295 | 0.3544411 | 8.780452243 | 0.519019 | 1.593585 | 0.7548205 | 3.364393168 | 0.221617 | 2.018067 | 0.918029 | 0.76311 | 0.922 | NA |
| Attendance/disability/mobility allowance: Blue badge | 8.891770389 | 0.153797 | 514.07756 | 0.291149 | 1059.344 | 4.99E-05 | 2.25E+10 | 0.424014 | 3.4830439 | 0.0165601 | 732.5783799 | 0.647462 | 44.5404 | 0.129513 | 0.570752 | 0.132 | NA |
| Current employment status: Full or part-time student | 2.08E-04 | 1.97E-11 | 2200.2382 | 0.30426 | 1.38E+30 | 1.31E-11 | 1.45E+71 | 0.193059 | 2.43E-01 | 7.26E-11 | 809940658.9 | 0.899252 | 8.312166 | 0.403586 | 0.145186 | 0.422 | NA |
| Frequency of depressed mood in last 2 weeks | 2.055344075 | 0.518193 | 8.1522428 | 0.305441 | 67.688035 | 0.0008018 | 5714482.064 | 0.483152 | 1.7498405 | 0.285944 | 10.70818802 | 0.544915 | 5.834061 | 0.884203 | 0.556556 | 0.886 | NA |
| Attendance/disability/mobility allowance: Attendance allowance | 792.9673479 | 0.001563 | 4.02E+08 | 0.319247 | 5.8344279 | 1.88E-38 | 1.81E+39 | 0.969585 | 1221.3121 | 1.67E-05 | 8.93E+10 | 0.44168 | 11.71007 | 0.469237 | 0.914455 | 0.472 | NA |
| Time spent doing vigorous physical activity | 1.199720827 | 0.819593 | 1.7561529 | 0.348944 | 1.6527748 | 0.5878071 | 4.6472128 | 0.365673 | 1.0801233 | 0.6570446 | 1.775627478 | 0.761196 | 4.734406 | 0.908199 | 0.529847 | 0.908 | NA |
| Vascular/heart problems diagnosed by doctor: Stroke | 133.3984251 | 0.003971 | 4481084.8 | 0.357439 | 994534.15 | 1.49E-19 | 6.62E+30 | 0.64847 | 666.75323 | 0.000758 | 586458684.9 | 0.351779 | 7.364478 | 0.599228 | 0.76377 | 0.62 | NA |
| Current employment status: None of the above | 186858942.3 | 3.91E-10 | 8.94E+25 | 0.359144 | 3.04E-69 | 0 | 6.87E+222 | 0.725039 | 1.25E+12 | 1.49E-10 | 1.06E+34 | 0.279486 | 0.48308 | 0.785417 | 0.695968 | 0.812 | NA |
| Frequency of tenseness / restlessness in last 2 weeks | 2.105300534 | 0.424712 | 10.435996 | 0.36203 | 0.0970261 | 0.0004922 | 19.12800623 | 0.415536 | 1.2271522 | 0.1291999 | 11.65560351 | 0.858545 | 7.433323 | 0.490681 | 0.269994 | 0.48 | NA |
| Average monthly fortified wine intake | 0.836971624 | 0.558704 | 1.2538327 | 0.38812 | 0.6131454 | 0.2574526 | 1.460258443 | 0.280655 | 0.8175149 | 0.4759508 | 1.404201074 | 0.465372 | 27.73715 | 0.271282 | 0.434174 | 0.275 | NA |
| Average monthly intake of other alcoholic drinks | 1.172683318 | 0.814378 | 1.6886335 | 0.391849 | 1.9754287 | 0.950897 | 4.103828623 | 0.078314 | 0.9012565 | 0.5299573 | 1.532695838 | 0.701159 | 23.76887 | 0.78238 | 0.117601 | 0.741 | NA |
| Average weekly red wine intake | 1.358796445 | 0.651976 | 2.8318952 | 0.413171 | 1.0688695 | 0.0223685 | 51.07558996 | 0.973582 | 1.3371287 | 0.5011415 | 3.567681578 | 0.561763 | 10.07352 | 0.756788 | 0.903284 | 0.591 | NA |
| Frequency of tiredness / lethargy in last 2 weeks | 1.33121332 | 0.665263 | 2.6638036 | 0.418877 | 0.5708827 | 0.0199783 | 16.31304923 | 0.745959 | 1.325318 | 0.5111553 | 3.436270383 | 0.562302 | 19.25957 | 0.784508 | 0.617519 | 0.435 | NA |
| Attendance/disability/mobility allowance: None of the above | 2.326376129 | 0.25512 | 21.213632 | 0.454045 | 2.1907886 | 0.0006694 | 7170.233775 | 0.850079 | 7.4956379 | 0.2794774 | 201.0344812 | 0.230011 | 49.66455 | 0.677965 | 0.987994 | 0.756 | NA |
| Diagnoses - secondary ICD10: I20.9 Angina pectoris, unspecified | 5.291689704 | 5.50E-02 | 508.81372 | 0.474476 | 0.4968806 | 4.75E-06 | 5.20E+04 | 0.908191 | 3.9835511 | 1.47E-02 | 1081.730651 | 0.62881 | 3.598287 | 0.963655 | 0.672631 | 0.98 | NA |
| Nap during day | 1.208275032 | 0.716587 | 2.0373352 | 0.477845 | 0.7131622 | 0.1144672 | 4.443195356 | 0.718213 | 1.0673687 | 0.4979782 | 2.287802587 | 0.86689 | 71.80987 | 0.675658 | 0.557305 | 0.686 | NA |
| Disorders of lipoprotein metabolism and other lipidaemias | 0.972959454 | 0.899818 | 1.0520466 | 0.491761 | 0.9411465 | 0.8060464 | 1.098890429 | 0.455695 | 0.9743746 | 0.8751448 | 1.084855747 | 0.6357 | 5.385467 | 0.988329 | 0.633829 | 0.987 | NA |
| Suffer from 'nerves' | 1.749850126 | 0.351918 | 8.7008291 | 0.494125 | 0.0023493 | 1.25E-07 | 44.1862866 | 0.243599 | 2.548172 | 0.2727892 | 23.80292374 | 0.411935 | 15.58997 | 0.684436 | 0.198573 | 0.672 | NA |
| Current employment status: Retired | 0.553459 | 0.100733 | 3.0408730 | 0.496152 | 1.0374694 | 0.0179808 | 59.86051565 | 0.985975 | 1.1332568 | 0.1080349 | 11.88755528 | 0.916917 | 12.81158 | 0.955958 | 0.741046 | 0.964 | NA |
| Current employment status: In paid employment or self-employed | 0.565988013 | 0.10932 | 2.9303309 | 0.497477 | 1.1629743 | 0.0109513 | 123.5017129 | 0.949949 | 0.3852933 | 0.0379312 | 3.913693621 | 0.42003 | 19.44893 | 0.775102 | 0.749264 | 0.77 | NA |
| Average monthly red wine intake | 1.415494141 | 0.515892 | 3.8838078 | 0.49983 | 204.43673 | 2.3730281 | 17612.25503 | 0.14415 | 1.4243958 | 0.5853312 | 3.466248323 | 0.435608 | 6.177676 | 0.103279 | 0.157141 | 0.194 | NA |
| Number of days/week of moderate physical activity 10+ minutes | 0.900630059 | 0.642483 | 1.2624986 | 0.543618 | 0.1835104 | 0.0246922 | 1.363836542 | 0.119795 | 0.8742395 | 0.5499383 | 1.389782572 | 0.569844 | 13.92891 | 0.530927 | 0.326716 | 0.506 | NA |
| Diabetes mellitus | 1.019479335 | 0.956664 | 1.0864189 | 0.552122 | 0.9398744 | 0.8272008 | 1.067895355 | 0.346197 | 0.9849641 | 0.8934898 | 1.08580345 | 0.760634 | 41.01519 | 0.717705 | 0.156922 | 0.741 | NA |
| Average monthly champagne plus white wine intake | 0.937533174 | 0.750971 | 1.1704418 | 0.568824 | 0.8336945 | 0.5171823 | 1.343909983 | 0.472468 | 1.0297081 | 0.7674184 | 1.381643805 | 0.84526 | 6.204372 | 0.859386 | 0.598234 | 0.869 | NA |
| Chest pain or discomfort | 2.754248058 | 0.074394 | 101.96965 | 0.582429 | 11.937574 | 4.90E-12 | 2.91E+13 | 0.872965 | 1.6908556 | 0.0213654 | 133.8140007 | 0.813814 | 0.560311 | 0.989748 | 0.923965 | 0.995 | NA |
| Current employment status: Looking after home and/or family | 4.581599615 | 0.016859 | 1245.11172 | 0.594554 | 32.710292 | 2.07E-06 | 517795227.7 | 0.689724 | 24.193643 | 0.0185156 | 31613.01218 | 0.384127 | 4.597095 | 0.916419 | 0.81049 | 0.942 | NA |
| Hand grip strength (left) | 0.916299659 | 0.660022 | 1.2720858 | 0.601512 | 4.6211964 | 1.2788415 | 16.69906442 | 0.021151 | 1.0080097 | 0.6127433 | 1.658253429 | 0.974941 | 112.7554 | 0.756207 | 0.011894 | 0.722 | NA |
| Average weekly spirits intake | 0.875618117 | 0.489531 | 1.5662083 | 0.65436 | 2.2630823 | 0.2491392 | 20.55694647 | 0.472479 | 1.1445884 | 0.5100895 | 2.56833846 | 0.743291 | 29.89035 | 0.878253 | 0.387257 | 0.882 | NA |
| Loneliness, isolation | 1.259735076 | 0.071107 | 22.317554 | 0.874895 | 0.5116407 | 4.62E-07 | 567107.6272 | 0.926884 | 2.9717541 | 0.0946397 | 93.31516706 | 0.535696 | 14.82862 | 0.138436 | 0.899429 | 0.163 | NA |

**Supplementary Table 7 Association of weighted and unweighted scores in five domains with PD risk**

|  | Weighted score (Model1) | | | | Not weighted score (Model1) | | | | Not weighted score (Model2) | | | |
| --- | --- | --- | --- | --- | --- | --- | --- | --- | --- | --- | --- | --- |
| Characteristics | Hazard Ratio | 5% CI | 95% CI | P value | Hazard Ratio | 5% CI | 95% CI | P value | Hazard Ratio | 5% CI | 95% CI | P value |
| **Socioeconomic** **status** | | | | | | | | | | | | |
| Favourable | 1.00 |  |  | Reference | 1.00 |  |  | Reference | 1.00 |  |  | Reference |
| Intermediate | 1.11 | 1.02 | 1.21 | 0.0124 | 1.15 | 1.02 | 1.29 | 0.0246 | 1.07 | 0.95 | 1.21 | 0.274626 |
| Unfavourable | 1.20 | 1.10 | 1.30 | 2.59E-05 | 1.77 | 1.49 | 2.11 | 1.45E-10 | 1.34 | 1.12 | 1.60 | 1.36E-03 |
| P for trend |  |  |  | 2.50E-05 |  |  |  | 2.13E-08 |  |  |  | 3.25E-03 |
| **Medical** **history** | | | | | | | | | | | | |
| Favourable | 1.00 |  |  | Reference | 1.00 |  |  | Reference | 1.00 |  |  | Reference |
| Intermediate | 1.21 | 1.11 | 1.33 | 3.99E-05 | 1.24 | 1.14 | 1.34 | 1.78E-07 | 1.15 | 1.06 | 1.25 | 8.91E-04 |
| Unfavourable | 1.56 | 1.45 | 1.68 | < 2e-16 | 1.75 | 1.61 | 1.90 | < 2e-16 | 1.49 | 1.36 | 1.62 | < 2e-16 |
| P for trend |  |  |  | < 2e-16 |  |  |  | < 2e-16 |  |  |  | < 2e-16 |
| **Psychosocial** **factors** | | | | | | | | | | | | |
| Favourable | 1.00 |  |  | Reference | 1.00 |  |  | Reference | 1.00 |  |  | Reference |
| Intermediate | 1.14 | 1.05 | 1.24 | 0.003 | 1.18 | 1.08 | 1.29 | 3.33E-04 | 1.11 | 1.01 | 1.22 | 2.32E-02 |
| Unfavourable | 1.62 | 1.49 | 1.76 | < 2e-16 | 1.57 | 1.45 | 1.69 | < 2e-16 | 1.40 | 1.29 | 1.51 | < 2e-16 |
| P for trend |  |  |  | < 2e-16 |  |  |  | < 2e-16 |  |  |  | 6.21E-06 |
| **Physical** **measures** | | | | | | | | | | | | |
| Favourable | 1.00 |  |  | Reference | 1.00 |  |  | Reference | 1.00 |  |  | Reference |
| Intermediate | 1.34 | 1.23 | 1.46 | 5.26E-11 | 1.27 | 1.15 | 1.40 | 1.51E-06 | 1.18 | 1.07 | 1.31 | 7.75E-04 |
| Unfavourable | 1.75 | 1.56 | 1.97 | < 2e-16 | 1.52 | 1.35 | 1.70 | 5.57E-13 | 1.30 | 1.16 | 1.45 | 1.02E-05 |
| P for trend |  |  |  | < 2e-16 |  |  |  | 3.92E-13 |  |  |  | 6.21E-06 |
| **Lifestyles** | | | | | | | | | | | | |
| Favourable | 1.00 |  |  | Reference | 1.00 |  |  | Reference | 1.00 |  |  | Reference |
| Intermediate | 1.11 | 1.02 | 1.21 | 0.0171 | 1.13 | 1.02 | 1.26 | 0.0217 | 1.11 | 1.00 | 1.24 | 0.056174 |
| Unfavourable | 1.45 | 1.34 | 1.58 | < 2e-16 | 1.42 | 1.30 | 1.54 | < 2e-16 | 1.32 | 1.21 | 1.43 | 5.11E-10 |
| P for trend |  |  |  | < 2e-16 |  |  |  | < 2e-16 |  |  |  | 1.15E-10 |

Model1 adjusted for age, sex, and assessment center. Model2 further adjusts the scores in the five different areas. Figure 4 in the text shows the results of Model 2 based on weighted scores.

**Supplementary Table 8 Two-sample mendelian randomisation analyses for PD to blood assays**

| **Outcome** | **Inverse variance weighted** | | | | **MR Egger** | | | | **Weighted median** | | | |
| --- | --- | --- | --- | --- | --- | --- | --- | --- | --- | --- | --- | --- |
|  | OR | LCI | UCI | P | OR | LCI | UCI | P | OR | LCI | UCI | P |
| Lymphocyte percentage | 0.83 | 0.71 | 0.98 | 0.03 | 0.94 | 0.58 | 1.54 | 0.82 | 0.91 | 0.73 | 1.14 | 0.40 |
| Neutrophill percentage | 1.12 | 1.02 | 1.23 | 0.02 | 0.87 | 0.57 | 1.32 | 0.53 | 1.07 | 0.95 | 1.21 | 0.27 |
| Total bilirubin | 1.10 | 1.01 | 1.21 | 0.04 | 1.04 | 0.77 | 1.41 | 0.82 | 1.06 | 0.94 | 1.20 | 0.33 |

**Supplementary Table 9 Weighted and unweighted PAF and communality of five domains when shifting the unfavorable variables to favourable and moderate ones**

| Categories | PAF | CI_L | CI_U | HR | CI5 | CI95 | P | communality | Weighted PAF |
| --- | --- | --- | --- | --- | --- | --- | --- | --- | --- |
| Socioeconomic status | 1.47% | -0.58% | 3.58% | 1.05 | 0.98 | 1.13 | 0.163 | 20.45% | 0.66% |
| Medical history | 8.15% | 6.04% | 10.31% | 1.34 | 1.24 | 1.44 | 2.83E-15 | 32.41% | 4.64% |
| Psychosocial factors | 11.02% | 8.49% | 13.59% | 1.39 | 1.29 | 1.49 | 2.00E-16 | 39.50% | 6.93% |
| Physical measures | 9.26% | 5.50% | 13.09% | 1.29 | 1.16 | 1.42 | 9.13E-07 | 23.39% | 4.48% |
| Lifestyles | 9.27% | 6.77% | 11.80% | 1.31 | 1.22 | 1.40 | 8.63E-14 | 18.27% | 3.96% |

Overall weighted PAF: 18.72%**.** PAF, population attributable fraction; HR, hazard ratio; CI, confidence interval

**Supplementary Table 10 Weighted and unweighted PAF and communality of five domains when reducing the unfavourable and moderate variables to favourable ones.**

| Categories | PAF | CI_L | CI_U | HR | CI5 | CI95 | P | communality | Weighted PAF |
| --- | --- | --- | --- | --- | --- | --- | --- | --- | --- |
| Socioeconomic status | 6.30% | 1.84% | 10.66% | 1.11 | 1.03 | 1.19 | 0.00584 | 14.20% | 2.37% |
| Medical history | 12.57% | 9.47% | 15.68% | 1.33 | 1.24 | 1.43 | 1.52E-15 | 22.44% | 5.96% |
| Psychosocial factors | 15.03% | 10.40% | 19.52% | 1.27 | 1.17 | 1.36 | 7.36E-10 | 38.62% | 9.34% |
| Physical measures | 18.14% | 12.83% | 23.23% | 1.32 | 1.21 | 1.44 | 1.41E-10 | 34.26% | 10.62% |
| Lifestyles | 12.60% | 7.82% | 17.22% | 1.21 | 1.13 | 1.31 | 4.42E-07 | 19.59% | 5.58% |

Overall weighted PAF: 33.95%. PAF, population attributable fraction; HR, hazard ratio; CI, confidence interval

## Supplementary Figures

**Supplementary Figure1** The correlation among the variables significantly associated with Parkinson's disease


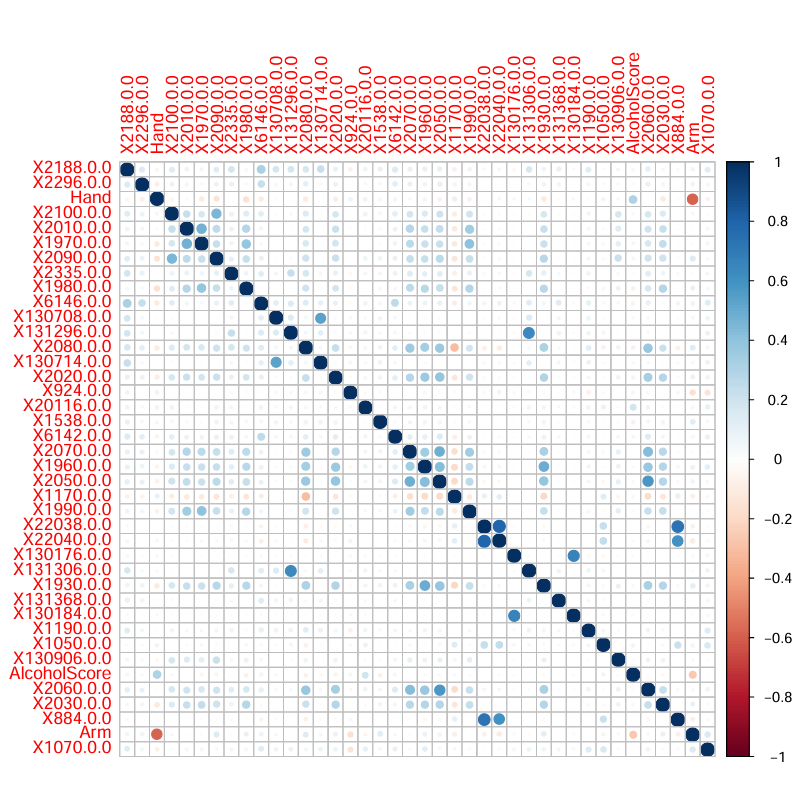

Supplement: Supplementary file 1 [file Data_Sheet_1.docx]
